# UBE2S interacting with TRIM21 mediates the K11-linked ubiquitination of LPP to promote the lymphatic metastasis of bladder cancer

**DOI:** 10.1038/s41419-023-05938-2

**Published:** 2023-07-08

**Authors:** Kanghua Xiao, Shengmeng Peng, Junlin Lu, Ting Zhou, Xuwei Hong, Siting Chen, Guangyao Liu, Hong Li, Jian Huang, Xu Chen, Tianxin Lin

**Affiliations:** 1 Department of Urology, Sun Yat-sen Memorial Hospital, Sun Yat-sen University, Guangzhou, 510120 Guangdong PR China; 2 Guangdong Provincial Key Laboratory of Malignant Tumor Epigenetics and Gene Regulation, Sun Yat-Sen Memorial Hospital, Sun Yat-Sen University, Guangzhou, 510120 Guangdong PR China; 3 Biobank of Sun Yat-sen University Cancer Center, Guangzhou, 510120 Guangdong PR China; 4 Department of Urology, Shantou Central Hospital, Shantou, 515031 PR China; 5 School of Medicine, South China University of Technology, Guangzhou, 510120 Guangdong PR China; 6 BioMed Laboratory, Guangzhou Jingke Biotech Group, Guangzhou, 510120 Guangdong PR China; 7 Guangdong Provincial Clinical Research Center for Urological Diseases, Guangzhou, 510120 Guangdong PR China

**Keywords:** Bladder cancer, Metastasis, Ubiquitylation

## Abstract

Lymphatic metastasis is the most common pattern of bladder cancer (BCa) metastasis and has an extremely poor prognosis. Emerging evidence shows that ubiquitination plays crucial roles in various processes of tumors, including tumorigenesis and progression. However, the molecular mechanisms underlying the roles of ubiquitination in the lymphatic metastasis of BCa are largely unknown. In the present study, through bioinformatics analysis and validation in tissue samples, we found that the ubiquitin-conjugating E2 enzyme *UBE2S* was positively correlated with the lymphatic metastasis status, high tumor stage, histological grade, and poor prognosis of BCa patients. Functional assays showed that *UBE2S* promoted BCa cell migration and invasion in vitro, as well as lymphatic metastasis in vivo. Mechanistically, *UBE2S* interacted with tripartite motif containing 21 (*TRIM21*) and jointly induced the ubiquitination of lipoma preferred partner (*LPP*) via K11-linked polyubiquitination but not K48- or K63-linked polyubiquitination. Moreover, *LPP* silencing rescued the anti-metastatic phenotypes and inhibited the epithelial-mesenchymal transition of BCa cells after *UBE2S* knockdown. Finally, targeting *UBE2S* with cephalomannine distinctly inhibited the progression of BCa in cell lines and human BCa-derived organoids in vitro, as well as in a lymphatic metastasis model in vivo, without significant toxicity. In conclusion, our study reveals that *UBE2S*, by interacting with *TRIM21*, degrades *LPP* through K11-linked ubiquitination to promote the lymphatic metastasis of BCa, suggesting that *UBE2S* represents a potent and promising therapeutic target for metastatic BCa.

## Introduction

As one of the most common malignancies of the genitourinary system, bladder cancer (BCa) had 573,000 newly diagnosed cases and 213,000 deaths worldwide reported in 2020 [1]. Although some novel urinary assays and drugs have been used, the prognosis of muscle-invasive BCa (MIBC) is still poor [2–4]. In addition, BCa patients with lymph node (LN) metastasis of N1, N2 and N3 stages achieved extremely unsatisfactory 5-year disease-free survival rates of 29.3%, 18.2% and 0%, respectively, compared with 81.4% for those without LN metastasis [5]. Lymphatic metastasis is a multistep complex process, including dissemination of cancer cells, transport, and colonization expansion in the LNs [6]. Although some mechanisms of epigenetics have been clarified in BCa [7–9], the molecular mechanism is still not fully understood, especially in posttranslational regulation.

Posttranslational modification (PTM) is an important regulatory process for cellular proteins and is extensively involved in physiological and pathological functions, including apoptosis, innate immunity and cancer [10–14]. As one of the most widely studied PTMs, ubiquitination covalently attaches the 76 amino acid protein ubiquitin (Ub) onto substrates to target proteins [15]. Specifically, Ub activating enzymes (E1s) first activate and transfer Ub to Ub conjugating enzymes (E2s), and then Ub ligases (E3s) transmit the activated Ub moieties to the substrate proteins with the cooperation of E2s. In eukaryotic cells, different polyubiquitination events, which are linked by seven internal lysine residues of Ub (K6, K11, K27, K29, K33, K48 and K63) or via amino-terminal methionine, affect ubiquitinated proteins with specific functions, such as substrate degradation, stabilization, localization and trafficking events [16, 17]. Recently, an increasing number of studies have focused on abnormal ubiquitination in the occurrence and development of cancers [18, 19].

Ubiquitin-conjugating enzyme E2S (*UBE2S*) has been reported to be upregulated in various human cancers and correlates with a poor prognosis. In hepatocellular carcinoma, Zhang *et al*. reported that *UBE2S* interacted with intranuclear *TRIM28* to accelerate the cell cycle by inducing proteasomal degradation of *p27* to support hepatocellular carcinoma development [20]. In prostate cancer, *UBE2S* was responsible for the degradation of *p16* and stabilization of *β-catenin* via K11-linked polyubiquitination to promote bone metastasis [21]. However, the biological mechanisms of *UBE2S* in lymphatic metastasis of BCa remain to be clarified.

In this study, we revealed that *UBE2S* was expressed at high levels in lymphatic metastasis of BCa and positively correlated with an advanced stage and poor prognosis. Inhibition of *UBE2S* impaired the migration, invasion and lymphatic metastasis of BCa cells. Mechanistically, *UBE2S* cooperated with tripartite motif containing 21 (*TRIM21*) to form K11-linked polyubiquitination chains and mediated lipoma preferred partner (*LPP*) degradation, which finally promoted the epithelial-mesenchymal transition (EMT) and enhanced the lymphatic metastasis of BCa. Targeting *UBE2S* with cephalomannine might be a potential therapy to effectively reduce the lymphatic metastasis of BCa.

## Materials and methods

### Human tissue samples

Approval for the use of human tissues was obtained from the ethics committee of Sun Yat-sen University. Five paired fresh bladder specimens were obtained from Sun Yat-sen Memorial Hospital (SYMH). Cohort 1 included 210 BCa tissue specimens and 59 normal urothelial tissues, while Cohort 2 consisted of 59 paired microarray tissues, both of which were obtained from Sun Yat‐sen University Cancer Center between 2000 and 2013 (Supplementary Table S1). All specimens were acquired with written informed consent. Two pathologists independently confirmed each sample by performing hematoxylin and eosin (H&E) staining.

### Immunohistochemistry (IHC)

The IHC procedures and analysis were conducted as previously described [22]. The antibodies are listed in Supplementary Table S2. Briefly, the staining intensity was categorized into four grades: negative (0), weak (1), moderate (2), or strong (3). The staining score (H-score) = staining intensity × staining percentage. *UBE2S* expression was classified as low (H-score <100) or high (H-score ≥100). *LPP* expression was classed as low (H-score <70) or high (H-score ≥70).

### Data mining and analysis

The omics data were obtained from two platforms: The Cancer Genome Atlas (TCGA, https://cancergenome.nih.gov/) and Gene Expression Omnibus (GEO, https://www.ncbi.nlm.nih.gov/geo/) (GSE48277, GSE120736 and GSE31684). Ubiquitination-related genes were downloaded from the Molecular Signature Database (MSigDB, https://www.gsea-msigdb.org/): KEGG UBIQUITIN MEDIATED PROTEOLYSIS. The volcano plot and Venn diagram were drawn using R software.

### Cell lines and cell culture

The human embryonic kidney cell line HEK-293T, normal urothelial cell line SV-HUC-1, and BCa cell lines (T24, UM-UC-3, 5637 and TCCSUP) were purchased from the American Type Culture Collection (ATCC, USA) and cultured as previously described [23]. More details are described in the Supplementary material and methods.

### RNA interference

Small interfering RNA (siRNA) oligonucleotides targeting *UBE2S*, *LPP*, and *TRIM21* and the negative control siRNA (si-Ctrl) were purchased from GenePharma (Shanghai, China) (Table S3). The siRNAs were transfected as previously described [24]. Briefly, 5 μl of siRNAs were coincubated with 3 μl of Lipofectamine RNAiMAX (13778150, Invitrogen, USA) in 200 μl of OPTI-MEM (31985070, Gibco, USA) at room temperature for 20 minutes. Afterwards, the mixture was added to the cells and incubated for 48 h.

### Plasmid transfection and construction of stable cell lines

Short hairpin RNA (shRNA) sequences targeting *UBE2S* were cloned into the pLKO.1-Puro vector, while full-length *UBE2S*, *TRIM21* and *LPP* were cloned into the pCDH-CMV-MCS-EF1-Puro vector. All vectors were purchased from IGE Biotechnology (Guangzhou, China), and their sequences are listed in Table S3. The transient transfection and lentivirus production procedures were described previously [25]. For transient transfection, the plasmids were incubated with Polyethylenimine Linear MW 40000 (PEI-40000, 40816ES02, YEASEN, China) at room temperature for 20 minutes (1 μg of plasmid:2 μl of transfection reagent). For packaging the lentivirus, HEK-293T cells were transfected with the target plasmid, psPAX2 and pMD2. G at a proportion of 4:3:1 along with PEI-40000.

### Quantitative real-time polymerase chain reaction (qPCR)

Total RNA was extracted from fresh tissues with TRIzol Reagent (15596018, Invitrogen, USA) or from cells with an RNA Quick Purification kit (RN001, ESscience, China). RNA (1 μg) was reverse transcribed to complementary DNA templates using a reverse transcription kit (R222-01, Vazyme, China). qPCR was conducted using a Light Cycler 480 system (Roche, Switzerland) with SYBR Green reaction mix (Q711-02, Vazyme, China). The 2^−ΔΔCt^ method (Ct, cycle threshold) was used to calculate relative expression. All qPCR primers are listed in Supplementary Table S4.

### Protein extraction and western blot assay

All proteins were extracted using RIPA lysis buffer supplemented with protease inhibitors (CW2200S, CWBIO, China) and quantified by a BCA Protein Assay Kit (23227, Thermo Fisher, USA). After being electrophoresed, transferred and blocked with 5% skim milk, proteins were incubated with primary and secondary antibodies and visualized using ultrasignal chemiluminescence (4AW011, 4Abio, China). The antibodies are listed in Supplementary Table S2.

### Transwell migration, invasion and wound healing assays

All these experiments were performed as described previously [26]. Photos were captured with an inverted microscope (Olympus, Japan). More details are described in the Supplementary material and methods.

### In vivo popliteal lymphatic metastasis model

All animal experiments were approved by the Sun Yat-sen University Institutional Animal Care and Use Committee (SYSU-IACUC-2023-000232). The procedures were introduced previously [27]. Briefly, 3 ×10^6^ UM-UC-3 cells with stable expression of firefly luciferase were inoculated into the footpads of randomized male BALB/c nude mice (4 weeks old, five mice per group). Usually, all mice were euthanized using cervical dislocation at the 4th week in accordance with the Declaration of Helsinki. The volume of LNs was calculated as follows: LN volume (mm^3^) = length × width^2^ × 0.5. Cephalomannine (S2408, Selleck, China) was dissolved in 5% dimethylacetamide (DMSO), 40% PEG300, 5% TWEEN80 and 50% distilled water. Cephalomannine (200 μl) was injected intraperitoneally into the therapeutic groups, and PBS was used as the control. No statistical method was used to predetermine the sample size for the mouse experiment, which was based on previous experimental observations. The investigator was blinded to the group allocation of the animals during the experiment.

### Immunofluorescence staining

Immunofluorescence staining assays were performed as previously described [28]. More details are described in the Supplementary material and methods.

### Coimmunoprecipitation (co-IP) and mass spectrometry

Wild-type T24 and UM-UC-3 cells were used to investigate the interactions between endogenous *UBE2S*, *TRIM21*, *LPP*, *TRIP13*, *PRMT1*, *UBR5* and *UBE3C*. Briefly, cell lysates were incubated with 2 μg of the corresponding antibodies (Supplementary Table S2) overnight at 4 °C, treated with Protein A/G Magnetic Beads (HY-K0202, MCE, USA) for 2 h at room temperature, and finally detected using western blot or mass spectrometry assays. A protein with ≥2 unique peptides was considered significant.

### In vitro ubiquitination assay

Recombinant proteins, including *UBE1* (Ag8920, Proteintech), *UBE2S* (ATAP00745, AtaGenix), *TRIM21* (Ag28377, Proteintech), *LPP* (P1269, FineTest) and ubiquitin (Ag20753 Proteintech), were purchased, while ubiquitin-K11R protein was expressed by a cell-free protein expression kit (PLDK001, PLD technology) according to the manufacturer’s instructions. This assay was performed according to the report of Jixi et al. [29]. Briefly, a 60 μl conjugation reaction containing 5 μg ubiquitin or ubiquitin-K11R, 5 μg *LPP*, 1 μg *UBE1*, 1 μg *UBE2S*, 1 μg *TRIM21*, 5 mM ATP (D7378, Beyotime) and 5 mM MgCl2 (ST269, Beyotime) was incubated at 37 °C for 1 h. Reactions were terminated by adding 4× loading buffer and subsequently analyzed by western blot with an antibody against LPP (25045-1-AP, Proteintech).

### Establishment of human BCa organoids

Four fresh BCa tissues were acquired with informed consent and transported in tissue storage solution (130-100-008, Miltenyi, Germany). The clinical information of the patients is described in Supplementary Table S5. More details are described in the Supplementary material and methods. The contents of culture medium are provided in Supplementary Table S6.

### Statistical analysis

Data from at least three independent experiments are presented as the means ± standard deviations (means ± SDs). Unpaired or paired Student’s two-tailed *t* test was used to analyze the differences between two groups, whereas one-way or two-way analysis of variance (ANOVA) was performed to compare the differences among at least three groups. All statistical assessments and plotting were executed using Statistical Product and Service Solutions (SPSS, version 24.0) and GraphPad Prism 7.0 software. Statistical significance was considered as *P < 0.05, **P < 0.01.

## Results

### *UBE2S* positively correlates with lymphatic metastasis and poor prognosis of BCa

**Fig. 1:**
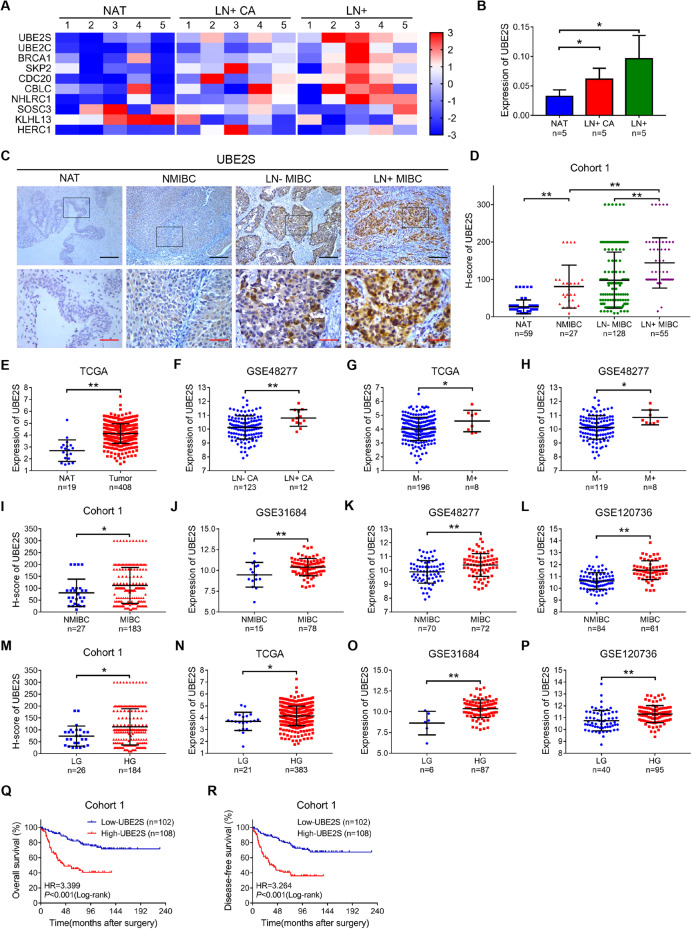
*UBE2S* positively correlates with lymphatic metastasis and poor prognosis in bladder cancer (BCa). **A** Heatmap of 10 dysregulated expressed ubiquitination-related genes in NAT, LN+ CA and LN+, as detected by qPCR. NAT, normal adjacent tissues; LN+ CA, BCa tissues with lymphatic metastasis; LN+, metastatic LNs. **B** Histogram showing the analysis of *UBE2S* mRNA expression in 5 paired BCa tissues detected by qPCR. **C** Representative images of immunohistochemistry staining for *UBE2S* expression in Cohort 1, which consisted of 59 NAT and 210 BCa tissues. Scale bars: black, 200 μm; red, 50 μm. **D** Protein levels of *UBE2S* in Cohort 1. **E** The relative mRNA expression of UBE2S in NAT and BCa tissues from the TCGA database. **F** Expression of *UBE2S* in LN− CA and LN + CA in the GSE48277 database. LN- CA, BCa tissues without lymphatic metastasis. **G**, **H** Comparison of *UBE2S* expression in BCa tissues without metastasis (M−) and with metastasis (M+) from TCGA and GSE48277 databases. **I**–**L** Comparison of *UBE2S* expression in NMIBC and MIBC tissues from Cohort 1, GSE31684, GSE48277 and GSE120736 databases. NMIBC nonmuscle-invasive bladder cancer, MIBC muscle-invasive bladder cancer. **M**–**P** Comparison of *UBE2S* expression in LG and HG BCa tissues from Cohort 1, TCGA, GSE31684 and GSE120736 databases. LG low-grade, HG high-grade. **Q**, **R** Kaplan‒Meier curves showing the overall survival (OS) and disease-free survival (DFS) of BCa patients with high *UBE2S* expression and low *UBE2S* expression in Cohort 1). *P < 0.05 and **P < 0.01.

We first performed a bioinformatics analysis using the TCGA-BLCA database to identify key ubiquitin enzymes in lymphatic metastasis of BCa (Supplementary Fig. S1A). Notably, *UBE2S* was one of the most significantly upregulated genes in both BCa tissues with lymphatic metastasis (LN+ CA) and metastatic LNs (LN+) compared with normal adjacent tissues (NAT) (Fig. 1A, B & Supplementary Fig. S1B). Furthermore, by intersecting the 1390 differentially expressed metastasis-related genes, we confirmed *UBE2S* as the highly expressed gene associated with both ubiquitination and metastasis in TCGA-BLCA (Supplementary Fig. S1C).

Subsequently, we detected *UBE2S* protein levels in cohort 1 using IHC assays and found that *UBE2S*, which localized in both the nucleus and cytoplasm, was nearly absent in NAT and exhibited stepwise increases from nonmuscle-invasive bladder cancer (NMIBC) to muscle-invasive bladder cancer (MIBC) without lymphatic metastasis (LN− MIBC) to MIBC with lymphatic metastasis (LN+ MIBC) (Fig. 1C, D). Consistently, higher *UBE2S* expression was found in BCa tissues compared to NAT in TCGA database (Fig. 1E), and in LN+ CA than in BCa tissues without lymphatic metastasis (LN-CA) in GSE48277 database (Fig. 1F). In addition, high *UBE2S* expression was associated with distant metastasis (M+) in BCa patients compared to those without metastasis (M-) in both TCGA and GSE48277 databases (Fig. 1G, H). We also found that *UBE2S* was upregulated not only in MIBC of the Cohort 1, GSE31684, GSE48277 and GSE120736 datasets (Fig. 1I–L) but also in high-grade (HG) BCa tissues compared with low-grade (LG) BCa tissues in the Cohort 1, TCGA, GSE31684 and GSE120736 datasets (Fig. 1M–P). Similarly, high *UBE2S* expression was closely related to a higher histological grade and more advanced T stage, N stage and M stage in Cohort 1 (*P* = 0.004, 0.008, <0.001 and 0.030, respectively) (Supplementary Table S7).

As shown in Fig. 1Q–R, the Kaplan‒Meier survival analysis revealed that BCa patients with high *UBE2S* expression experienced both lower overall survival (OS) and disease-free survival (DFS) rates than those with low *UBE2S* expression in cohort 1. Further univariate and multivariate Cox regression analyses confirmed high *UBE2S* expression as a risk factor for both the OS (*P* < 0.001) and DFS (*P* < 0.001) of BCa patients (Supplementary Table S8). Taken together, high *UBE2S* expression was not only related to lymphatic metastasis and advanced tumor and histological grades but also predicted a poor prognosis for BCa patients.

### *UBE2S* knockdown inhibits BCa migration and invasion in vitro and lymphatic metastasis in vivo

**Fig. 2:**
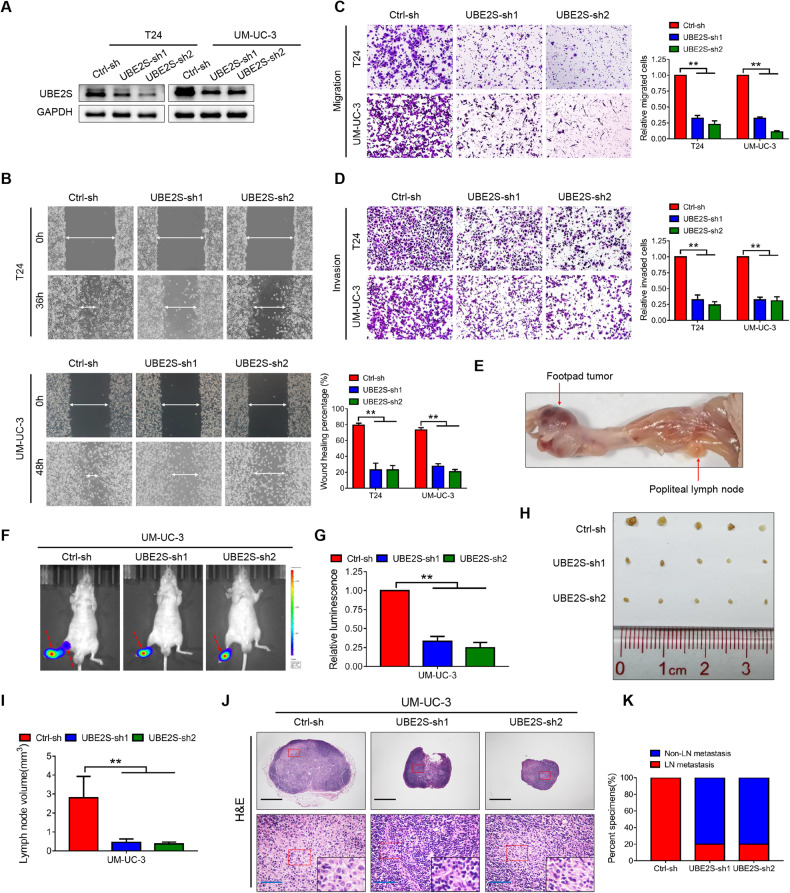
*UBE2S* knockdown inhibits BCa migration and invasion in vitro and lymphatic metastasis in vivo. **A** Western blot analysis of *UBE2S* protein expression levels in *UBE2S*-silenced BCa cells. Representative images and quantitative analysis of wound healing (**B**), transwell migration (**C**) and invasion (**D**) assays in *UBE2S*-silenced BCa cells. **E** Representative image of the popliteal lymphatic metastasis model. Representative bioluminescence images (**F**) and quantitative analysis (**G**) of popliteal metastatic LNs from nude mice. LNs lymph nodes. Representative image of popliteal LNs (**H**) and quantitative analysis of their volumes (**I**). **J** Representative images of H&E staining in popliteal LNs from each group. Scale bars: black, 500 μm; blue, 50 μm. **K** Percentages of lymphatic metastasis in each group. *P < 0.05 and **P < 0.01.

We first constructed stable *UBE2S*-knockdown and *UBE2S*-overexpressing cells using T24 and UM-UC-3 cell lines (with moderate *UBE2S* expression, Supplementary Fig. S2A) through lentiviral transfection, the efficiency of which was confirmed by western blot assay (Fig. 2A & Supplementary Fig. S2B). As shown in the wound healing and transwell migration assays, the migratory speed and number of BCa cells were substantially decreased in the *UBE2S*-knockdown groups (Fig. 2B, C), while the opposite was observed in the *UBE2S*-overexpressing groups (Supplementary Fig. S2C, D). Meanwhile, the invasive capability of BCa cells was significantly suppressed when *UBE2S* was silenced (Fig. 2D) but enhanced after ectopic overexpression of *UBE2S* (Supplementary Fig. S2E). In addition, synchronous cell apoptosis assays revealed no significant difference (Supplementary Fig. S3), further confirming the metastasis-promoting effects of *UBE2S*.

An in vivo popliteal LN model was established using stable *UBE2S*-knockdown UM-UC-3 cells to determine the biological functions of *UBE2S* (Fig. 2E). As revealed in Fig. 2F, G, a significant decrease in luminescence of the popliteal LNs was observed in the *UBE2S*-knockdown groups compared to the control group. Furthermore, *UBE2S* silencing distinctly reduced the volume of popliteal LNs (Fig. 2H, I). Meanwhile, the percentage of lymphatic metastasis in the *UBE2S*-knockdown groups was noticeably decreased to 20% compared to 100% in the matched group based on H&E staining of the popliteal LNs (Fig. 2J, K). Collectively, our findings revealed that *UBE2S* increased migration and invasion in vitro and lymphatic metastasis of BCa cells in vivo.

### *UBE2S* interacts with *TRIM21* and *LPP* in both the nucleus and cytoplasm of BCa

**Fig. 3:**
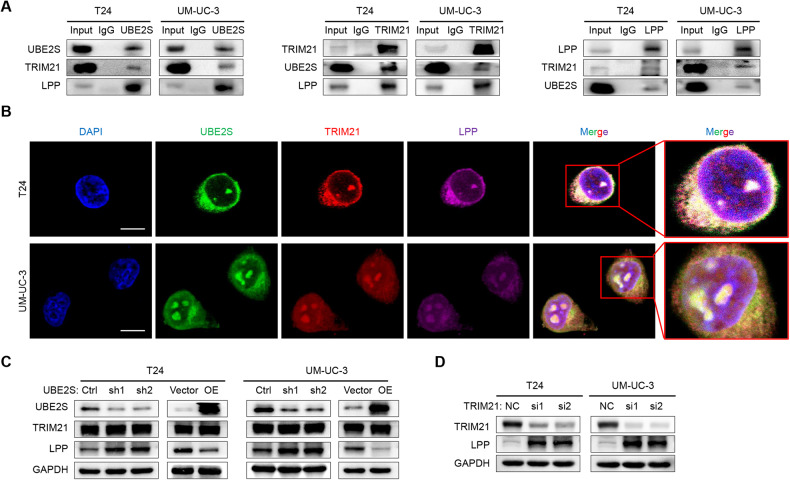
*UBE2S* interacts with *TRIM21* and *LPP* in both the nucleus and cytoplasm of BCa. **A** The endogenous interaction among *UBE2S*, *TRIM21* and *LPP* in BCa cells was determined by performing co-IP and western blot assays. Anti-*UBE2S* (14115-1-AP, Proteintech), anti-*TRIM21* (12108-1-AP, Proteintech) and anti-*LPP* (25045-1-AP, Proteintech) were used for Co-IP (each 2 μg). **B** Representative immunofluorescence images of *UBE2S, TRIM21* and *LPP* colocalization in the nucleus and cytoplasm of BCa cells. Scale bars: white, 25 μm. DAPI was used to label the cell nucleus. **C** *UBE2S*, *TRIM21* and *LPP* protein levels in *UBE2S*-silenced or *UBE2S*-overexpressing BCa cells. **D** *TRIM21* and *LPP* protein levels in *TRIM21*-silenced BCa cells.

We performed immunoprecipitation (IP) and mass spectrometry assays of wild-type T24 and UM-UC-3 cells with a *UBE2S* antibody to investigate the potential mechanisms of *UBE2S* in lymphatic metastasis. We found ten E3 enzymes (Supplementary Table S9), among which *TRIM21, UBR5* and *UBE3C* have been widely reported to be involved in tumor metastasis [30–35]. In addition, we selected three more metastasis-related genes (*TRIP13*, *LPP* and *PRMT1*) for further study from 27 potential substrate proteins of *UBE2S* (Top 10 listed in Supplementary Table S9). Finally, we confirmed the interactions among *UBE2S*, *TRIM21* and *LPP* by co-IP and western blot assays (Fig. 3A & Supplementary Fig. S4), while *UBE2S* did not interact with *UBR5, UBE3C, TRIP13 or PRMT1* in BCa cells (Supplementary Fig. S5). Moreover, immunofluorescence assays revealed that *UBE2S*, *TRIM21* and *LPP* highly colocalized in the nucleus and cytoplasm of BCa cells (Fig. 3B).

In addition, *LPP* protein was increased in *UBE2S-*knockdown BCa cells but decreased in *UBE2S*-overexpressing cells, while *TRIM21* expression was not affected (Fig. 3C). Similarly, *TRIM21* knockdown in BCa cells increased *LPP* protein levels (Fig. 3D). In addition, we also performed qPCR assays and found that neither knockdown of *UBE2S* nor *TRIM21* obviously increased the mRNA levels of *LPP* (Supplementary Fig. S6), indicating that *UBE2S* and *TRIM21* regulated *LPP* mainly through posttranslational modification. Taken together, *UBE2S* interacting with *TRIM21* regulated the stability of *LPP*.

### *UBE2S* interacts with *TRIM21* to degrade *LPP* via K11-linked ubiquitination

**Fig. 4:**
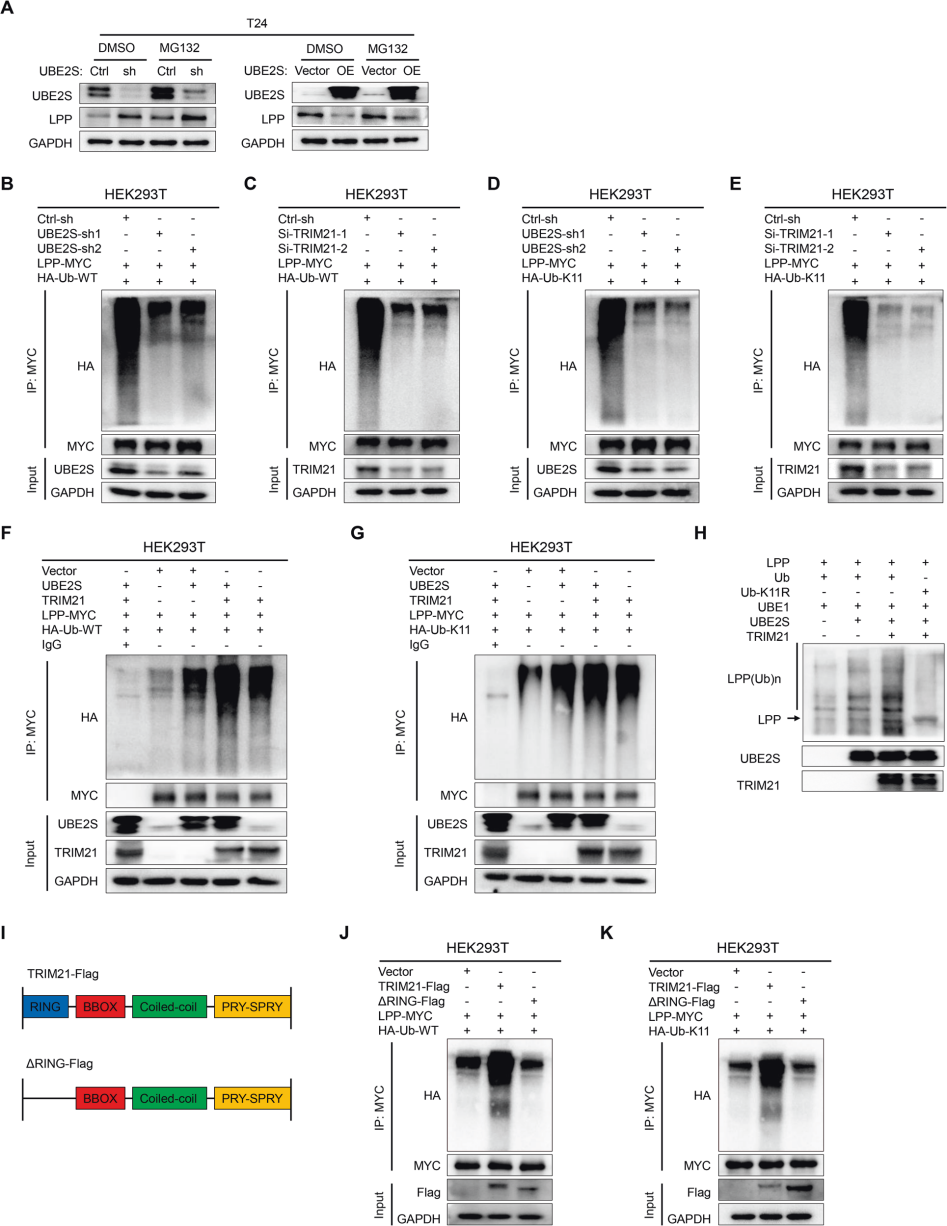
*UBE2S* interacts with *TRIM21* to degrade *LPP* by inducing K11-linked ubiquitination. **A** *LPP* protein levels in different groups of BCa cells after treatment with MG132 (10 μM, 12 h) or DMSO. The total (**B**, **C**) and K11-linked (**D**, **E**) ubiquitination levels of *LPP* in *UBE2S*-knockdown or *TRIM21*-knockdown HEK293T cells transfected with corresponding plasmids (48 h) and treated with MG132 (10 μM, 12 h). WT wild type. The total (**F**) and K11-linked (**G**) ubiquitination levels of *LPP* in *UBE2S*- and *TRIM21*-overexpressing HEK293T cells transfected with corresponding plasmids (48 h) and treated with MG132 (10 μM, 12 h). **H** An in vitro ubiquitination assay was performed in the presence of *LPP*, ubiquitin (Ub), Ub-K11R, *UBE1*, *UBE2S* and *TRIM21*. The reaction was analyzed by western blotting with an anti-*LPP* antibody (25045-1-AP, Proteintech). **I** Schematic diagram of *TRIM21* and its truncated mutant. The total (**J**) and K11-linked (**K**) ubiquitination levels of *LPP* in full-length *TRIM21* and *TRIM21*-ΔRING HEK293T cells transfected with the corresponding plasmids (48 h) and treated with MG132 (10 μM, 12 h).

We next determined whether *UBE2S*-induced *LPP* downregulation occurred in a ubiquitin‒proteasome-dependent manner. As shown in Fig. 4A, the difference in *LPP* protein levels was attenuated after treatment with MG132 (a proteasome inhibitor), indicating that *UBE2S* mediated *LPP* degradation via ubiquitination. Previous studies reported that K11-linked polyubiquitination by *UBE2S* or other ubiquitin-related enzymes mediated the degradation of various substrate proteins [21, 36, 37]. Meanwhile, K48- and K63-linked polyubiquitination were also associated with the degradation of substrates [38]. Therefore, we performed in vivo ubiquitination assays in HEK293T cells and found that knockdown of *UBE2S* markedly decreased both the total and K11-polyubiquitination but not K48- or K63-linked polyubiquitination of MYC-tagged-*LPP*, which was more obvious in *TRIM21*-knockdown HEK293T cells (Fig. 4B–E & Supplementary Fig. S7A–D). Simultaneously, overexpression of *UBE2S* and *TRIM21* in HEK293T cells obviously increased the ubiquitination of *LPP* through K11-linked ubiquitination but not K48- or K63-linked ubiquitination (Fig. 4F-G & Supplementary Fig. S7E, F). Furthermore, a reconstituted in vitro ubiquitination assay demonstrated that *UBE2S* and *TRIM21* successfully assembled polyubiquitin chains on *LPP*, while the polyubiquitin signal was distinctly diminished after the K11 mutant of Ub (Ub-K11R) (Fig. 4H), which strongly suggested that *UBE2S* combined with *TRIM21* mediated K11-linkage formation on *LPP*.

*TRIM21* contains four domains, including the RING-finger, B-box (BBOX), coiled-coil and PRY-SPRY domains [39]. Numerous studies have disclosed that the RING-finger domain of *TRIM21* is responsible for the function of its E3 ligase [40, 41]. Therefore, we constructed a plasmid of truncated *TRIM21* that lacked the RING domain (ΔRING) (Fig. 4I) and found that ΔRING dramatically decreased the total and K11-linked ubiquitination levels of LPP (Fig. 4J, K), which confirmed that the RING domain of *TRIM21* was necessary for the ubiquitination of *LPP*. In summary, *UBE2S* interacted with *TRIM21*, and both proteins facilitated the degradation of *LPP* by inducing K11-linked polyubiquitination.

### *LPP* suppresses the prometastatic effects of *UBE2S* on BCa

**Fig. 5:**
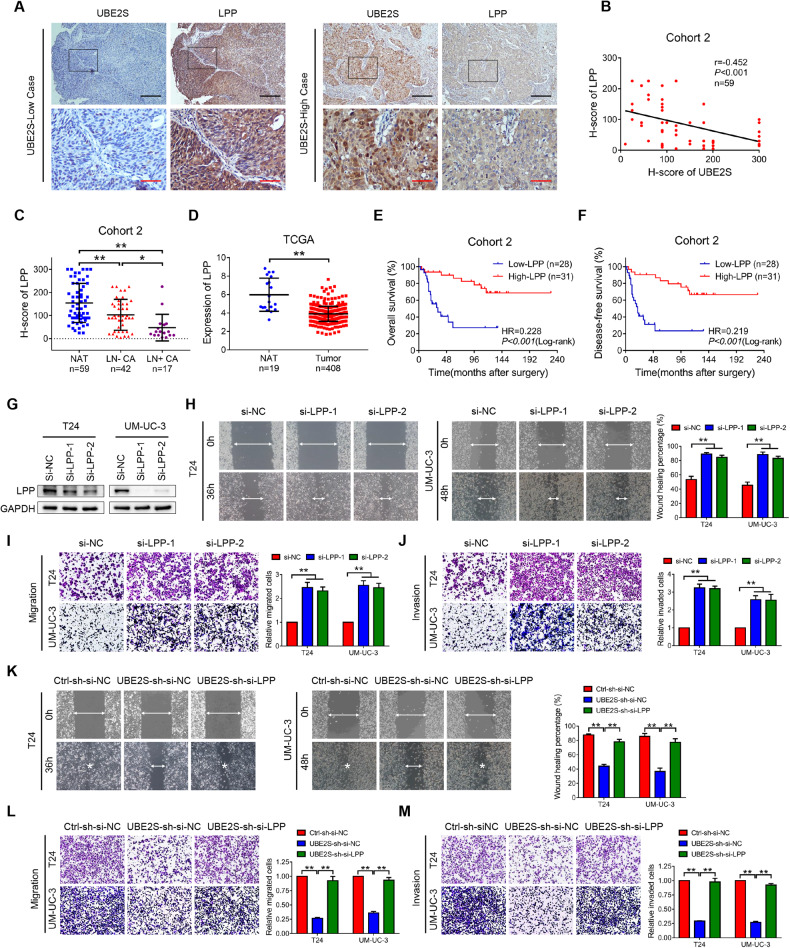
*LPP* reverses the metastasis-promoting effects of *UBE2S*. **A** Representative images of *UBE2S* and *LPP* immunohistochemistry staining in BCa tissues. Scale bars: black, 200 μm; red, 50 μm. **B** Pearson’s correlation analysis of *UBE2S* and *LPP* protein expression in Cohort 2, which consisted of 59 paired BCa tissues. **C** *LPP* protein levels in Cohort 2. NAT normal adjacent tissues; LN− CA, BCa tissues without lymphatic metastasis; LN+ CA, BCa tissues with lymphatic metastasis. **D** Relative *LPP* mRNA expression in NAT and BCa tissues from the TCGA database. Kaplan‒Meier curves showing the overall survival (**E**) and disease-free survival (**F**) of BCa patients with high *LPP* expression and low *LPP* expression in cohort 2. **G** *LPP* protein levels in *LPP*-silenced BCa cells, as detected by western blot assays. Representative images and quantitative analysis of wound healing (**H**), transwell migration (**I**) and invasion (**J**) assays using *LPP*-silenced BCa cells. Representative images and quantitative analyses of wound healing (**K**), transwell migration (**L**) and invasion (**M**) assays of *UBE2S*-silenced BCa cells combined with *LPP* knockdown. *P < 0.05 and **P < 0.01.

We performed IHC assays in Cohort 2 to explore the clinical relevance of *UBE2S*–*LPP* regulation in BCa patients. As displayed in Fig. 5A, B, a significant negative correlation was observed between the expression of *UBE2S* and *LPP* (*r* = −0.452, *P* < 0.001). Chi-square tests indicated that *LPP* expression was negatively related to N and M stages (*P* < 0.001 and 0.030, respectively, Supplementary Table S10). More importantly, we discovered that *LPP* expression was gradually downregulated from NAT to LN-CA to LN + CA (Fig. 5C). Similarly, *LPP* was expressed at high levels in NAT compared with bladder tumors in the TCGA cohort (Fig. 5D). Kaplan‒Meier survival analysis of cohort 2 revealed that BCa patients with low *LPP* expression had a poor prognosis with lower OS and DFS rates (Fig. 5E, F). In addition, univariate and multivariate analyses indicated low *LPP* expression as a risk factor for both the OS and DFS of BCa patients (Supplementary Table S11).

We next evaluated whether *UBE2S* exerted its oncogenic function in an *LPP*-dependent manner by transfecting siRNAs, the efficiency of which was detected by western blot assays (Fig. 5G). Interestingly, *LPP* silencing distinctly increased the migration and invasion of BCa cells (Fig. 5H–J). Consistent with our hypothesis, *LPP* knockdown effectively rescued the inhibition of the migratory and invasive abilities of BCa cells induced by silencing *UBE2S* (Fig. 5K–M). In addition, silencing *TRIM21* diminished the migratory ability of BCa cells, as revealed in the transwell migration assays (Supplementary Fig. S8). Taken together, our results revealed that *LPP* functioned as a tumor suppressor in BCa and reversed the metastasis-promoting effects of *UBE2S*.

### *UBE2S* promotes the EMT of BCa cells in an *LPP*-dependent manner

**Fig. 6:**
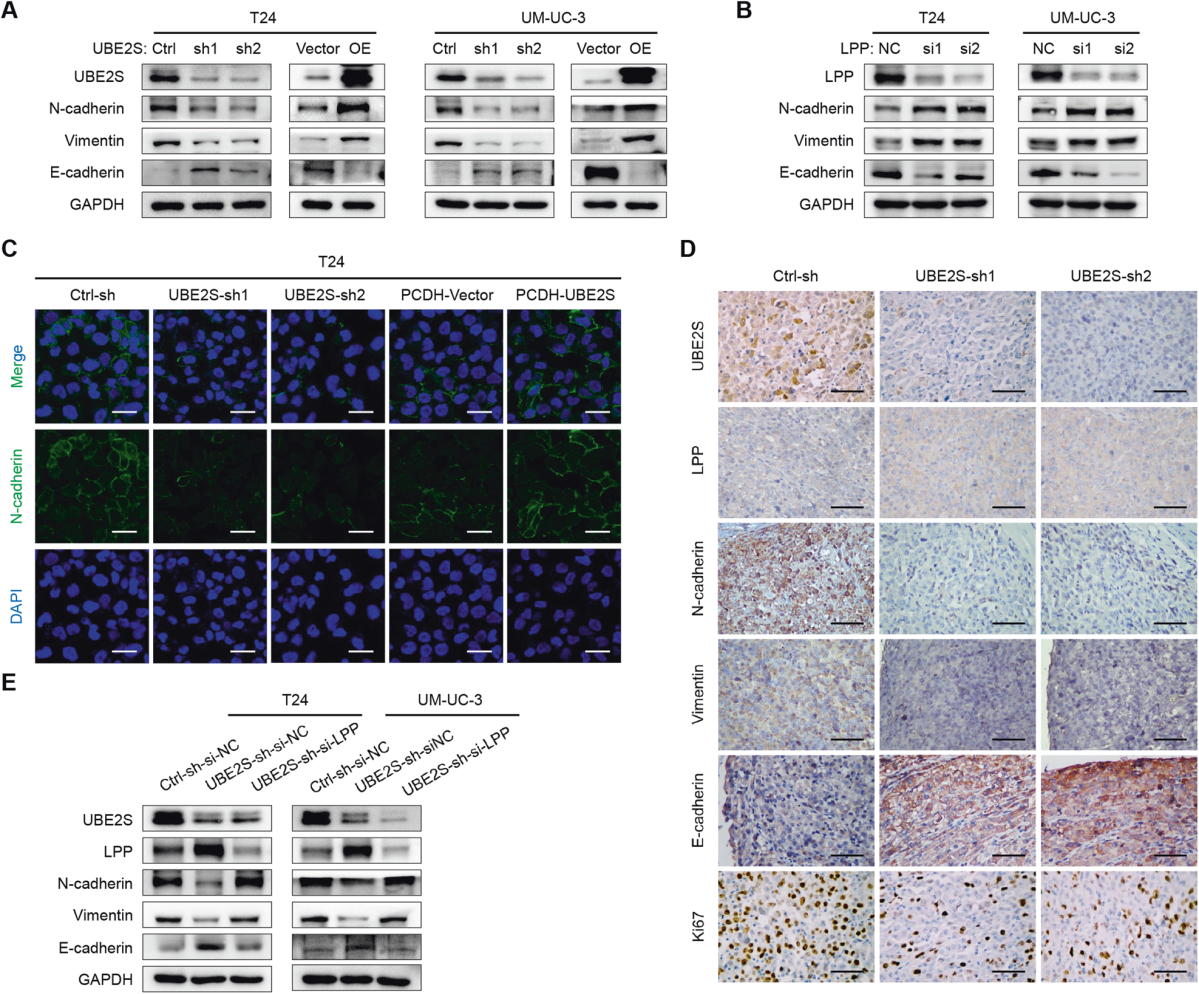
*UBE2S* promotes EMT in BCa cells in an *LPP*-dependent manner. Protein levels of EMT markers (*N-cadherin*, *Vimentin* and *E-cadherin*) in *UBE2S*-silenced and *UBE2S*-overexpressed (**A**) or *LPP*-silenced (**B**) BCa cells, as detected by western blot assays. **C** Representative images of immunofluorescence staining showing *N-cadherin* expression in *UBE2S*-silenced and *UBE2S*-overexpressing T24 cells. Scale bars: white, 20 μm. DAPI was used to label the cell nucleus. **D** Representative images of immunohistochemistry staining showing *UBE2S*, *LPP*, *N-cadherin*, *Vimentin*, *E-cadherin* and *Ki-67* expression in footpad tumors. Scale bars: black, 50 μm. **E** The protein levels of EMT markers and *LPP* in *UBE2S*-silenced BCa cells combined with *LPP* knockdown, as detected using western blot assays.

Previous studies have documented that *LPP* is closely related to invadopodia formation and EMT in a variety of cancers [42]. Therefore, we performed western blot assays to detect the expression of EMT markers. The mesenchymal markers *N-cadherin* and *Vimentin* were downregulated, whereas the epithelial marker *E-cadherin* was upregulated in *UBE2S*-knockdown BCa cells, and vice versa, suggesting that *UBE2S* enhanced the metastasis of BCa cells by promoting the process of EMT (Fig. 6A). Additionally, *LPP* knockdown increased the protein levels of mesenchymal markers but decreased epithelial markers in BCa cells (Fig. 6B). Furthermore, immunofluorescence staining indicated that silencing *UBE2S* impaired EMT, while overexpressing *UBE2S* accelerated EMT (Fig. 6C & Supplementary Fig. S9). In addition, we detected EMT markers in footpad tumor tissues derived from the *UBE2S*-knockdown in vivo model using IHC assays. As shown in Fig. 6D, compared to the control group, the expression of *UBE2S*, *N-cadherin*, *Vimentin* and *Ki-67* was evidently diminished, while the expression of *LPP* and *E-cadherin* was obviously increased in the *UBE2S*-knockdown groups. We then performed a rescue experiment by silencing *LPP* expression in UBE2S-knockdown BCa cells. Obviously, the inhibition of EMT by *UBE2S* knockdown was significantly recovered after the suppression of *LPP* (Fig. 6E). These data validated that *UBE2S* facilitated EMT in BCa cells in an *LPP*-dependent manner.

### Cephalomannine pharmacologically inhibits *UBE2S* and blocks the lymphatic metastasis of BCa cells

**Fig. 7:**
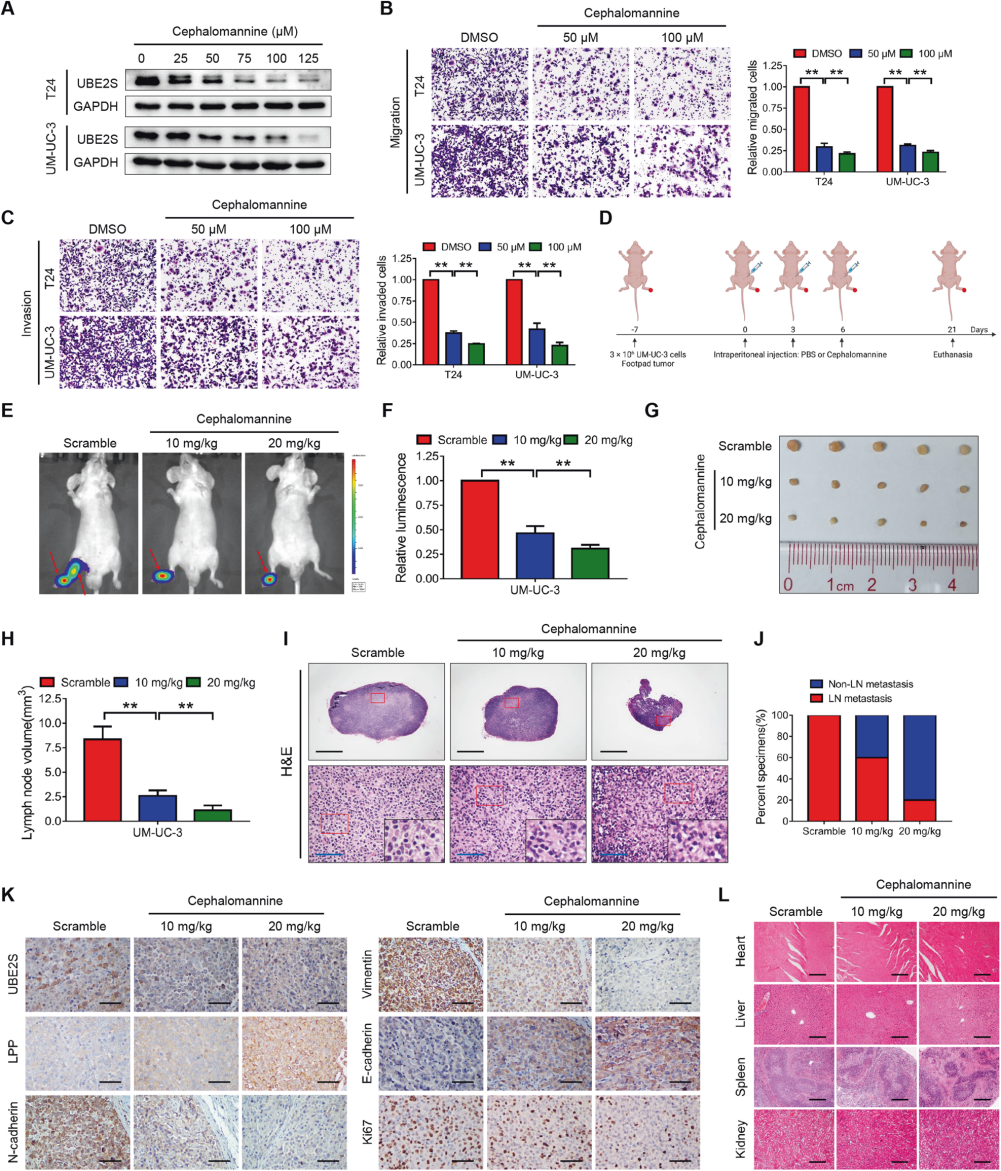
Cephalomannine pharmacologically inhibits *UBE2S* and blocks the lymphatic metastasis of BCa cells. **A** *UBE2S* protein levels in BCa cells treated with various concentrations of cephalomannine for 48 h, as determined by western blot assays. Representative images and quantitative analyses of transwell migration (**B**) and invasion (**C**) assays of BCa cells treated with cephalomannine at the indicated concentrations. **D** Schematic illustration of the intraperitoneal injection of PBS or cephalomannine in a UM-UC-3 popliteal lymphatic metastasis model. Representative bioluminescence images (**E**) and quantitative analysis (**F**) of popliteal metastatic LNs from nude mice. LNs lymph nodes. Representative images of popliteal LNs (**G**) and quantitative analysis of their volumes (**H**). **I** Representative images of H&E staining in popliteal LNs from each group. Scale bars: black, 500 μm; blue, 50 μm. **J** Percentages of lymphatic metastasis in each group. **K** Representative images of IHC staining showing *UBE2S*, *LPP*, *N-cadherin*, *Vimentin*, *E-cadherin* and *Ki-67* expression in footpad tumors of the indicated groups. Scale bars: black, 50 μm. **L** Representative images of H&E staining in the major organs treated with the indicated concentrations of cephalomannine. Scale bars: black, 50 μm. **P < 0.01.

We next explored the functional roles of cephalomannine, a small-molecule compound that inhibits *UBE2S* promoter activity [20]. As shown in Fig. 7A, *UBE2S* expression was gradually decreased in a dose-dependent manner in BCa cells treated with cephalomannine. Transwell migration and invasion assays revealed strong inhibitory effects of cephalomannine on BCa cells (Fig. 7B, C). A popliteal lymphatic metastasis model showed that both the relative luminescence and popliteal LN volume were visibly decreased in the cephalomannine-treated groups in a dose-dependent manner compared with the scrambled control group (Fig. 7D–H). Moreover, the lymphatic metastasis rate was reduced from 100% in the matched group to 60% and 20% in the groups treated with 10 mg/kg and 20 mg/kg cephalomannine, respectively (Fig. 7I, J). In particular, IHC assays of footpad tumors revealed that cephalomannine reduced the expression of *UBE2S*, *N-cadherin*, *vimentin* and *Ki-67* but increased the expression of *LPP* and *E-cadherin* in a concentration-dependent manner (Fig. 7K). In addition, no significant histological alterations were observed in the heart, liver, spleen and kidney between the therapeutic groups and the control group (Fig. 7L). Meanwhile, a blood analysis showed no significant differences among the three groups, indicating that cephalomannine had no apparent toxicity (Supplementary Table S12). Overall, these data indicated that cephalomannine dose-dependently suppressed BCa cell metastasis both in vitro and in vivo by targeting *UBE2S*.

### Cephalomannine inhibited the growth and metastasis of human BCa-derived organoids

**Fig. 8:**
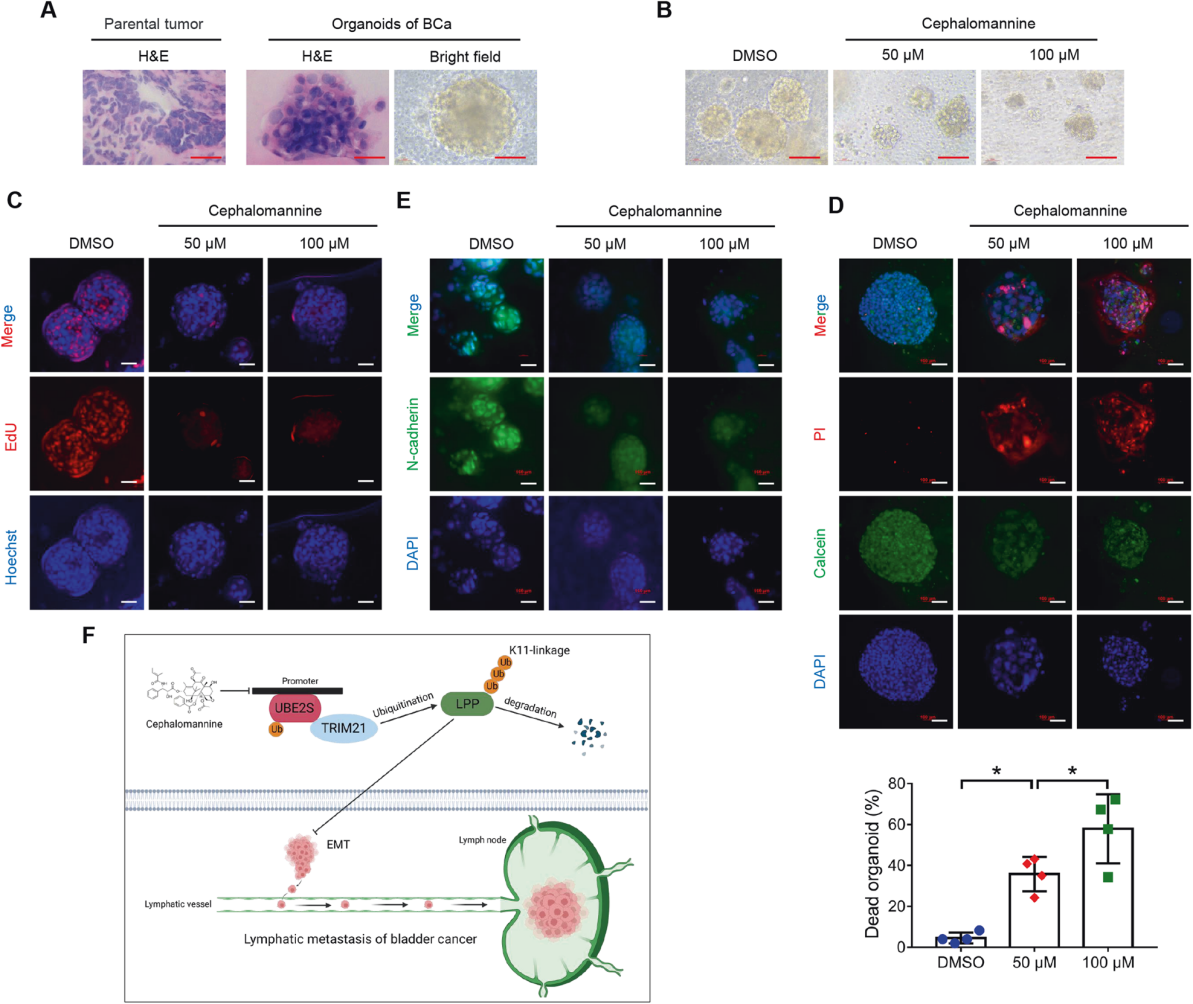
Cephalomannine inhibits the growth and metastasis of organoids derived from human BCa. **A** Representative images of H&E staining of parental tumors and PDOs and bright-field images of organoids. PDOs patient-derived organoids. Scale bars: red, 100 μm. **B** Bright-field images of BCa organoids treated with the indicated concentrations of cephalomannine for 96 h. Scale bars: red, 100 μm. **C** Measurement of the organoids in S phase using EdU assays. Scale bars: white, 100 μm. Hoechst was used to label the cell nucleus. **D** Representative images and quantitative analysis of cell viability in four BCa organoids treated with the indicated concentrations of cephalomannine for 96 h. PI was used to label the dead cells, while calcein was used to label the living cells. Scale bars: white, 100 μm. *P < 0.05 and **P < 0.01. **E** Representative immunofluorescent images showing *N-cadherin* expression in BCa organoids treated with the indicated concentrations of cephalomannine for 96 h. Scale bars: white, 100 μm. **F** Schematic diagram depicting the underlying mechanism of *UBE2S* function in lymphatic metastasis of BCa.

For an in-depth investigation of the clinical value of cephalomannine in BCa, we established four patient-derived organoids (PDOs) (Fig. 8A). Functionally, both bright-field images and EdU assays provided evidence that cephalomannine disturbed the growth and proliferation of BCa organoids in a dose-dependent manner (Fig. 8B, C & Supplementary Fig. S10A). In addition, BCa organoids were significantly more sensitive to cephalomannine, with mean inhibition rates of 36% and 58% in the cephalomannine groups (50 μM and 100 μM, respectively) compared with 5% in the DMSO group (Fig. 8D & Supplementary Fig. S10B). Furthermore, we also found that cephalomannine decreased the expression of the mesenchymal marker *N-cadherin*, suggesting that cephalomannine blocked EMT in BCa organoids (Fig. 8E & Supplementary Fig. S10C). Taken together, these results confirmed the efficacy of cephalomannine in suppressing the growth of human BCa organoids.

## Discussion

Tumor metastasis, particularly lymphatic metastasis, is the main cause of death in BCa, since no sufficient targeted treatments have been developed. Thus, a clinical need exists to elucidate the molecular mechanism and develop novel targeted therapies for lymphatic metastasis of BCa. In the present study, we identified a novel biomarker and therapeutic target termed *UBE2S* that is involved in the LN metastasis of BCa. Mechanistically, *UBE2S* directly interacted with *TRIM21* to enhance the metastatic capacities and EMT of BCa cells by degrading *LPP* via K11-linked ubiquitination. Furthermore, targeting UBE2S with cephalomannine was a promising strategy to prevent the lymphatic metastasis of BCa cells (Fig. 8F).

The ubiquitin proteasome system (UPS), which affects nearly all pivotal functions in tumor cells, is crucial for maintaining protein homeostasis [43]. As part of the UPS, *UBE2S* is reported to promote tumor development via the degradation or stabilization of various proteins. However, to the best of our knowledge, no study has reported the relationship between *UBE2S* and lymphatic metastasis. In the present study, we discovered that *UBE2S* expression was closely correlated with lymphatic metastasis and served as an independent prognostic factor for BCa patients. In addition, *UBE2S* enhanced BCa cell migration and invasion in vitro and lymphatic metastasis in vivo. Taken together, *UBE2S* may serve as a novel biomarker for LN metastasis and the prognosis of BCa.

Lipoma preferred partner (*LPP*), a member of the zyxin family of LIM proteins, functions as an actin-binding protein and transcriptional coactivator and plays an essential role in metastatic processes [44]. However, its functions strongly depend on the context. For example, in invasive endometrial carcinoma, *LPP* transcriptionally regulates *ETV5* to activate *Zeb1*, which later suppresses *E-cadherin* to promote EMT [45]. In contrast, *LPP* directly regulates *MMP15* to degrade *N-cadherin*, which results in inhibition of EMT in lung cancer [44]. In our study, *LPP* suppressed metastasis-related phenotypes and EMT and served as a tumor suppressor in BCa. Furthermore, knockdown of *LPP* effectively rescued the inhibition of metastasis in *UBE2S*-silenced cells, highlighting that *UBE2S* regulated the malignant progression of BCa in an *LPP*-dependent manner.

Tripartite motif containing 21 (*TRIM21*) was first discovered for its antiviral function in innate immunity but has recently attracted increasing attention in cancer metabolism and tumorigenesis [40]. As an E3 ubiquitin ligase, *TRIM21* regulates multiple substrates through different types of ubiquitination, among which K11, K27, K33, K48 and K63 have been reported [41, 46–49]. In the present study, we found that *UBE2S* transferred Ub to *TRIM21* and cooperatively formed K11-linked ubiquitin chains to mediate the degradation of *LPP* in BCa.

To date, proteasome inhibitors, including bortezomib (Velcade), carfilzomib (Kyprolis), and ixazomib (Ninlaro), have become foundational chemotherapeutic drugs in the clinical treatment of multiple myeloma and mantle cell lymphoma [43]. In the present study, we confirmed that cephalomannine is an effective inhibitor that attenuates *UBE2S* expression and the invasion and lymphatic metastasis of BCa as well as BCa organoids without significant toxicity. Similarly, cephalomannine is reported to inhibit the growth of hepatocellular carcinoma and alleviate the bone metastasis of prostate cancer [20, 21]. Overall, targeting *UBE2S* with cephalomannine is a prospective therapeutic method for treating the progression and lymphatic metastasis of BCa.

In summary, our work provides evidence for the crucial roles of *UBE2S* in the lymphatic metastasis of BCa and clarifies the *UBE2S*-*TRIM21*-*LPP* axis as a novel proteasome-mediated mechanism. Cephalomannine is a promising treatment targeting *UBE2S* and serves as an effective drug for metastatic BCa.

## Supplementary information

Supplemental materials

aj-checklist

Original Data File

## Data Availability

All datasets generated and analyzed during this study are included in this published article and its Supplementary Information files. Additional data are available from the corresponding author upon reasonable request.

## References

[1] Sung H, Ferlay J, Siegel RL, Laversanne M, Soerjomataram I, Jemal A (2021). Global Cancer Statistics 2020: GLOBOCAN Estimates of Incidence and Mortality Worldwide for 36 Cancers in 185 Countries. CA Cancer J Clin.

[2] Liu S, Chen X, Lin T (2022). Emerging strategies for the improvement of chemotherapy in bladder cancer: current knowledge and future perspectives. J Adv Res.

[3] Chen X, Zhang J, Ruan W, Huang M, Wang C, Wang H (2020). Urine DNA methylation assay enables early detection and recurrence monitoring for bladder cancer. J Clin Invest.

[4] Ruan W, Chen X, Huang M, Wang H, Chen J, Liang Z (2021). A urine-based DNA methylation assay to facilitate early detection and risk stratification of bladder cancer. Clin Epigenetics.

[5] Hautmann RE, de Petriconi RC, Pfeiffer C, Volkmer BG (2012). Radical cystectomy for urothelial carcinoma of the bladder without neoadjuvant or adjuvant therapy: long-term results in 1100 patients. Eur Urol.

[6] Liu S, Chen X, Lin T (2021). Lymphatic metastasis of bladder cancer: molecular mechanisms, diagnosis and targeted therapy. Cancer Lett.

[7] Chen Z, Chen X, Xie R, Huang M, Dong W, Han J (2019). DANCR promotes metastasis and proliferation in bladder cancer cells by enhancing IL-11-STAT3 signaling and CCND1 expression. Mol Ther.

[8] Wang C, Liu Q, Huang M, Zhou Q, Zhang X, Zhang J (2020). Loss of GATA6 expression promotes lymphatic metastasis in bladder cancer. FASEB J.

[9] Xie R, Chen X, Cheng L, Huang M, Zhou Q, Zhang J (2021). NONO inhibits lymphatic metastasis of bladder cancer via alternative splicing of SETMAR. Mol Ther.

[10] Zamaraev AV, Kopeina GS, Prokhorova EA, Zhivotovsky B, Lavrik IN (2017). Post-translational modification of caspases: the other side of apoptosis regulation. Trends Cell Biol.

[11] Liu J, Qian C, Cao X (2016). Post-translational modification control of innate immunity. Immunity..

[12] Li W, Li F, Zhang X, Lin HK, Xu C (2021). Insights into the post-translational modification and its emerging role in shaping the tumor microenvironment. Signal Transduct Target Ther.

[13] Dong B, Wu Y (2021). Epigenetic regulation and post-translational modifications of SNAI1 in cancer metastasis. Int J Mol Sci.

[14] Song L, Luo ZQ (2019). Post-translational regulation of ubiquitin signaling. J Cell Biol.

[15] Hershko A, Ciechanover A (1998). The ubiquitin system. Annu Rev Biochem.

[16] Yau R, Rape M (2016). The increasing complexity of the ubiquitin code. Nat Cell Biol.

[17] Swatek KN, Komander D (2016). Ubiquitin modifications. Cell Res.

[18] Cockram PE, Kist M, Prakash S, Chen SH, Wertz IE, Vucic D (2021). Ubiquitination in the regulation of inflammatory cell death and cancer. Cell Death Differ.

[19] Deng L, Meng T, Chen L, Wei W, Wang P (2020). The role of ubiquitination in tumorigenesis and targeted drug discovery. Signal Transduct Target Ther.

[20] Zhang RY, Liu ZK, Wei D, Yong YL, Lin P, Li H (2021). UBE2S interacting with TRIM28 in the nucleus accelerates cell cycle by ubiquitination of p27 to promote hepatocellular carcinoma development. Signal Transduct Target Ther.

[21] Peng S, Chen X, Huang C, Yang C, Situ M, Zhou Q (2022). UBE2S as a novel ubiquitinated regulator of p16 and beta-catenin to promote bone metastasis of prostate cancer. Int J Biol Sci.

[22] Zhou Q, Chen X, He H, Peng S, Zhang Y, Zhang J (2021). WD repeat domain 5 promotes chemoresistance and Programmed Death-Ligand 1 expression in prostate cancer. Theranostics..

[23] Xiao KH, Teng K, Ye YL, Tan L, Chen MK, Liang HT (2019). Kinesin family member C1 accelerates bladder cancer cell proliferation and induces epithelial-mesenchymal transition via Akt/GSK3beta signaling. Cancer Sci.

[24] Chen X, Xie R, Gu P, Huang M, Han J, Dong W (2019). Long noncoding RNA LBCS inhibits self-renewal and chemoresistance of bladder cancer stem cells through epigenetic silencing of SOX2. Clin Cancer Res.

[25] Xie R, Chen X, Chen Z, Huang M, Dong W, Gu P (2019). Polypyrimidine tract binding protein 1 promotes lymphatic metastasis and proliferation of bladder cancer via alternative splicing of MEIS2 and PKM. Cancer Lett.

[26] Zhang J, Zhou Q, Xie K, Cheng L, Peng S, Xie R (2021). Targeting WD repeat domain 5 enhances chemosensitivity and inhibits proliferation and programmed death-ligand 1 expression in bladder cancer. J Exp Clin Cancer Res.

[27] Huang M, Dong W, Xie R, Wu J, Su Q, Li W (2022). HSF1 facilitates the multistep process of lymphatic metastasis in bladder cancer via a novel PRMT5-WDR5-dependent transcriptional program. Cancer Commun.

[28] Xie R, Cheng L, Huang M, Huang L, Chen Z, Zhang Q (2023). NAT10 drives cisplatin chemoresistance by enhancing ac4C-associated DNA repair in bladder cancer. Cancer Res.

[29] Gao W, Li Y, Liu X, Wang S, Mei P, Chen Z (2022). TRIM21 regulates pyroptotic cell death by promoting Gasdermin D oligomerization. Cell Death Differ.

[30] Wang X, Cheng H, Zhao J, Li J, Chen Y, Cui K (2022). Long noncoding RNA DLGAP1-AS2 promotes tumorigenesis and metastasis by regulating the Trim21/ELOA/LHPP axis in colorectal cancer. Mol Cancer.

[31] Chen X, Li Z, Yong H, Wang W, Wang D, Chu S (2021). Trim21-mediated HIF-1alpha degradation attenuates aerobic glycolysis to inhibit renal cancer tumorigenesis and metastasis. Cancer Lett.

[32] Zhang Y, Hou J, Shi S, Du J, Liu Y, Huang P (2021). CSN6 promotes melanoma proliferation and metastasis by controlling the UBR5-mediated ubiquitination and degradation of CDK9. Cell Death Dis.

[33] Song M, Yeku OO, Rafiq S, Purdon T, Dong X, Zhu L (2020). Tumor derived UBR5 promotes ovarian cancer growth and metastasis through inducing immunosuppressive macrophages. Nat Commun.

[34] Xu Z, Chen S, Liu R, Chen H, Xu B, Xu W (2022). Circular RNA circPOLR2A promotes clear cell renal cell carcinoma progression by facilitating the UBE3C-induced ubiquitination of PEBP1 and, thereby, activating the ERK signaling pathway. Mol Cancer.

[35] Hang C, Zhao S, Wang T, Zhang Y (2021). Oncogenic UBE3C promotes breast cancer progression by activating Wnt/beta-catenin signaling. Cancer Cell Int.

[36] Li Z, Wang Y, Li Y, Yin W, Mo L, Qian X (2018). Ube2s stabilizes beta-Catenin through K11-linked polyubiquitination to promote mesendoderm specification and colorectal cancer development. Cell Death Dis.

[37] Zhao J, Cai B, Shao Z, Zhang L, Zheng Y, Ma C (2021). TRIM26 positively regulates the inflammatory immune response through K11-linked ubiquitination of TAB1. Cell Death Differ.

[38] Martinez-Ferriz A, Ferrando A, Fathinajafabadi A, Farras R (2022). Ubiquitin-mediated mechanisms of translational control. Semin Cell Dev Biol.

[39] Oke V, Wahren-Herlenius M (2012). The immunobiology of Ro52 (TRIM21) in autoimmunity: a critical review. J Autoimmun.

[40] Chen X, Cao M, Wang P, Chu S, Li M, Hou P (2022). The emerging roles of TRIM21 in coordinating cancer metabolism, immunity and cancer treatment. Front Immunol.

[41] Xue B, Li H, Guo M, Wang J, Xu Y, Zou X (2018). TRIM21 promotes innate immune response to RNA viral infection through Lys27-linked polyubiquitination of MAVS. J Virol.

[42] Ngan E, Kiepas A, Brown CM, Siegel PM (2018). Emerging roles for LPP in metastatic cancer progression. J Cell Commun Signal.

[43] Narayanan S, Cai CY, Assaraf YG, Guo HQ, Cui Q, Wei L (2020). Targeting the ubiquitin-proteasome pathway to overcome anti-cancer drug resistance. Drug Resist Updat.

[44] Kuriyama S, Yoshida M, Yano S, Aiba N, Kohno T, Minamiya Y (2016). LPP inhibits collective cell migration during lung cancer dissemination. Oncogene..

[45] Colas E, Muinelo-Romay L, Alonso-Alconada L, Llaurado M, Monge M, Barbazan J (2012). ETV5 cooperates with LPP as a sensor of extracellular signals and promotes EMT in endometrial carcinomas. Oncogene..

[46] Zhu X, Xue J, Jiang X, Gong Y, Gao C, Cao T (2022). TRIM21 suppresses CHK1 activation by preferentially targeting CLASPIN for K63-linked ubiquitination. Nucleic Acids Res.

[47] Yuan L, Li P, Jing H, Zheng Q, Xiao H (2022). trim-21 promotes proteasomal degradation of CED-1 for apoptotic cell clearance in C. elegans. Elife..

[48] Fan X, Zhou D, Zhao B, Sha H, Li M, Li X (2021). Rab11-FIP1 and Rab11-FIP5 regulate pIgR/pIgA transcytosis through TRIM21-mediated polyubiquitination. Int J Mol Sci.

[49] Song Y, Wu X, Xu Y, Zhu J, Li J, Zou Z (2020). HPV E7 inhibits cell pyroptosis by promoting TRIM21-mediated degradation and ubiquitination of the IFI16 inflammasome. Int J Biol Sci.

