# Supplementary material for: UBE2S interacting with TRIM21 mediates the K11-linked ubiquitination of LPP to promote the lymphatic metastasis of bladder cancer
Source: Cell Death Dis. 2023 Jul 8;14(7):408. doi: 10.1038/s41419-023-05938-2 (PMC10329682; doi:10.1038/s41419-023-05938-2)
Supplement: Supplementary file 1 — Supplemental materials [file 41419_2023_5938_MOESM1_ESM.docx]

**Supplementary materials for:**

**UBE2S interacting with TRIM21 mediates the K11-linked ubiquitination of LPP to promote the lymphatic metastasis of bladder cancer**

**Running title: UBE2S facilitates lymphatic metastasis of BCa**

Kanghua Xiao^1,2†^, Shengmeng Peng^1,2†^, Junlin Lu^1,2†^, Ting Zhou^3^, Xuwei Hong^4^, Siting Chen^1,2^, Guangyao Liu^5^, Hong Li^6^, Jian Huang^1,2,7*^, Xu Chen^1,2,7*^, Tianxin Lin^1,2,7*^

**Table of contents**

Supplementary figures ………………………………………………………………………………………2-9

Supplementary tables …………………………………………………………………………………… 10-14

Supplementary material and methods …………………………………………………………………… 15-16

**Supplementary figures**


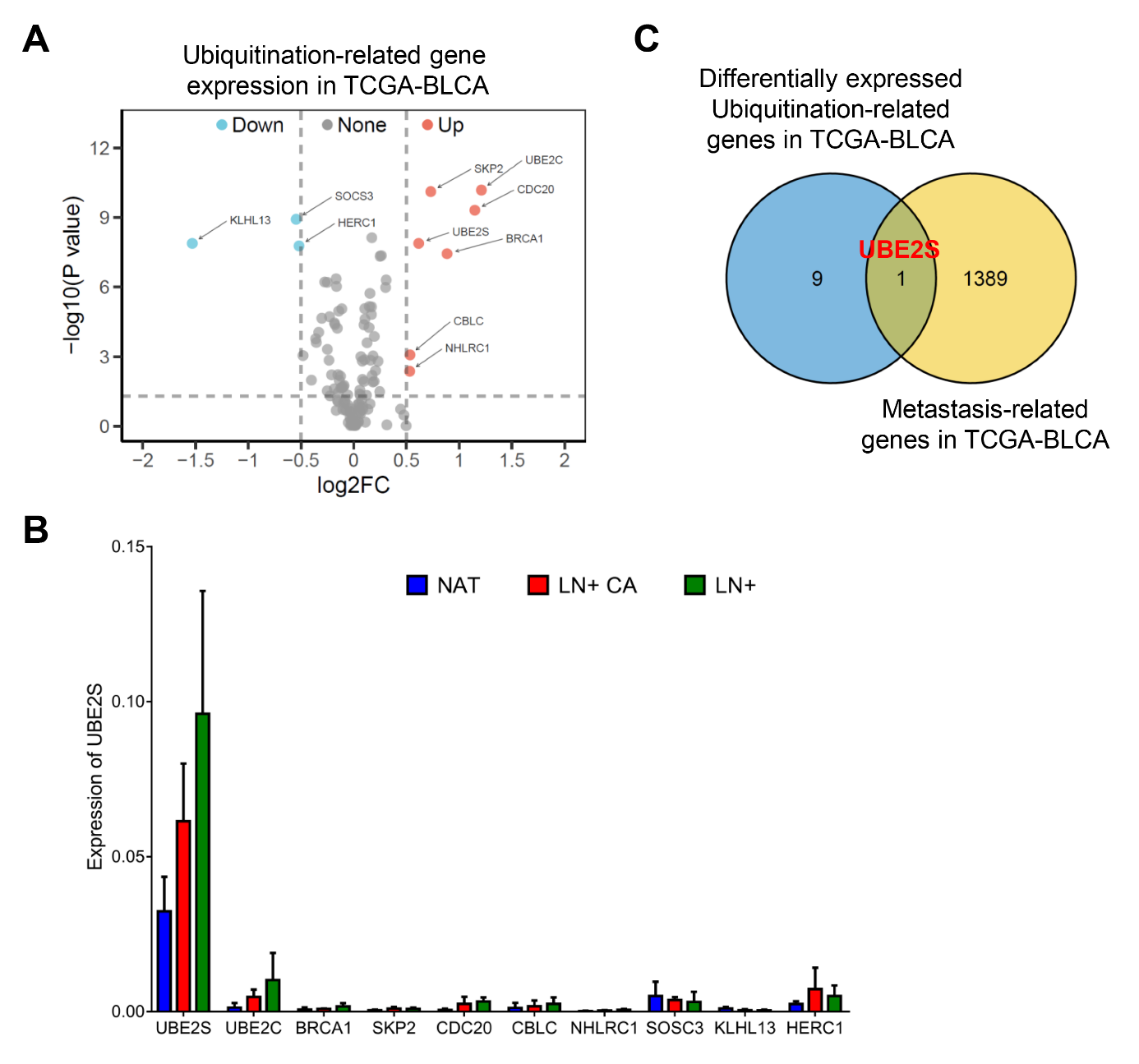


**Figure S1. *UBE2S,* a highly expressed gene, is associated with both ubiquitination and metastasis in BCa. A.** Ten differentially expressed ubiquitination-related genes between BCa and normal tissues in the TCGA-BLCA cohort displayed in a volcano plot. The *P* value was set as < 0.05, and fold change (FC) >0.5. **B.** The expression of ten ubiquitination-related genes detected by qPCR (n=5). **C.** The intersection between ubiquitination- and metastasis-related genes in the TCGA-BLCA database shown in a Venn diagram.


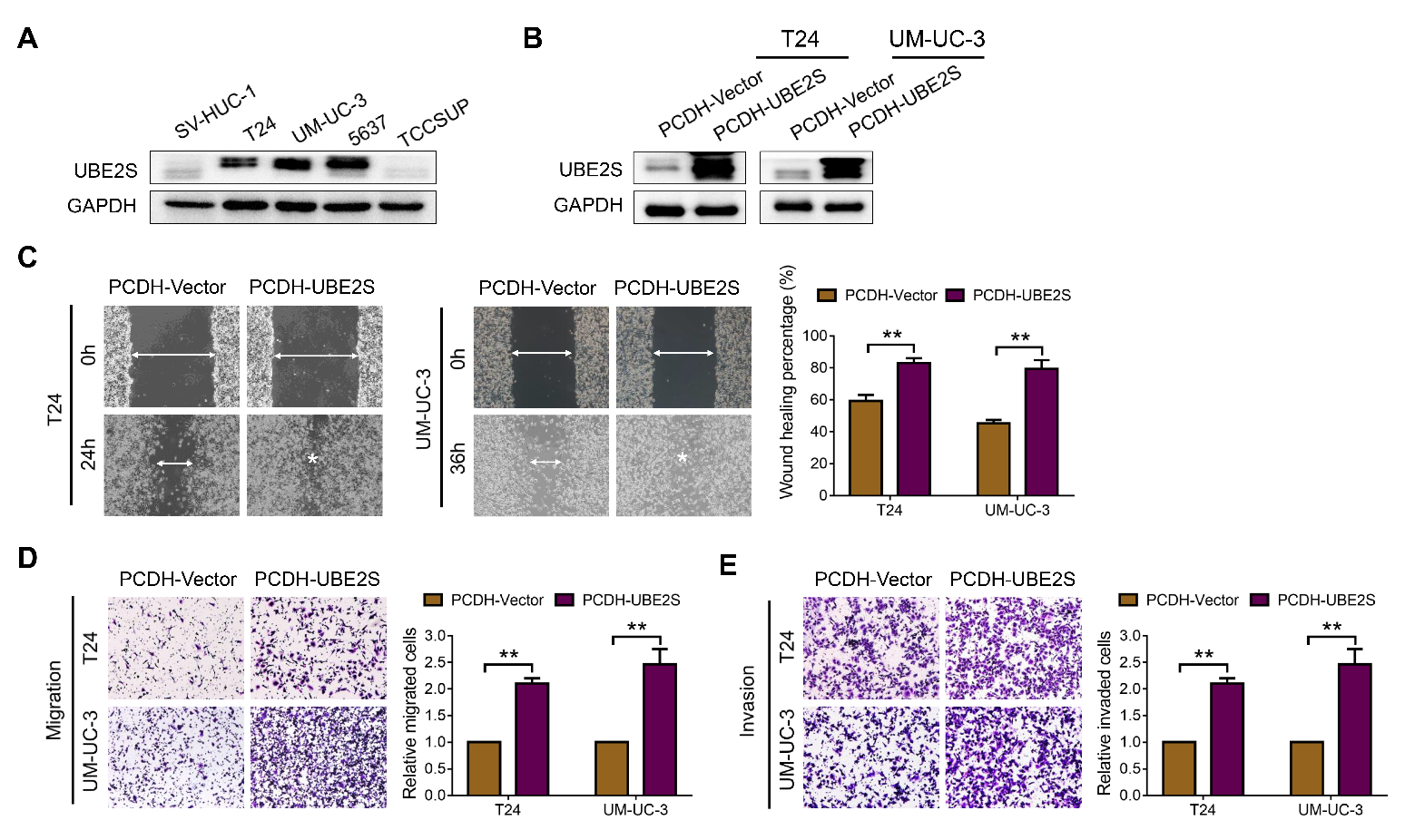


**Figure S2. *UBE2S* promotes the migration and invasion of BCa cells. A.** The protein levels of *UBE2S* expression in human uroepithelial cells (SV-HUC-1) and four BCa cell lines (T24, UM-UC-3, 5637 and TCCSUP). **B.** Western blot analysis of *UBE2S* protein expression levels in *UBE2S*-overexpressing BCa cells. **C-E.** Representative images and quantitative analysis of wound healing (C), transwell migration (D) and invasion (E) assays in *UBE2S*-overexpressing BCa cells. ** *P* < 0.01.


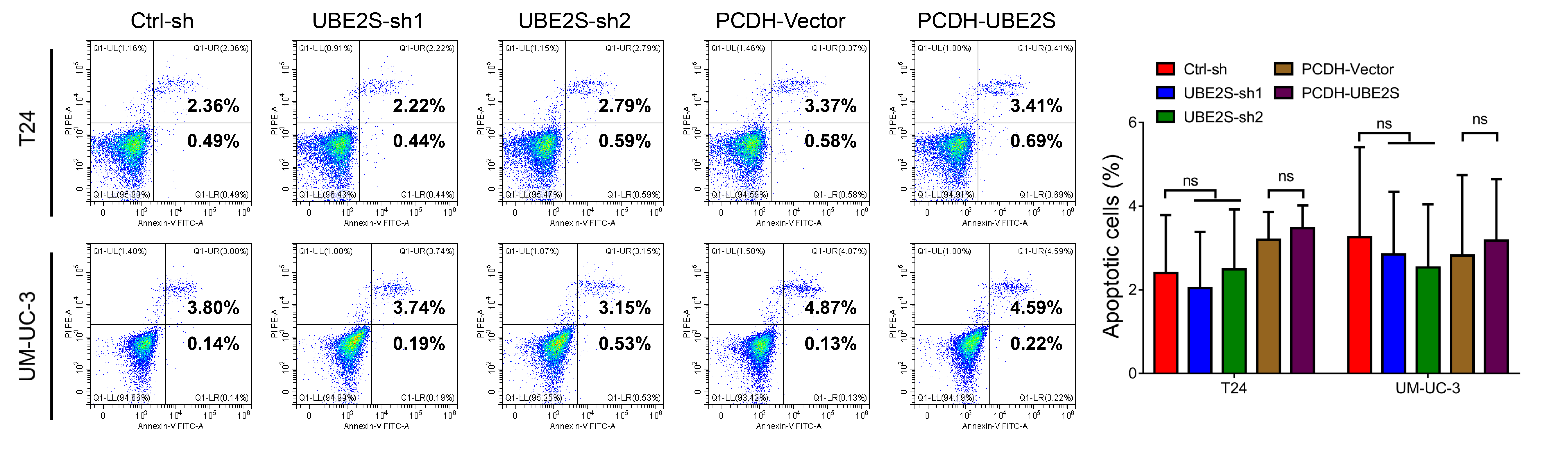


**Figure S3. *UBE2S* does not affect the apoptosis of BCa cells treated for 48 h.** Representative images and histogram analysis of cell apoptosis of BCa cells in different groups. ns, not significant.


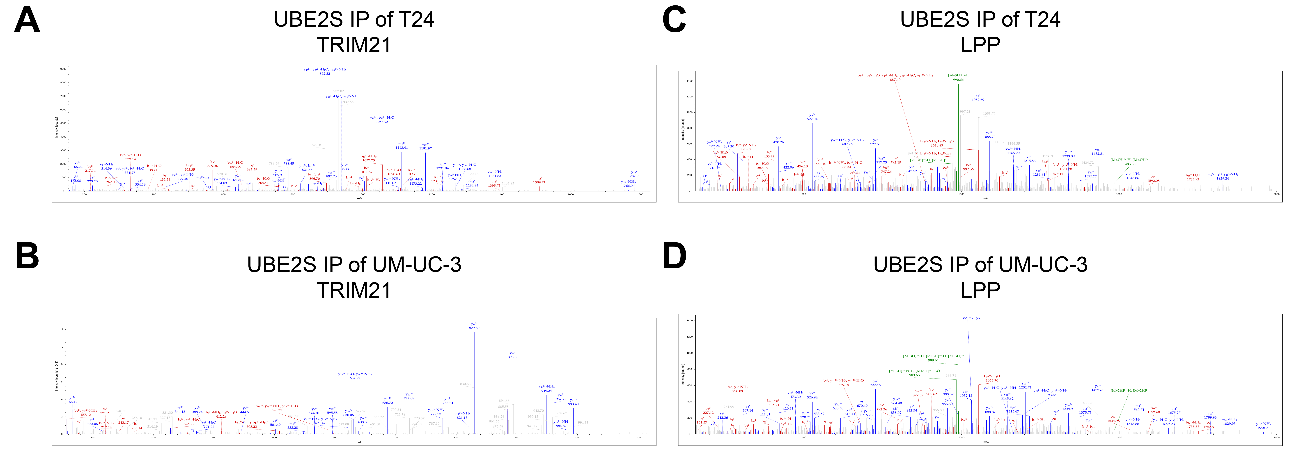


**Figure S4. *UBE2S* interacts with *TRIM21* and *LPP* in BCa cells. A-B.** The representative peptide of *TRIM21* from mass spectrometry of *UBE2S* IP in BCa cells. **C-D.** The representative peptide of *LPP* from mass spectrometry of *UBE2S* IP in BCa cells.


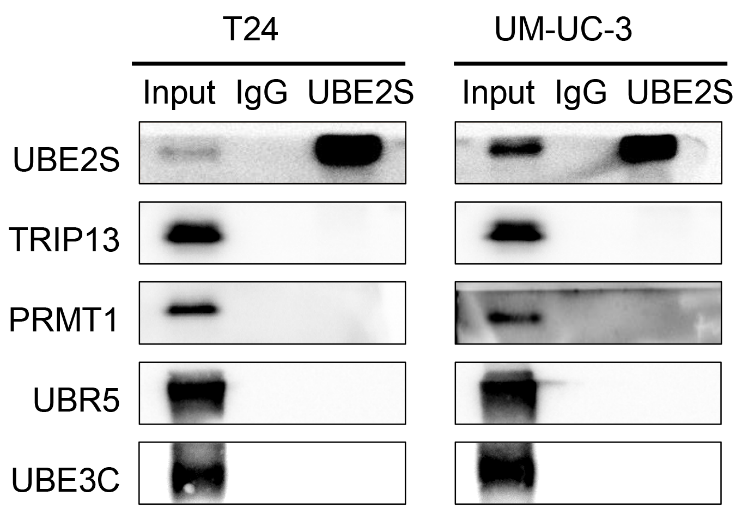


**Figure S5. *UBE2S* does not interact with *TRIP13*, *PRMT1*, *UBR5* and *UBE3C* in BCa cells.** The endogenous interaction among *UBE2S*, *TRIP13*, *PRMT1*, *UBR5* and *UBE3C* in BCa cells was shown by co-IP and western blot assays.


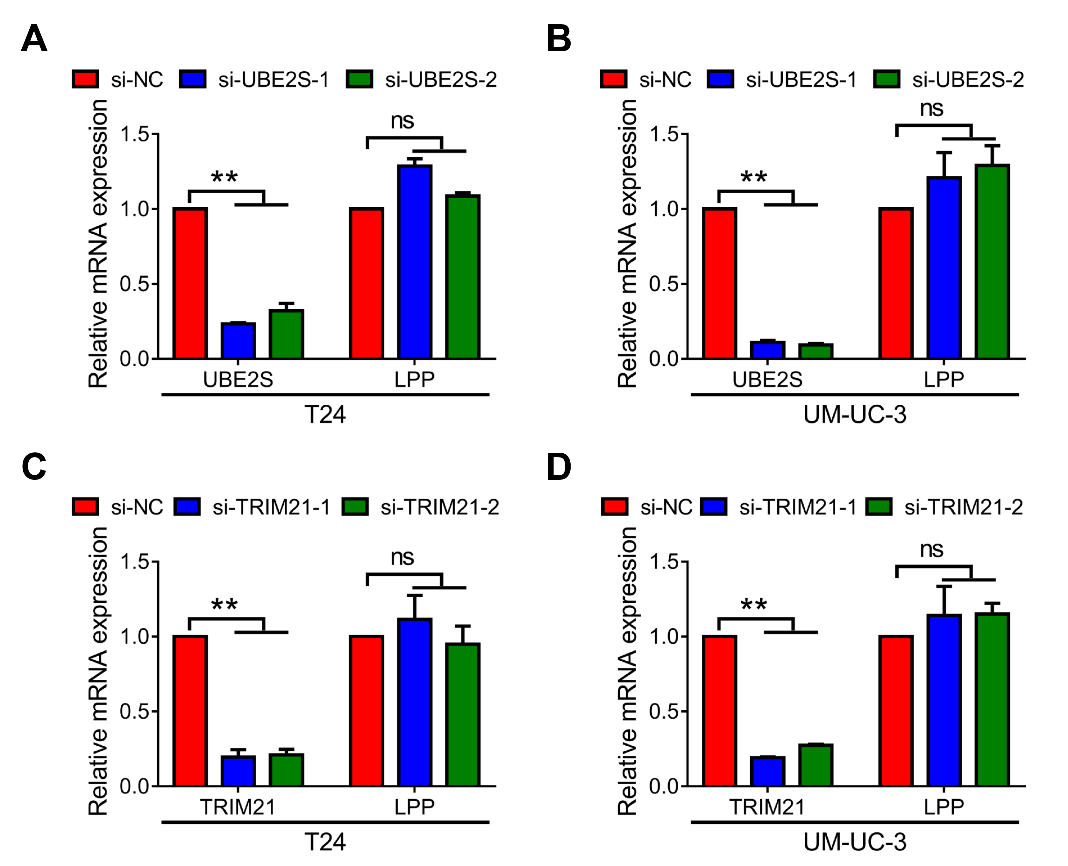


**Figure S6. Neither knockdown of *UBE2S* nor *TRIM21* obviously increased the mRNA levels of *LPP*.** A-D. Quantification of *LPP* mRNA expression by qPCR after *UBE2S* or *TRIM21* knockdown in T24 and UM-UC-3 cells. ** P < 0.01; ns, not significant.


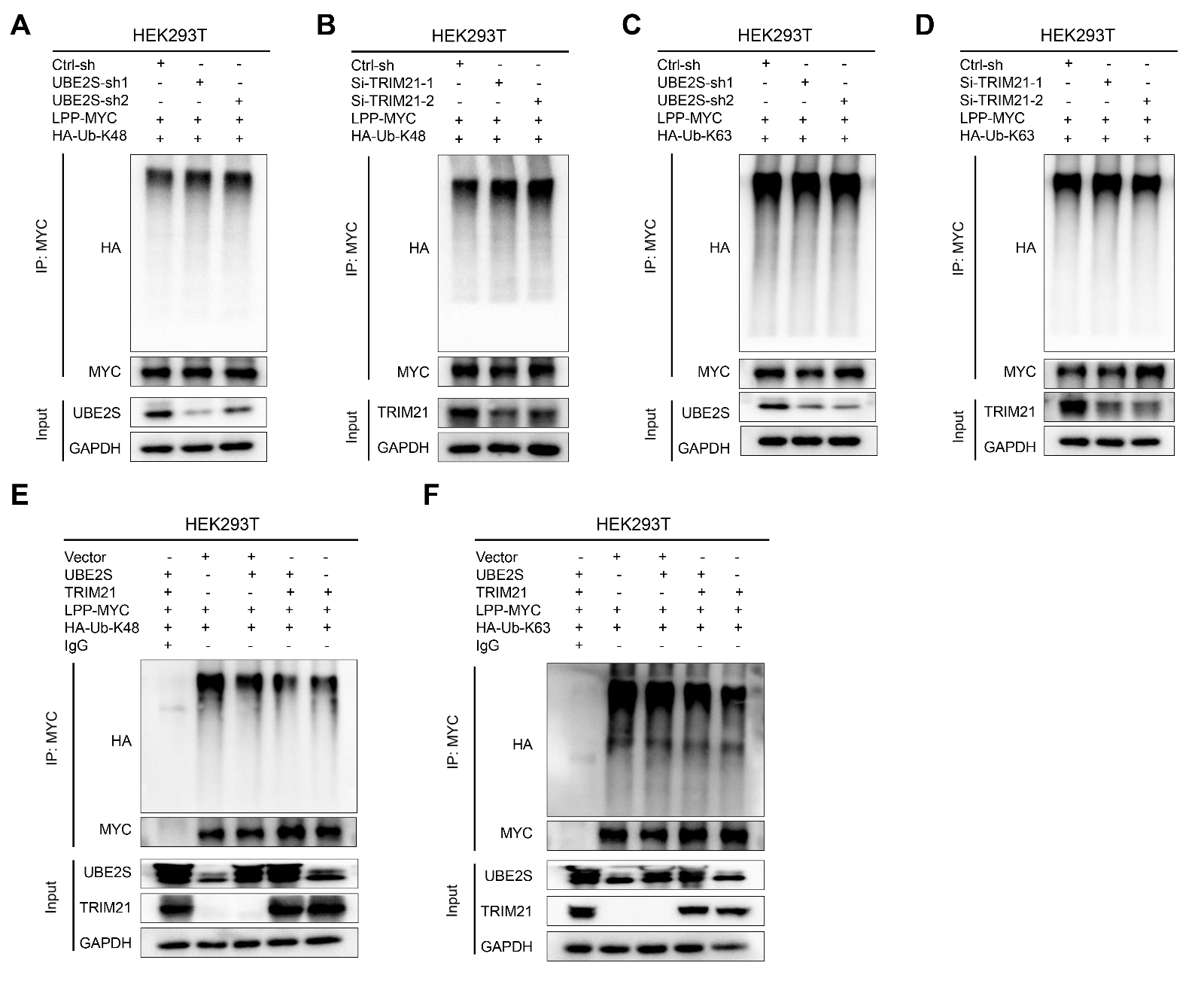


**Figure S7. *UBE2S* degrades *LPP* not through K48-linked or K63-linked ubiquitination. A-D.** K48-linked and K63-linked ubiquitination levels of *LPP* in *UBE2S*-knockdown or *TRIM21*-knockdown HEK293T cells. **E-F.** K48-linked and K63-linked ubiquitination levels of *LPP* in *UBE2S*- and *TRIM21*-overexpressing HEK293T cells.


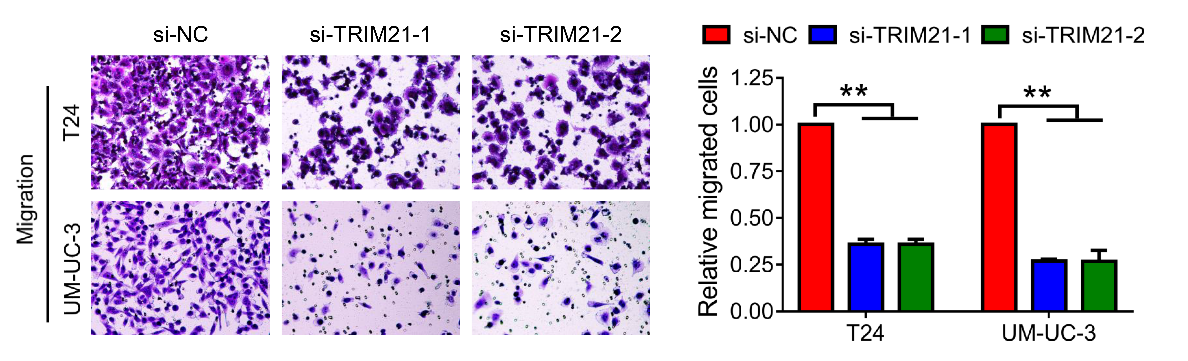


**Figure S8. *TRIM21* knockdown inhibits BCa cell migration.** Representative images and quantitative analysis of transwell migration assays in *TRIM21*-silenced BCa cells. ** *P* < 0.01.


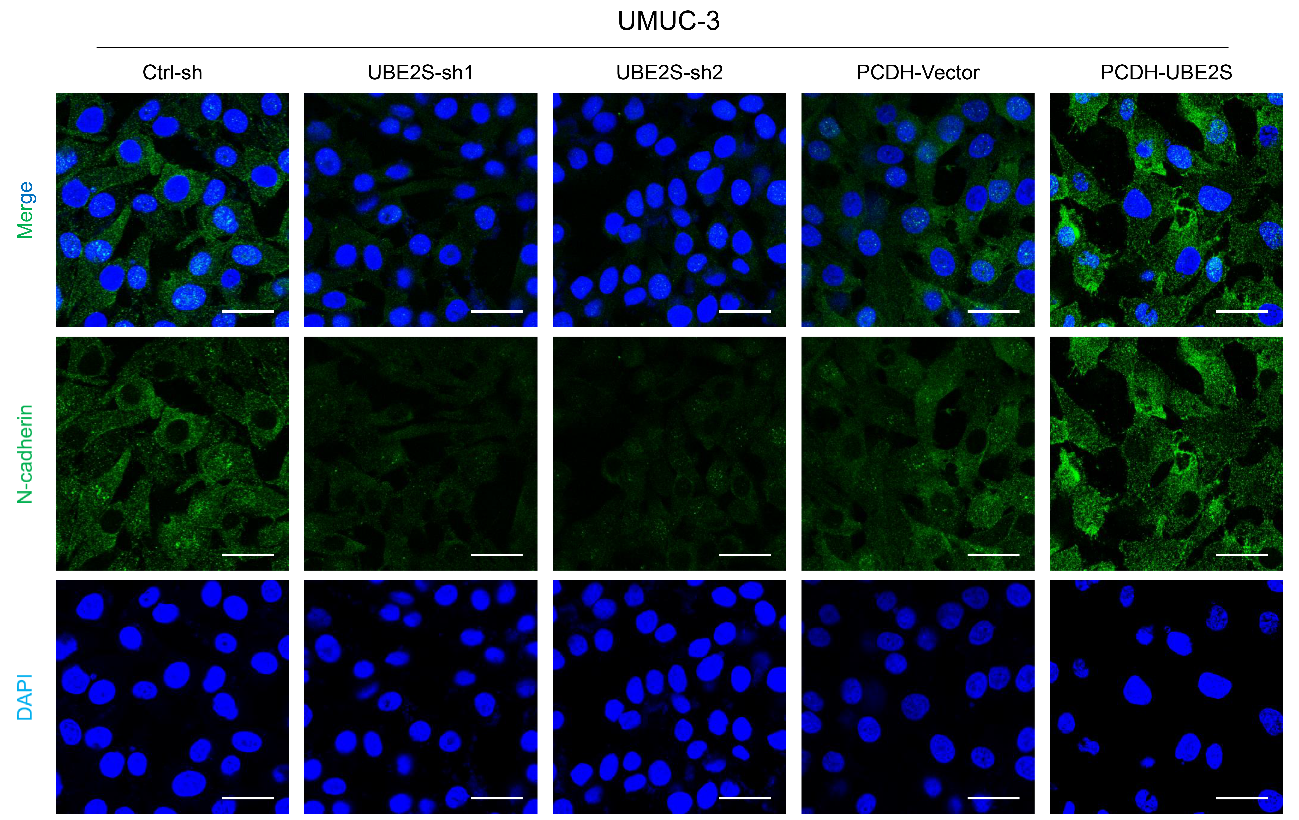


**Figure S9. *UBE2S* increases *N-cadherin* expression in BCa cells.** Representative immunofluorescence images showing *N-cadherin* expression in *UBE2S*-silenced and UBE2S-overexpressing UM-UC-3 cells. Scale bars: white, 20 μm**.**


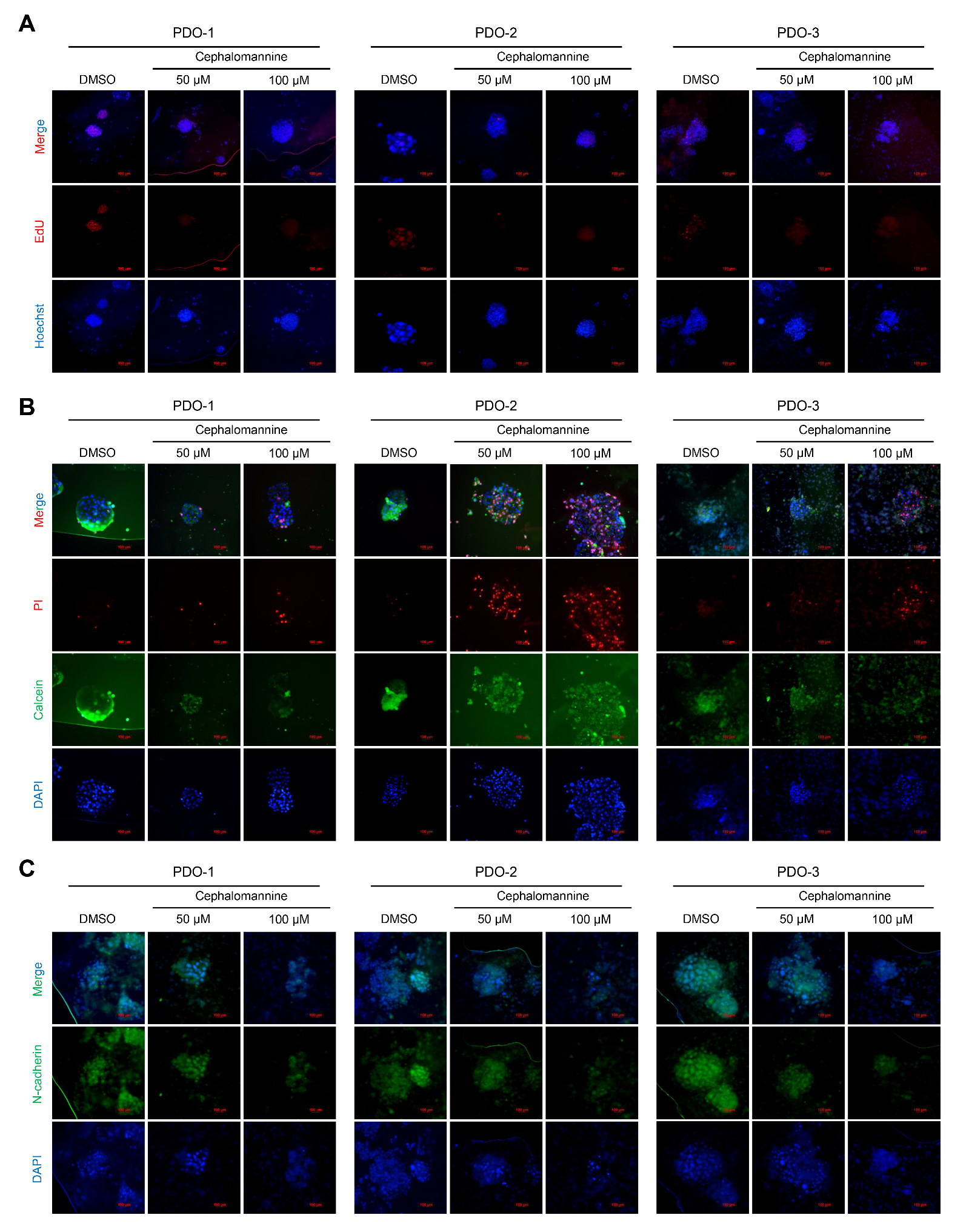


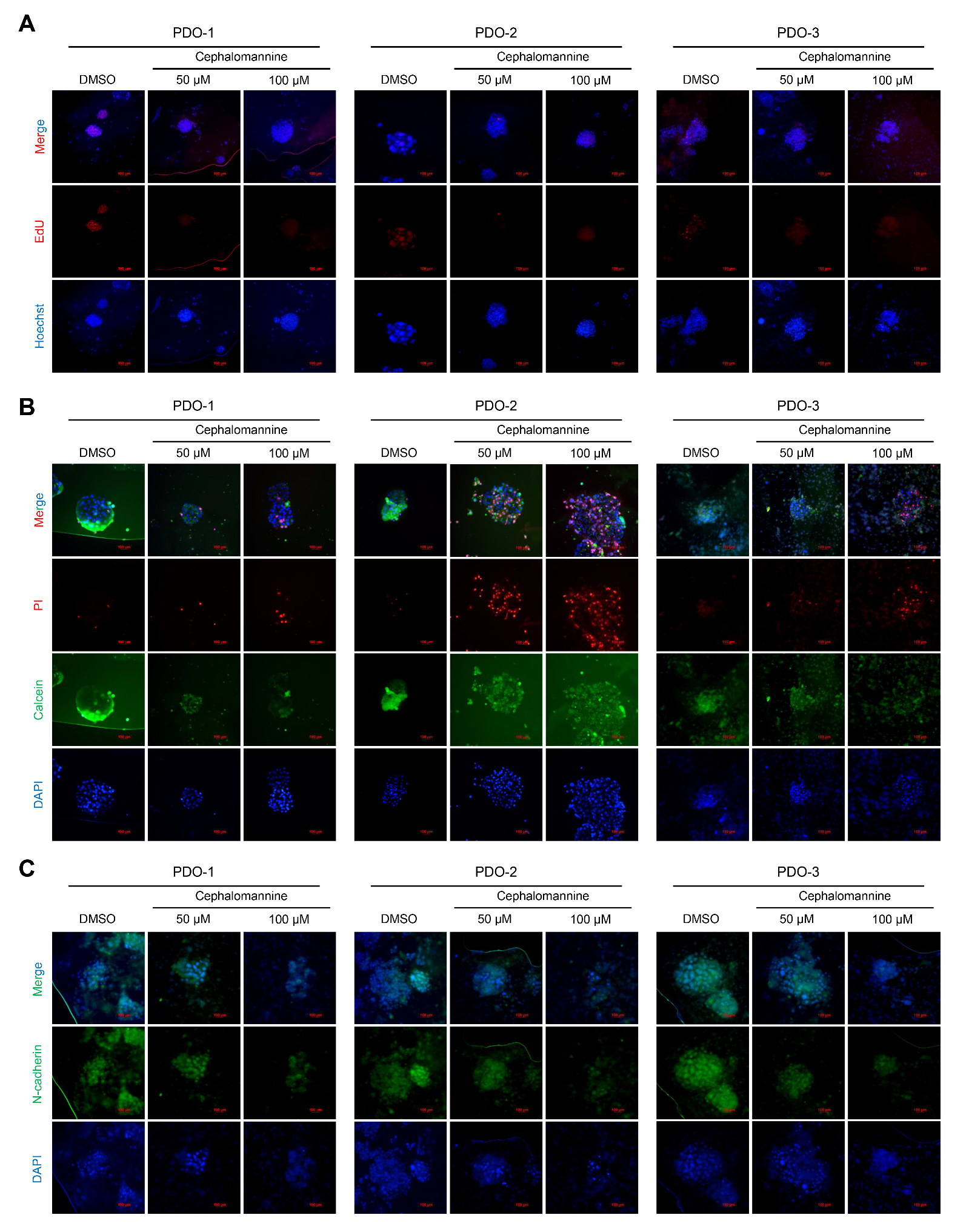


**Figure S10. Cephalomannine inhibited the proliferation, viability and *N-cadherin* expression of BCa organoids in a dose-dependent manner. A-C.** Representative images showing proliferation (A), viability (B) and *N-cadherin* (C) expression of the other three BCa organoids treated with Cephalomannine at the indicated concentrations. PDO, patient-derived organoid.

**Supplementary tables**

**Table S1. Clinicopathologic characteristics of BCa patients in cohort 1 and cohort 2.**

| **Variables** | | **Cohort 1** | **Cohort 2** |
| --- | --- | --- | --- |
|  |  | **Number of cases (%)** | **Number of cases (%)** |
| Total | | 210 | 59 |
| Age | <60 | 97(46.2) | 27 (45.8) |
|  | ≥60 | 113(53.8) | 32 (54.2) |
| Gender | Female | 29(13.8) | 11 (18.6) |
|  | Male | 181(86.2) | 48 (81.4) |
| Histological grade | Low | 26(12.4) | 12 (20.3) |
|  | High | 184(87.6) | 47 (79.7) |
| T stage | pTa-T1 | 27(12.9) | 12(20.3) |
|  | pT2 | 79(37.6) | 19(32.2) |
|  | pT3 | 56(26.7) | 16(27.1) |
|  | pT4 | 48(22.9) | 12(20.3) |
| N stage | Negative | 153(72.9) | 42 (71.2) |
|  | Positive | 57(27.1) | 17 (28.8) |
| M stage | Negative | 200(95.2) | 54 (91.5) |
|  | Positive | 10(4.8) | 5 (8.5) |

**Table S2. Antibodies used in this study.**

| **Protein** | **Manufacturer** | **Cat number** | **Application** |
| --- | --- | --- | --- |
| *UBE2S* | Proteintech | 14115-1-AP | 1:1000 for IHC and WB; 2 µg for IP; 1:200 for IF |
| *LPP* | Proteintech | 25045-1-AP | 1:1000 for IHC and WB; 2 µg for IP; 1:200 for IF |
| *TRIM21* | Proteintech | 12108-1-AP | 1:1000 for WB; 2 µg for IP; 1:200 for IF |
| *TRIP13* | Proteintech | 19602-1-AP | 1:1000 for WB |
| *PRMT1* | Zen BioScience | 385446 | 1:1000 for WB |
| *UBR5* | Proteintech | 22782-1-AP | 1:1000 for WB |
| *UBE3C* | Abcam | ab226173 | 1:1000 for WB |
| *N-Cadherin* | Proteintech | 22018-1-AP | 1:1000 for IHC and WB; 1:200 for IF |
| *Vimentin* | Proteintech | 10366-1-AP | 1:3000 for IHC; 1:2000 for WB |
| *E-Cadherin* | Proteintech | 20874-1-AP | 1:1000 for IHC and WB |
| MYC tag | Beijing Ray Antibody Biotech | RM1003 | 1:1000 for WB; 2 µg for IP |
| HA tag | Beijing Ray Antibody Biotech | RM1004 | 1:1000 for WB |
| IgG | CST | #3900 | 2 µg for IP |

IHC: Immunohistochemistry; WB: Western blotting; IP: Immunoprecipitation; IF: Immunofluorescence; Proteintech (Wuhan, Hubei, China); Zen BioScience (Chengdu, Chongqing, China); Abcam (Cambridge, UK); Beijing Ray Antibody Biotech (Beijing, China); CST (Danvers, Massachusetts, USA).

**Table S3. Sequences of siRNA oligos and shRNAs used in this study.**

| **Name** | **Sequence 5’-3’** |
| --- | --- |
| **siRNA** |  |
| si-Ctrl | UUCUCCGAACGUGUCACGUTT |
| si-*UBE2S*-1 | ACAUCAUCCGCCUGGUGUATT |
| si-*UBE2S*-2 | CAUAUGCUGGAGGUCUGUUTT |
| si-*LPP*-1 | CCCTTCCATCTATCTCTGGAA |
| si-*LPP*-2 | CAGCCATTCTATGCTGTGGAA |
| si-*TRIM21*-1 | GCAGAGCAUACCUGGAAAUTT |
| si-*TRIM21*-2 | CCUGUUCUGUGAGAAAGAUTT |
| **shRNA** |  |
| sh-Ctrl | CAACAAGATGAAGAGCACCAA |
| *UBE2S*-sh1 | ACATCATCCGCCTGGTGTA |
| *UBE2S*-sh2 | CATATGCTGGAGGTCTGTT |

**Table S4. Primers for qPCR used in this study.**

| **Primer Name** | | **Sequence 5’-3’** |
| --- | --- | --- |
| *UBE2S* | Forward | ACAAGGAGGTGACGACACTGA |
|  | Reverse | CCACGTTCGGGTGGAAGAT |
| *LPP* | Forward | GTTTCACCTGCGTGATGTGCCA |
|  | Reverse | GGCTGGCATAATAGGCTCCTTG |
| *TRIM21* | Forward | CAGAACTCAGGAGTGTGTGCCA |
|  | Reverse | TCCAAGCCTCACTTGTCTCCGA |
| *GAPDH* | Forward | CAAGGCTGAGAACGGGAAG |
|  | Reverse | TGAAGACGCCAGTGGACTC |
| *N-cadherin* | Forward | CCTCCAGAGTTTACTGCCATGAC |
|  | Reverse | GTAGGATCTCCGCCACTGATTC |
| *Vimentin* | Forward | AGGCAAAGCAGGAGTCCACTGA |
|  | Reverse | ATCTGGCGTTCCAGGGACTCAT |
| *E-cadherin* | Forward | ACGCATTGCCACATACACTC |
|  | Reverse | GGTGTTCACATCATCGTCCG |

**Table S5. Clinical information of human BCa organoids.**

| **ID** | **Source** | **Age** | **Gender** | **Histological grade** | **T stage** | **Prior therapy** |
| --- | --- | --- | --- | --- | --- | --- |
| PDO-1 | RC | 63 | Male | High grade | T3 | None |
| PDO-2 | RC | 59 | Male | High grade | T3 | GC+ Camrelizumab |
| PDO-3 | TURBT | 45 | Male | High grade | T1 | BCG+Epirubicin |
| PDO-4 | TURBT | 56 | Male | High grade | T1 | None |

PDO, patient-derived organoid; RC: radical cystectomy; TURBT: transurethral resection of bladder tumor; GC: Gemcitabine and Cisplatin.

**Table S6. Contents of BCa organoids culture medium.**

| **Reagent** | **Supplier** | **Cat. No.** | **Final concentration** |
| --- | --- | --- | --- |
| Advanced D-MEM/F-12 | ThermoFisher | 12634010 | 1× |
| Primocin | InvivoGen | ant-pm-05 | 100 µg/mL |
| GlutaMAX | GIBCO | 35050061 | 1× |
| HEPES | GIBCO | 15630080 | 1× |
| B-27^TM^ Supplement | GIBCO | 17504044 | 1× |
| N-Acetylcysteine | Sigma | A9165 | 1.25 mM |
| Nicotinamide | Sigma | N0636 | 10 mM |
| A83-01 | Sigma | SML0788 | 500 nM |
| R-spondin1 | NovoProtein | CX83 | 500 ng/mL |
| Noggin | NovoProtein | CB89 | 100 ng/mL |
| FGF-10 | Peprotech | 100-26 | 20 ng/mL |
| FGF-2 | NovoProtein | C779 | 5 ng/mL |
| EGF | Peprotech | 100-15 | 50 ng/mL |

**Table S7. Correlations between *UBE2S* expression and clinicopathological characteristics in cohort 1.**

| **Characteristics** | **All** | ***UBE2S* expression (%)** | | ***P* value^a^** |
| --- | --- | --- | --- | --- |
|  |  | **Low** | **High** |  |
| Total | 210 | 102 | 108 |  |
| Age(years) |  |  |  | 0.348 |
| <60 | 97 | 51 (50.0) | 46 (42.6) |  |
| ≥60 | 113 | 51 (50.0) | 62 (57.4) |  |
| Gender |  |  |  | 0.066 |
| Female | 29 | 9 (8.8) | 20 (18.5) |  |
| Male | 181 | 93 (91.2) | 88 (81.5) |  |
| Histological grade |  |  |  | **0.004** |
| Low | 26 | 20 (19.6) | 6 (5.6) |  |
| High | 184 | 82 (80.4) | 102 (94.4) |  |
| T stage |  |  |  | **0.008** |
| pTa-T1 | 27 | 20 (19.6) | 7 (6.5) |  |
| pT2-T4 | 183 | 82 (80.4) | 101 (93.5) |  |
| N stage |  |  |  | **<0.001** |
| Negative | 153 | 96 (94.1) | 57 (52.8) |  |
| Positive | 57 | 6 (5.9) | 51 (47.2) |  |
| M stage |  |  |  | **0.030** |
| Negative | 200 | 101 (99.0) | 99 (91.7) |  |
| Positive | 10 | 1 (1.0) | 9 (8.3) |  |

^a^Chi-square test; Bold values represent *P* < 0.05.

**Table S8. Univariate and multivariate analyses of prognostic parameters associated with survival in cohort 1.**

| **Prognostic parameters** | **Univariate** | |  | **Multivariate** | |
| --- | --- | --- | --- | --- | --- |
|  | **HR (95% CI)** | ***P* value** |  | **HR (95% CI)** | ***P* value** |
| **Overall survival (OS)**  Age (years) (≥60/＜60) | 1.980(1.230-3.170) | **0.005** |  | 2.000(1.230-3.250) | **0.005** |
| Gender (Male/Female) | 0.960(0.490-1.860) | 0.896 |  |  |  |
| Histological grade (High/Low) | 0.950(0.500-1.800) | 0.879 |  |  |  |
| T stage (pT2-T4/Ta-T1) | 2.050(0.890-4.720) | 0.092 |  |  |  |
| N stage (Positive/Negative) | 1.990(1.220-3.240) | **0.006** |  | 1.110 (0.630-1.960) | 0.708 |
| M stage (Positive/Negative) | 3.890(1.770-8.540) | **0.001** |  | 3.020 (1.280-7.120) | **0.011** |
| *UBE2S* (High/Low) | 4.240(2.560-7.020) | **<0.001** |  | 3.710 (2.170-6.330) | **<0.001** |
|  |  |  |  |  |  |
| **Disease-free survival (DFS)** |  |  |  |  |  |
| Age (years) (≥60/＜60) | 1.820(1.170-2.830) | **0.007** |  | 1.870(1.190-2.930) | **0.007** |
| Gender (Male/Female) | 1.130(0.580-2.190) | 0.715 |  |  |  |
| Histological grade (High/Low) | 1.080(0.580-2.040) | 0.803 |  |  |  |
| T stage (pT2-T4/Ta-T1) | 2.410(1.050-5.530) | **0.038** |  | 1.770 (0.760-4.140) | 0.185 |
| N stage (Positive/Negative) | 2.590(1.640-4.080) | **<0.001** |  | 1.470 (0.870-2.480) | 0.154 |
| M stage (Positive/Negative) | 3.400(1.550-7.440) | **0.002** |  | 2.140 (0.930-4.920) | **0.072** |
| *UBE2S* (High/Low) | 4.030(2.520-6.440) | **<0.001** |  | 3.090 (1.840-5.180) | **<0.001** |

Bold values represent *P* < 0.05.

**Table S9. List of the top 10** **candidates of *UBE2S*-interacting proteins identified by co-IP and MS.**

| **Gene Symbol** | **Official Full Name** | **Accession** | **Coverage (%)** | **Unique Peptides** | **MW (kDa)** | **Score Sequest HT** |
| --- | --- | --- | --- | --- | --- | --- |
| **E3 enzymes** |  |  |  |  |  |  |
| *TRIM21* | tripartite motif containing 21 | P19474 | 31 | 12 | 54 | 49 |
| *RANBP2* | RAN binding protein 2 | P49792 | 14 | 23 | 358 | 85 |
| *ZNF598* | zinc finger protein 598, E3 ubiquitin ligase | Q86UK7 | 9 | 5 | 99 | 14 |
| *UBR4* | ubiquitin protein ligase E3 component n-recognin 4 | Q5T4S7 | 8 | 30 | 574 | 88 |
| *ITCH* | itchy E3 ubiquitin protein ligase | Q96J02 | 8 | 5 | 103 | 13 |
| *RNF213* | ring finger protein 213 | Q63HN8 | 7 | 30 | 591 | 83 |
| *UFL1* | UFM1 specific ligase 1 | O94874 | 7 | 5 | 90 | 12 |
| *UBE3C* | ubiquitin protein ligase E3C | Q15386 | 6 | 5 | 124 | 14 |
| *UBR5* | ubiquitin protein ligase E3 component n-recognin 5 | O95071 | 4 | 7 | 309 | 23 |
| *HERC2* | HECT and RLD domain containing E3 ubiquitin protein ligase 2 | O95714 | 2 | 7 | 527 | 19 |
|  |  |  |  |  |  |  |
| **Substrates** |  |  |  |  |  |  |
| *NCOA5* | Nuclear receptor coactivator 5 | Q9HCD5 | 25 | 8 | 66 | 31 |
| *GOLGA2* | Golgin subfamily A member 2 | Q08379 | 20 | 19 | 113 | 60 |
| *TRIP13* | thyroid hormone receptor interactor 13 | Q15645 | 20 | 9 | 49 | 23 |
| *NANS* | Sialic acid synthase | Q9NR45 | 19 | 5 | 40 | 15 |
| *ATIC* | Bifunctional purine biosynthesis protein ATIC | P31939 | 17 | 8 | 65 | 26 |
| *LPP* | Lipoma-preferred partner | Q93052 | 15 | 4 | 66 | 11 |
| *COPB2* | COPI coat complex subunit beta 2 | P35606 | 13 | 9 | 102 | 29 |
| *GFPT1* | glutamine--fructose-6-phosphate transaminase 1 | Q06210 | 13 | 6 | 79 | 18 |
| *PRMT1* | Protein arginine N-methyltransferase 1 | Q99873 | 13 | 4 | 42 | 15 |
| *NAA15* | N-alpha-acetyltransferase 15 | Q9BXJ9 | 12 | 10 | 101 | 34 |

MW: Molecular Weight.

**Table S10. Correlations between *LPP* expression and clinicopathological characteristics in cohort 2.**

| **Variables** | **All** | ***LPP* expression (%)** | | | ***P* value^a^** | |
| --- | --- | --- | --- | --- | --- | --- |
|  |  | **Low** | **High** | |  | |
| Total | 59 | 28 | 31 | |  | |
| Age(years) |  |  |  | | 0.377 | |
| <60 | 27 | 15 (53.6) | 12 (38.7) | |  | |
| ≥60 | 32 | 13 (46.4) | 19 (61.3) | |  | |
| Gender |  |  |  | | 0.127 | |
| Female | 11 | 8 (28.6) | 3 (9.7) | |  | |
| Male | 48 | 20 (71.4) | 28 (90.3) | |  | |
| Histological grade |  |  |  | | 0.155 | |
| Low | 12 | 3 (10.7) | 9 (29.0) |  | |  |
| High | 47 | 25 (89.3) | 22 (71.0) | |  | |
| T stage |  |  |  | | 0.439 | |
| pTa-T1 | 12 | 4 (14.3) | 8 (25.8) | |  | |
| pT2-T4 | 47 | 24 (85.7) | 23 (74.2) | |  | |
| N stage |  |  |  | | **<0.001** | |
| Negative | 42 | 13 (46.4) | 29 (93.5) | |  | |
| Positive | 17 | 15 (53.6) | 2 (6.5) | |  | |
| M stage |  |  |  | | 0.291 | |
| Negative | 54 | 24 (85.7) | 30 (96.8) | |  | |
| Positive | 1 | 4 (14.3) | 1 (3.2) | |  | |

^a^Chi-square test; Bold values represent *P* < 0.05.

**Table S11. Univariate and multivariate analyses of prognostic parameters associated with survival in cohort 2.**

| **Prognostic parameters** | **Univariate** | |  | **Multivariate** | |
| --- | --- | --- | --- | --- | --- |
|  | **HR (95% CI)** | ***P* value** |  | **HR (95% CI)** | ***P* value** |
| **Overall survival (OS)** |  |  |  |  |  |
| Age (years) (≥60/＜60) | 1.720(0.760-3.890) | 0.196 |  |  |  |
| Gender (Male/Female) | 0.780(0.290-2.110) | 0.629 |  |  |  |
| Histological grade (High/Low) | 0.780(0.330-1.870) | 0.580 |  |  |  |
| T stage (pT2-T4/Ta-T1) | 1.600(0.550-4.670) | 0.388 |  |  |  |
| N stage (Positive/Negative) | 1.770(0.750-4.150) | 0.191 |  |  |  |
| M stage (Positive/Negative) | 3.670(1.050-12.820) | **0.041** |  | 2.020 (0.570-7.130) | 0.273 |
| *LPP* (High/Low) | 0.190(0.080-0.470) | **<0.001** |  | 0.210 (0.080-0.510) | **0.001** |
|  |  |  |  |  |  |
| **Disease-free survival (DFS)** |  |  |  |  |  |
| Age (years) (≥60/＜60) | 1.810(0.830-3.920) | 0.136 |  |  |  |
| Gender (Male/Female) | 0.990(0.370-2.620) | 0.980 |  |  |  |
| Histological grade (High/Low) | 0.870(0.370-2.040) | 0.744 |  |  |  |
| T stage (pT2-T4/Ta-T1) | 1.950(0.670-5.620) | 0.218 |  |  |  |
| N stage (Positive/Negative) | 2.330(1.050-5.190) | **0.038** |  | 0.960(0.400-2.290) | 0.926 |
| M stage (Positive/Negative) | 2.730(0.790-9.450) | 0.114 |  |  |  |
| *LPP* (High/Low) | 0.170(0.070-0.410) | **<0.001** |  | 0.170 (0.070-0.430) | **<0.001** |

Bold values represent *P* < 0.05.

**Table S12. Blood analysis of mice treated with cephalomannie in BCa.**

| **Blood Index** | **PBS (Mean±SD)** | **Cephalomannie 10 mg/kg (Mean±SD)** | **Cephalomannie 20 mg/kg**  **(Mean±SD)** | **reference value** |
| --- | --- | --- | --- | --- |
| RBC, 10^^12^/L | 6.60±1.80 | 9.91±0.40 | 9.33±0.43 | 6.36-9.42 |
| WBC, 10^^9^/L | 5.10±2.71 | 8.22±2.28 | 5.28±0.23 | 0.80-6.80 |
| Granulocyte% | 14.62±5.60 | 17.76±11.45 | 12.36±3.12 | 8.60-38.90 |
| PLT, 10^^9^/L | 536.60±308.11 | 915.00±231.70 | 833.20±239.27 | 450.00-1590.00 |
| HGB, g/L | 106.40±21.93 | 153.60±5.24 | 149.80±3.49 | 110.00-143.00 |
| ALT, U/L | 294.39±1.15 | 104.82±0.66 | 99.51±0.44 | 10.06-96.47 |
| AST, U/L | 457.10±0.75 | 174.98±3.61 | 265.98±0.83 | 36.31-235.48 |
| ALB, g/L | 40.87±0.17 | 39.03±0.06 | 35.64±0.10 | 21.22-39.15 |
| CREA, umol/l | 29.66±0.18 | 41.21±0.09 | 51.67±0.13 | 10.91-85.09 |

RBC=red blood cell count; WBC= white blood cell count; PLT=platelet count; HGB= hemoglobin; ALT= alanine transaminase; AST= aspartate aminotransferase; ALB= albumin; CREA= creatinine.

**Supplementary material and methods**

**Cell lines and cell culture**

Specifically, SV-HUC-1 cells were cultured in Ham's F-12K medium (PM150910, Procell, China), T24 and 5637 cells were cultured in RPMI 1640 medium, and HEK-293T, UM-UC-3 and TCCSUP cells were cultured in DMEM. All cells were cultured in media supplemented with 10% fetal bovine serum (F7524, Sigma‒Aldrich) and 1% penicillin/streptomycin and grown in a humidified atmosphere with 5% CO_2_ at 37 °C. All cell lines mentioned above had undergone short tandem repeat authentication by IGE Biotechnology (Guangzhou, China) and were confirmed to be negative for mycoplasma contamination.

**Transwell migration, invasion and** **wound healing assays**

For the transwell migration assay, 6 × 10^4^ BCa cells were inoculated into the upper chamber (353097, Falcon), subsequently fixed and stained at the indicated time (usually 8 hours for T24 cells, 21 hours for UM-UC-3 cells). For the transwell invasion assay, the upper chambers were first coated with 25 μg Matrigel (Corning, USA) and then underwent procedures similar to those described above. Because of the presence of Matrigel, cells should be permitted to penetrate for 2 more hours. For the wound healing assay, BCa cells were seeded in six-well plates until they reached confluence, scratched with a 200 μl pipette tip, and then cultured in serum-free medium.

**I****mmunofluorescence staining**

Briefly, BCa cells were seeded in confocal dishes, fixed with 4% paraformaldehyde, blocked with 3% BSA, and incubated with corresponding primary antibodies overnight at 4 °C. The primary antibodies, including antibodies against *UBE2S*, *TRIM21*, *LPP* and *N-cadherin*, are listed in Table S2. After washing with PBS, the cells were incubated with secondary antibodies (SA00013-2, SA00013-3, Proteintech) at room temperature for 1 hour and counterstained with DAPI (Solarbio, China) at room temperature for 5 minutes. For the colocalization of *UBE2S*, *TRIM21* and *LPP*, a multiple fluorescent immunohistochemical kit (10001100020, PANOVUE) was used according to the manufacturer’s instructions. The results were captured using a confocal microscope (Leica, Germany).

**Cell apoptosis, EdU and cell death detection assays**

Cell apoptosis, EdU (ethynyl deoxyuridine) and cell death detection assays were performed using the Annexin V-FITC/PI Kit (FXP018, 4Abio, China), BeyoClick™ EdU-594 Kit (C0078, Beyotime, China) and Calcein/PI Cell Viability/Cytotoxicity Assay Kit (C2015, Beyotime, China), respectively, according to the manufacturers’ instructions.

**Establishment of human BCa organoids**

After being washed with PBS, BCa tissues were minced into pieces and digested in Adv DMEM/F-12 (Thermo Fisher, 12634028) containing 5 mg/mL collagenase type II (LS004176, Worthington), 10 μg/mL DNAse I (LS002138, Worthington) and 10 μM ROCK inhibitor (Y-27632) at 37 °C for 30 minutes. Subsequently, cell aggregates were filtered through a 100 μm strainer, centrifuged at 300 × g for 5 minutes, and suspended in cold Matrigel (3533-010-02, R&D). Cell suspensions were then seeded in prewarmed 48-well plates at a volume of 25 μl per well, solidified at 37 °C for 10 minutes, and added to 200 μl of culture media. For passaging, organoids were either scraped with a pipette tip or digested using TrypLE™ Express (12605036, Invitrogen) with Y-27632 at 37 °C for 5 minutes.
