# Supplementary material for: UBE2S interacting with TRIM21 mediates the K11-linked ubiquitination of LPP to promote the lymphatic metastasis of bladder cancer
Source: Cell Death Dis. 2023 Jul 8;14(7):408. doi: 10.1038/s41419-023-05938-2 (PMC10329682; doi:10.1038/s41419-023-05938-2)
Supplement: Supplementary file 3 — Original Data File [file 41419_2023_5938_MOESM3_ESM.pdf]

Figure 2A

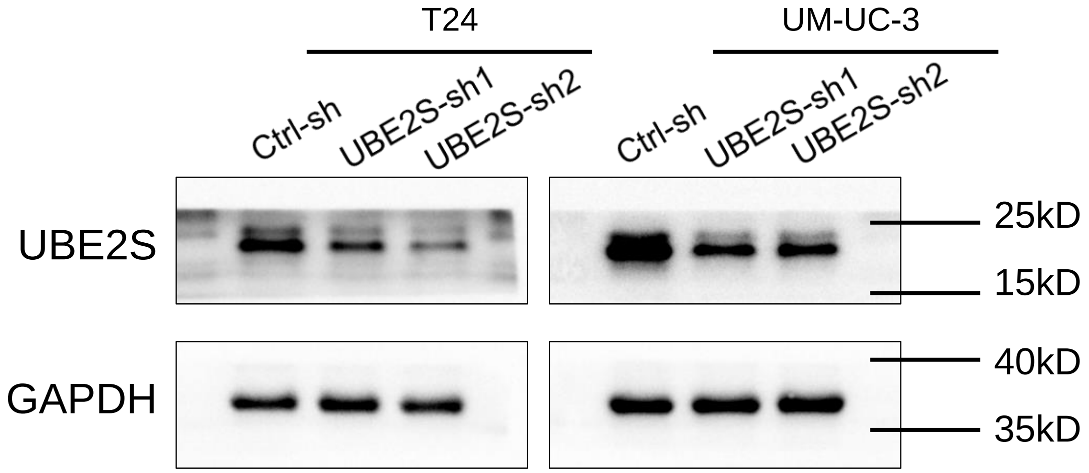

Figure 3A

Left

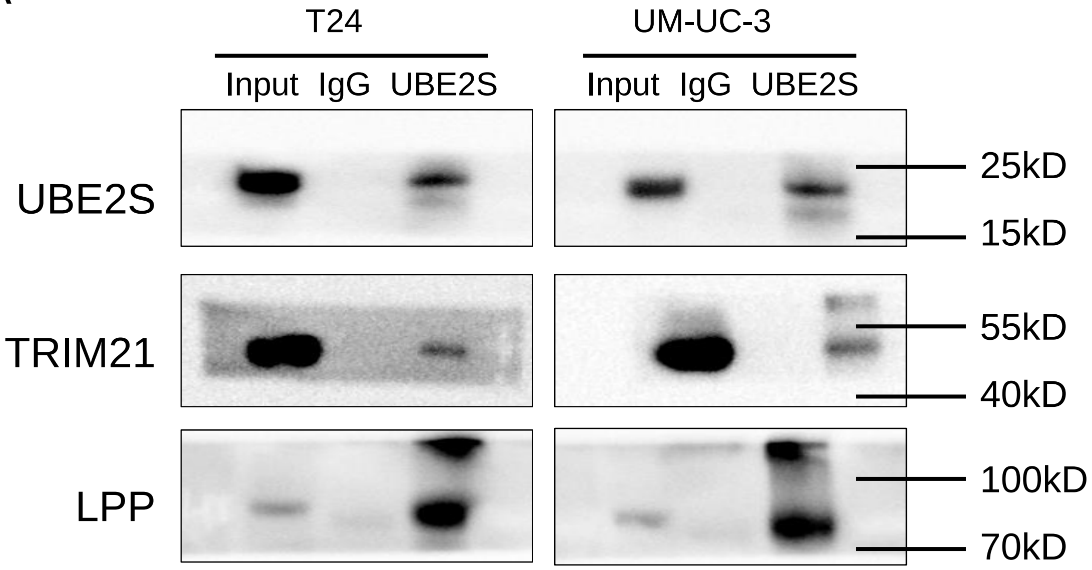

Figure 3A

Middle

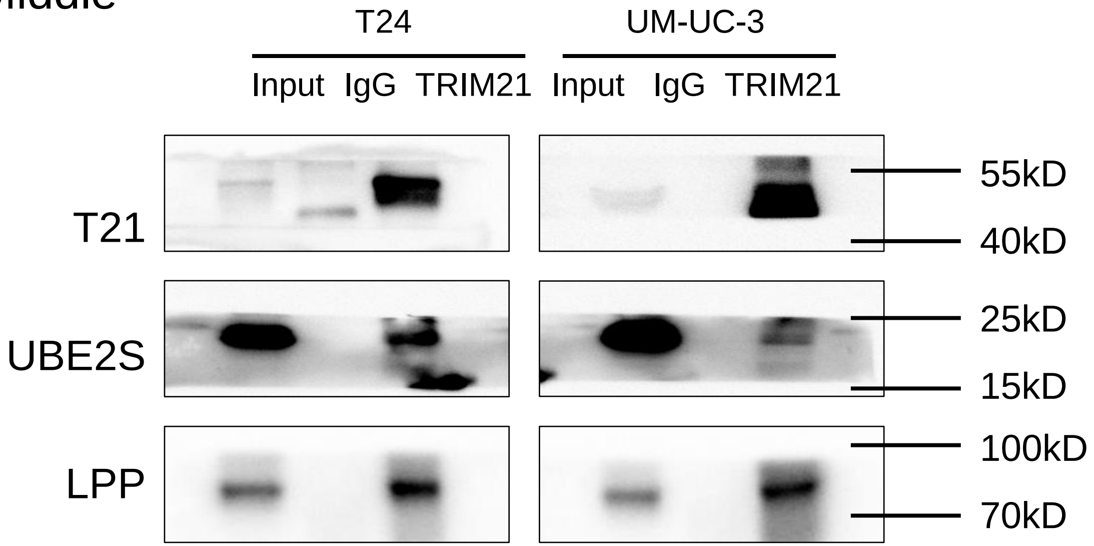

Figure 3A

Right

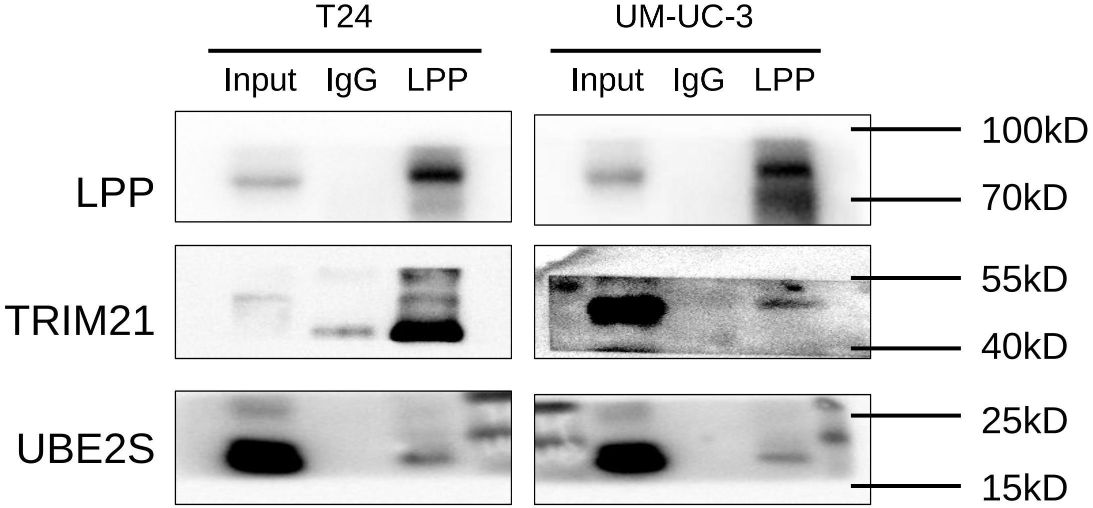

Figure 3C

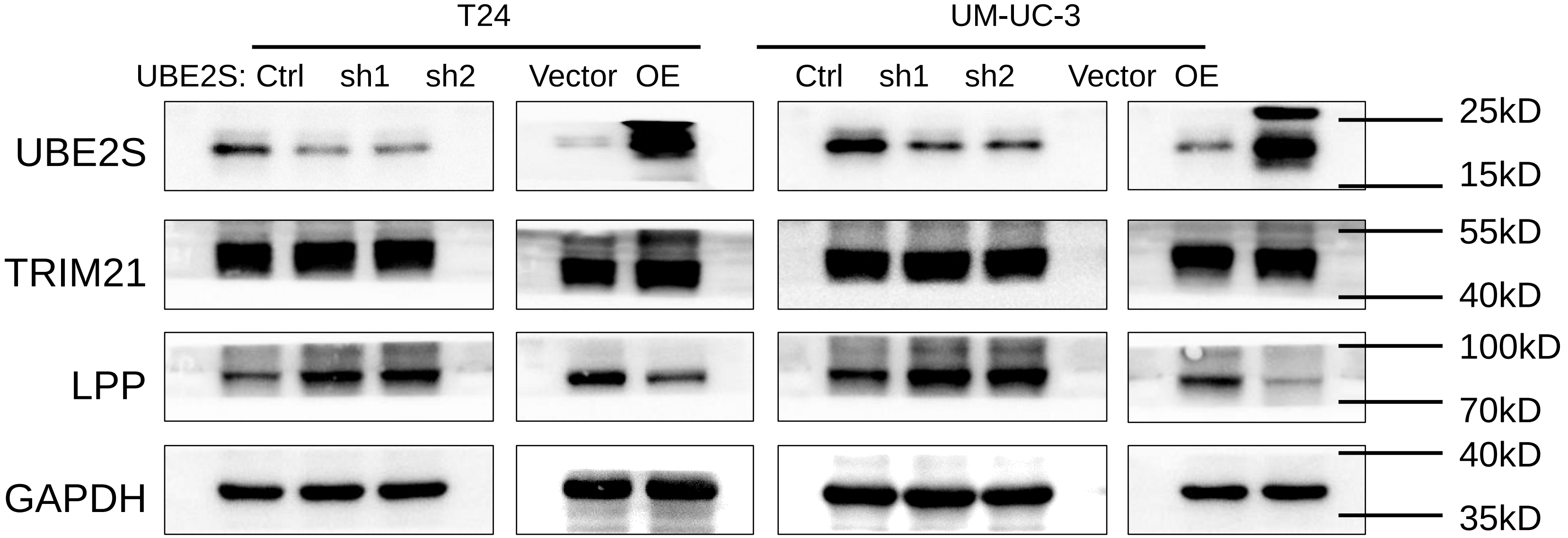

Figure 3D

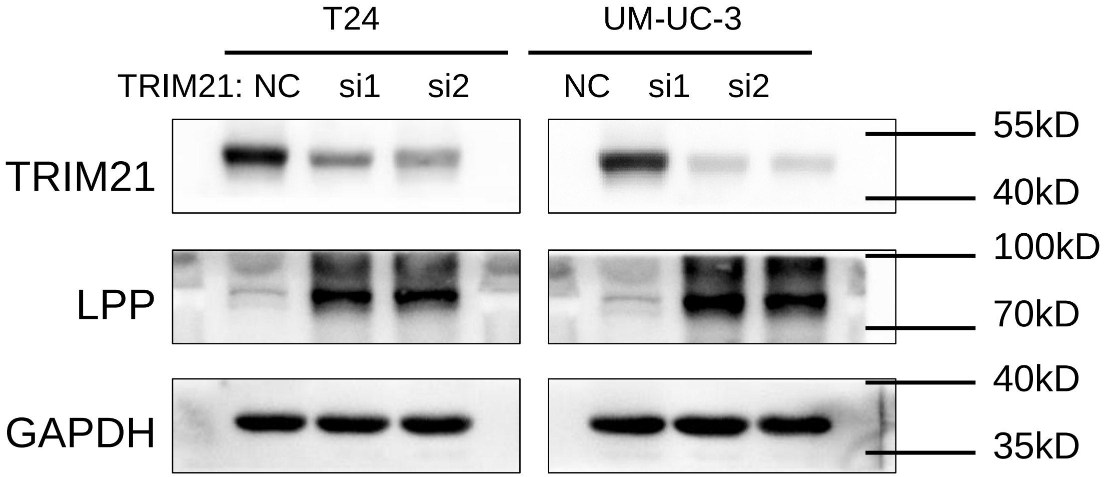

Figure 4A

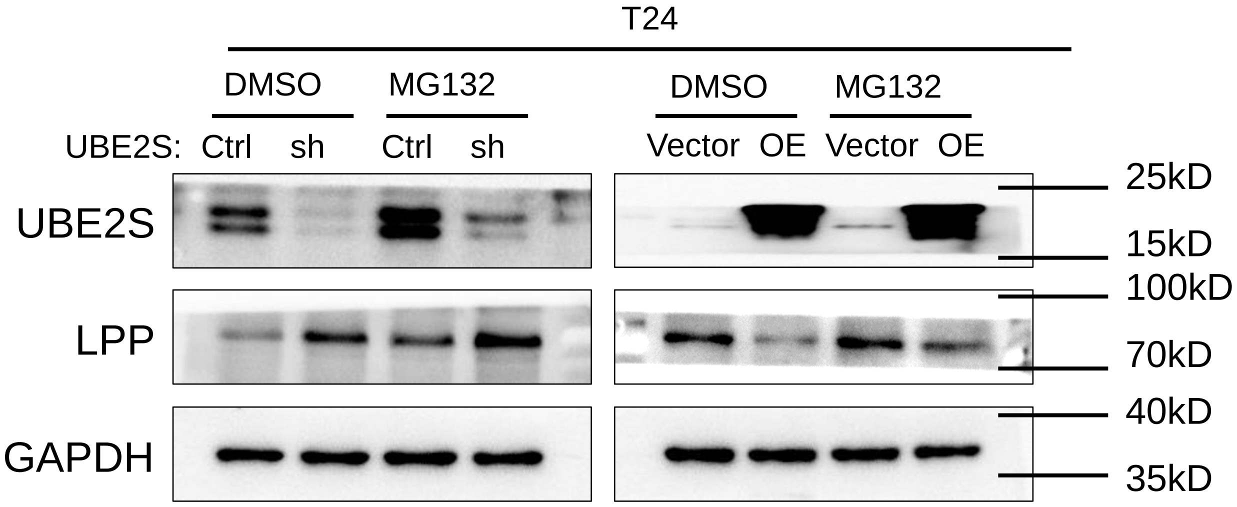

Figure 4B

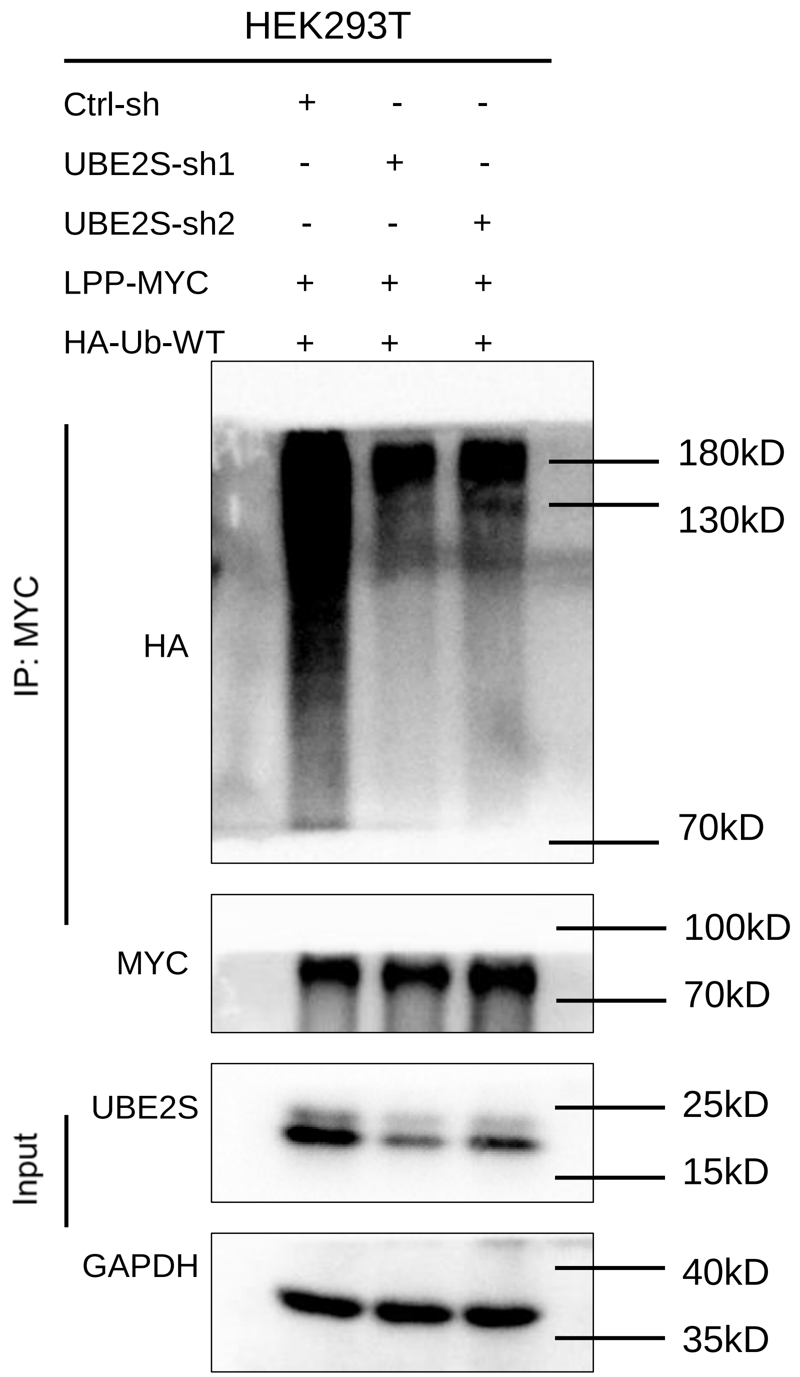

Figure 4C

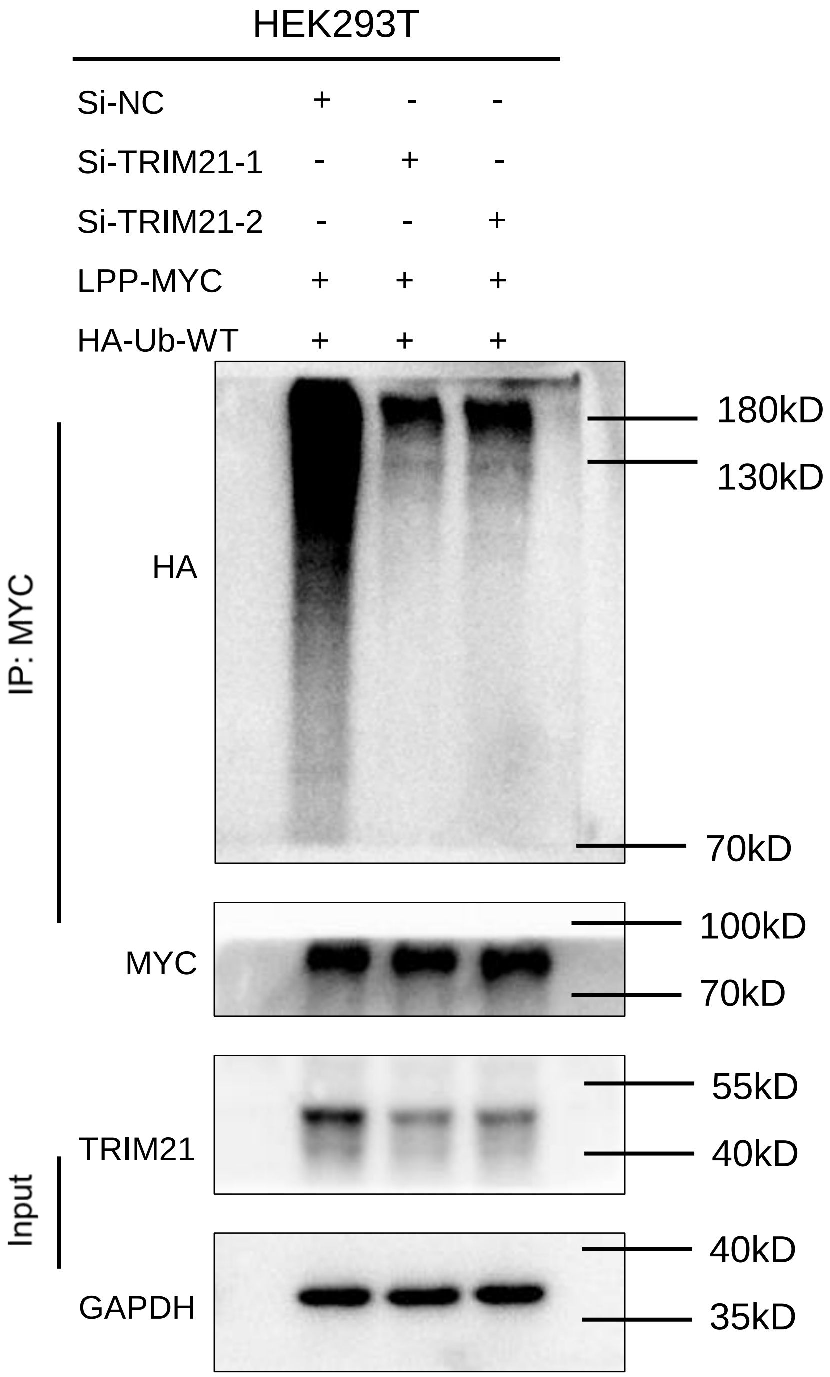

Figure 4D

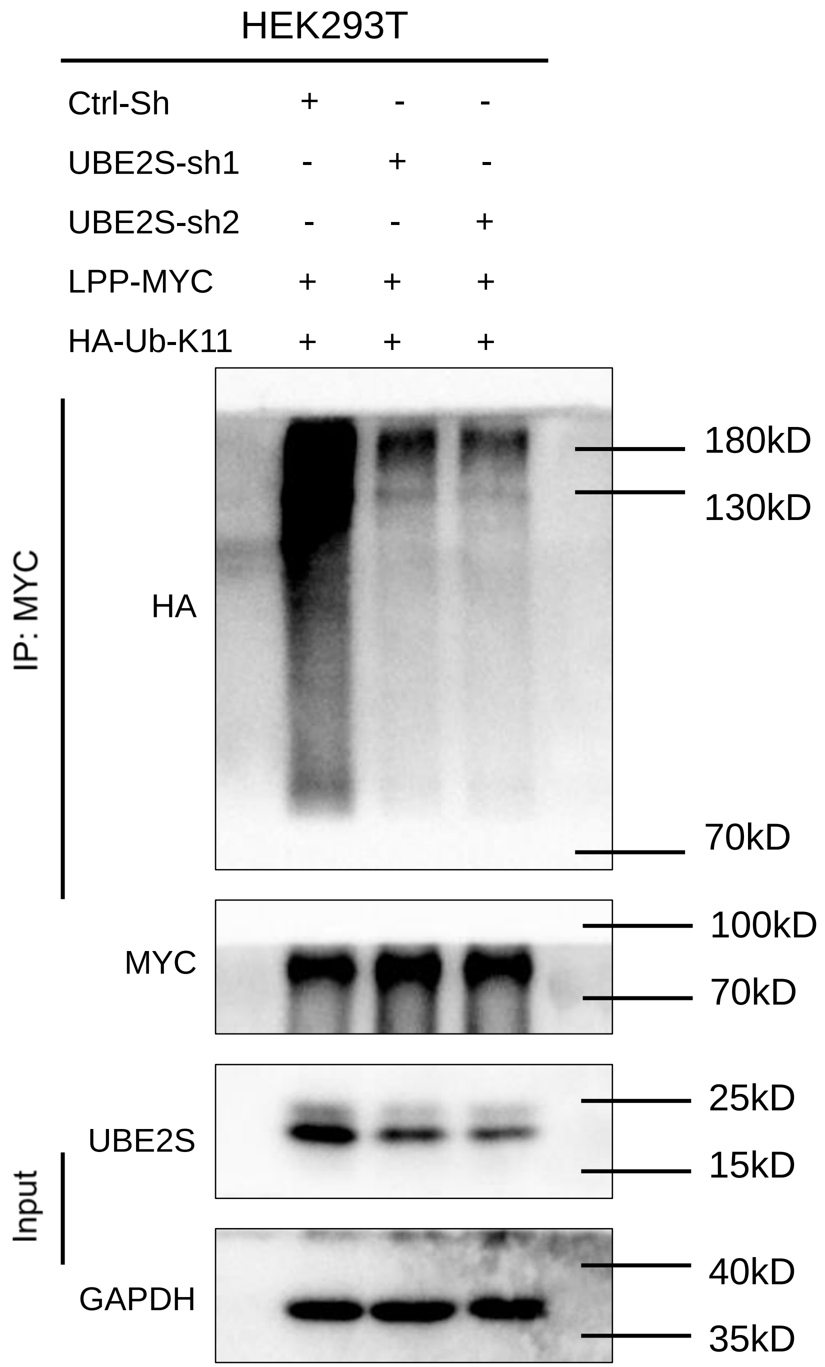

Figure 4E

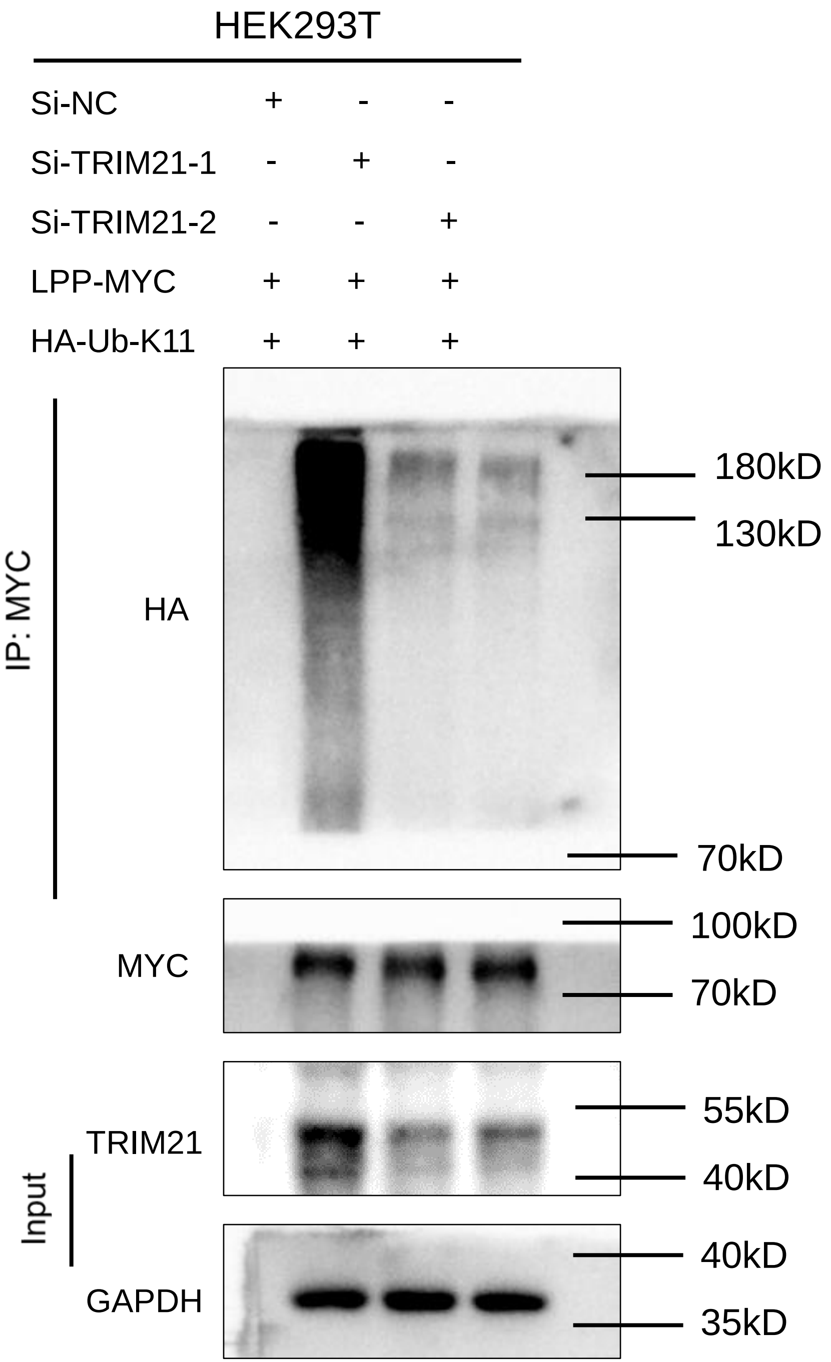

Figure 4F

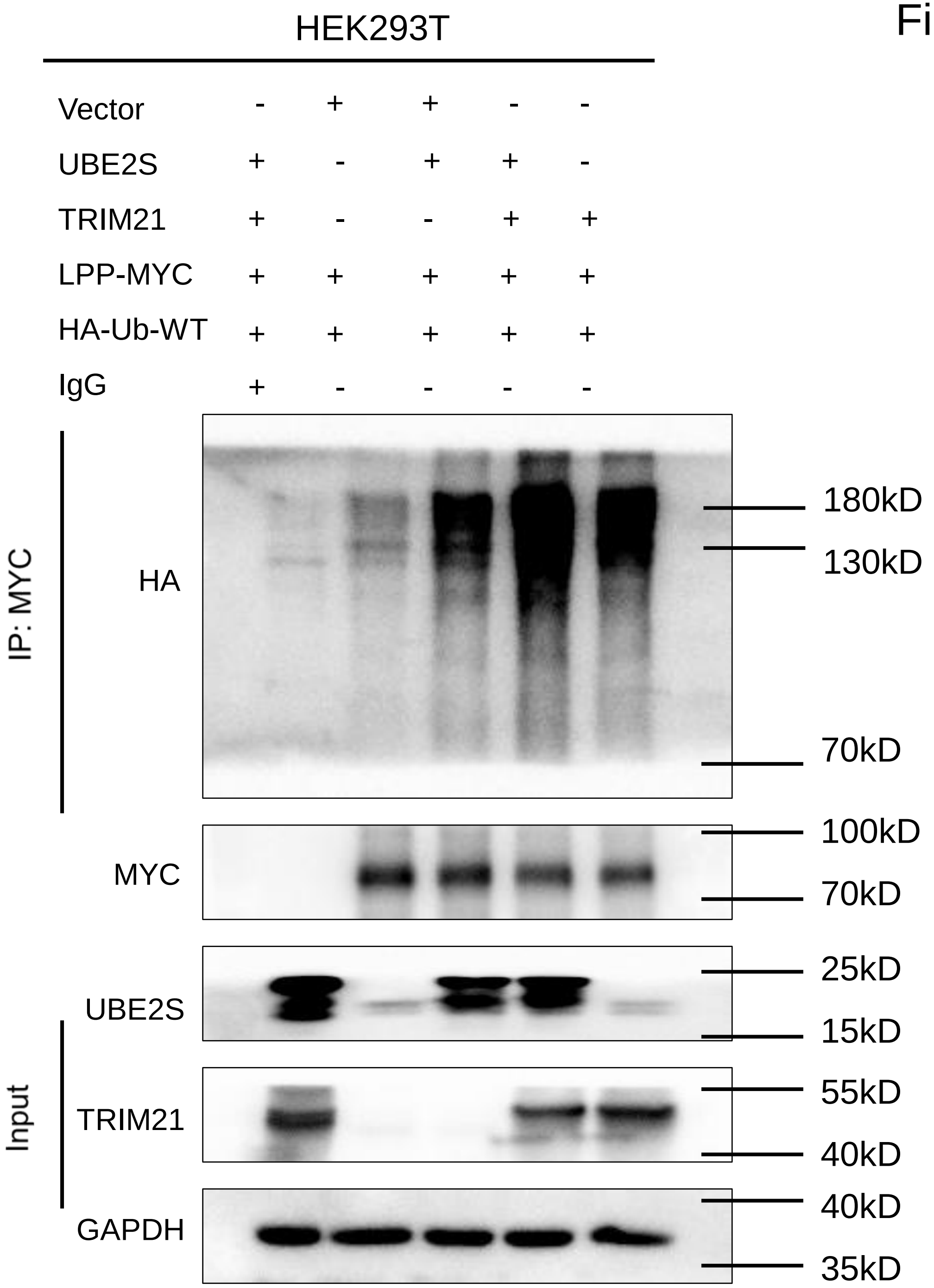

Figure 4G

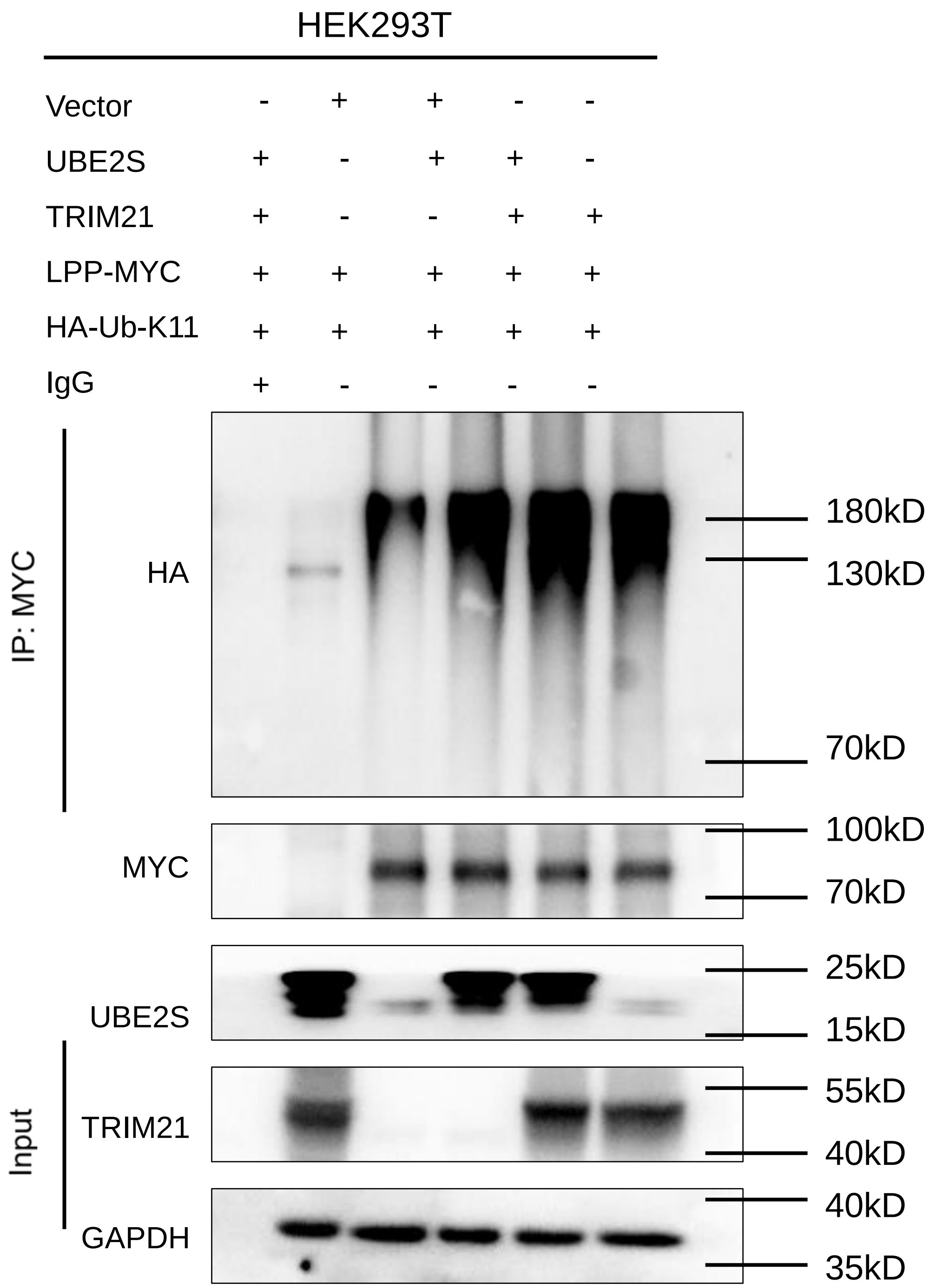

Figure 4H

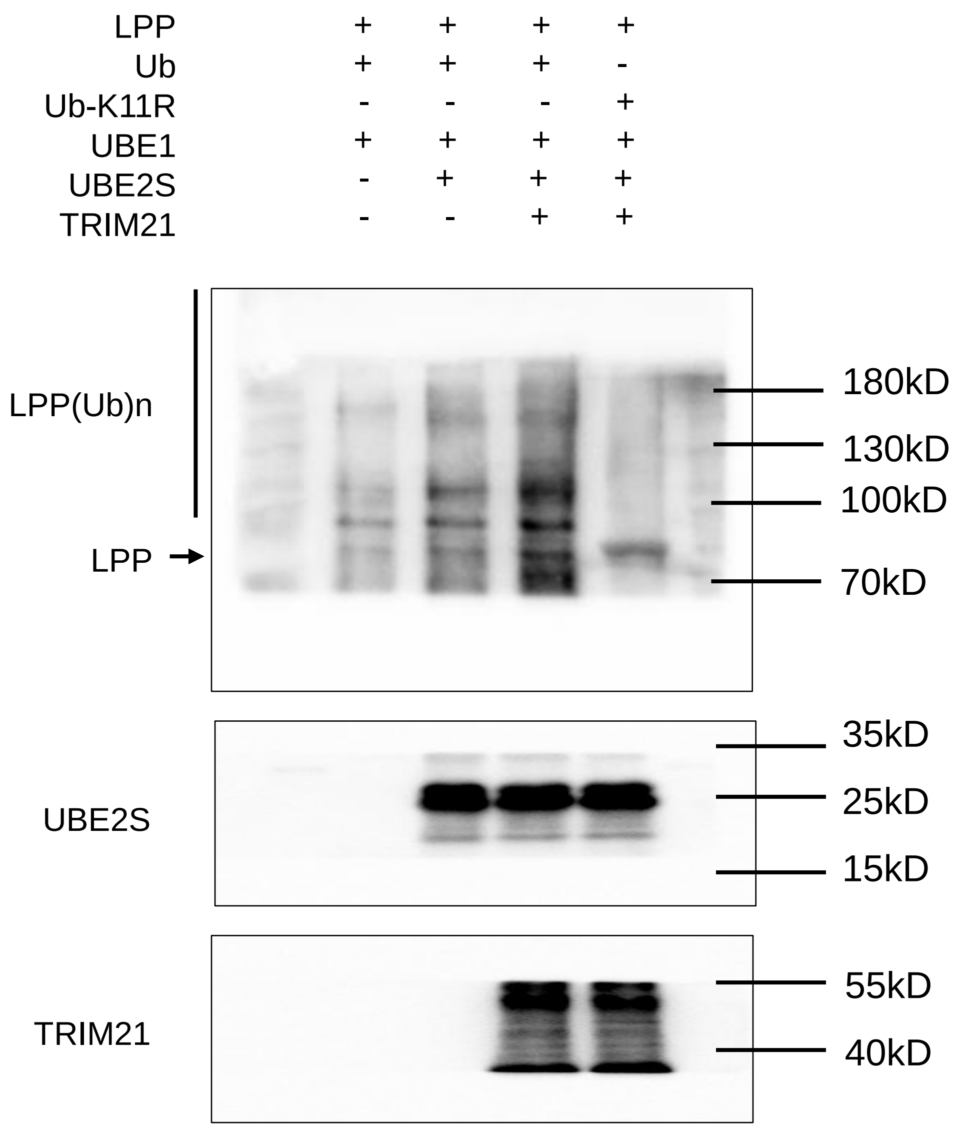

Figure 4J

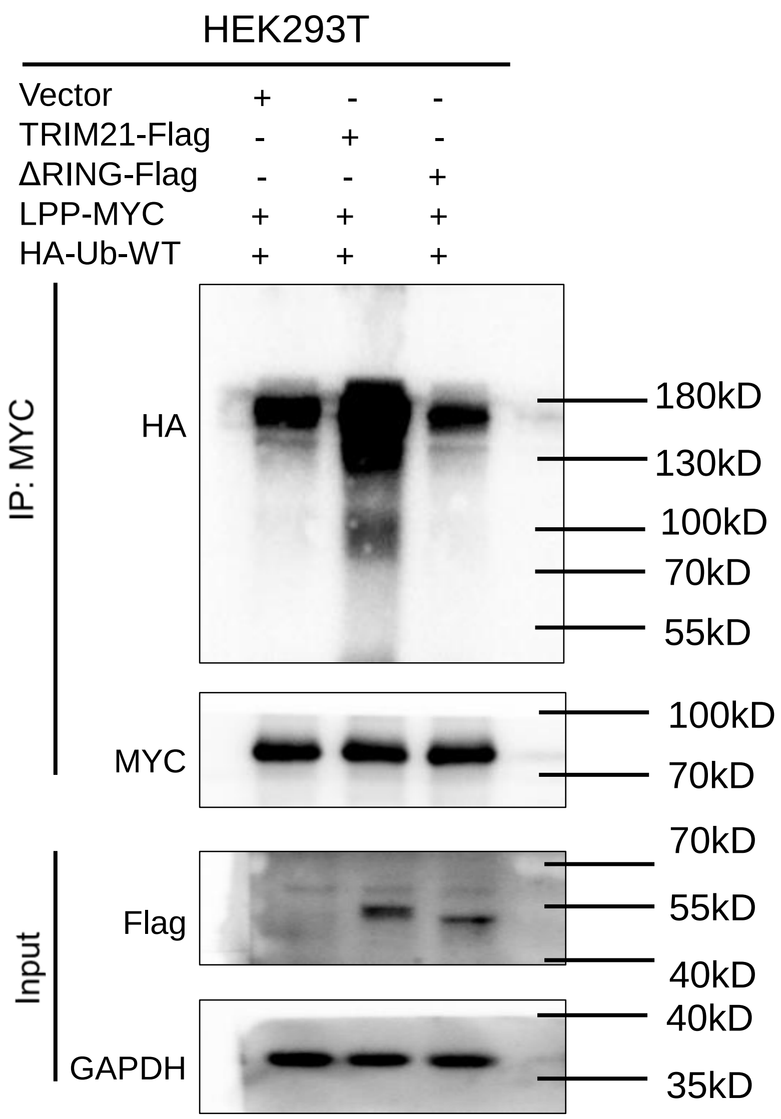

Figure 4K

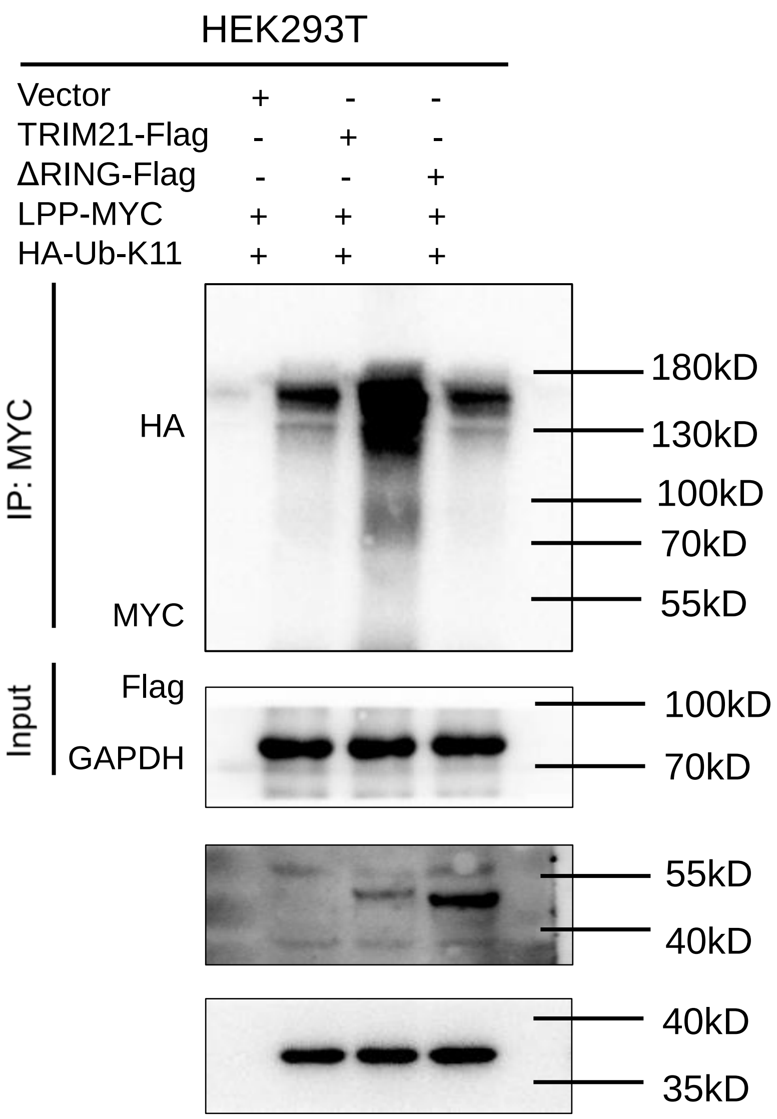

Figure 5G

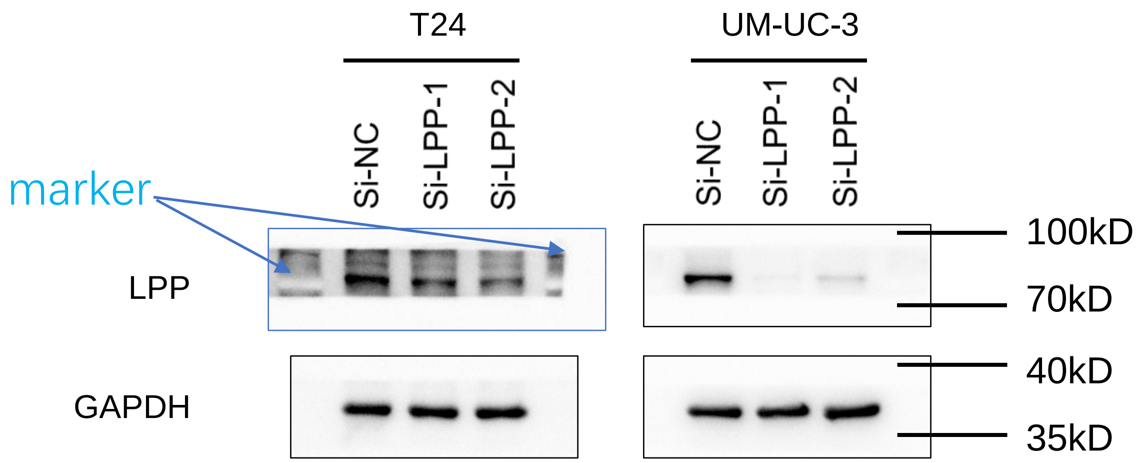

Figure 6A

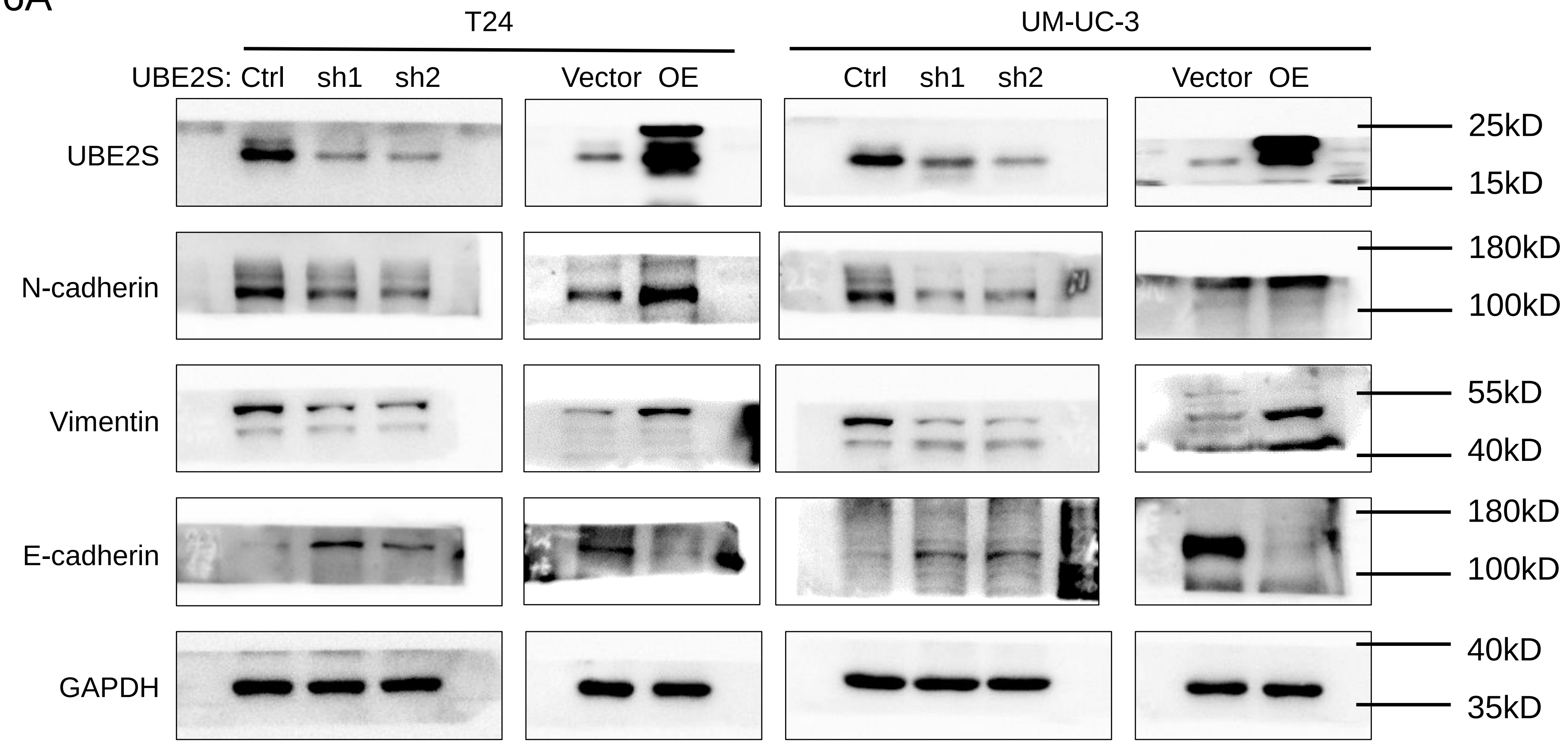

Figure 6B

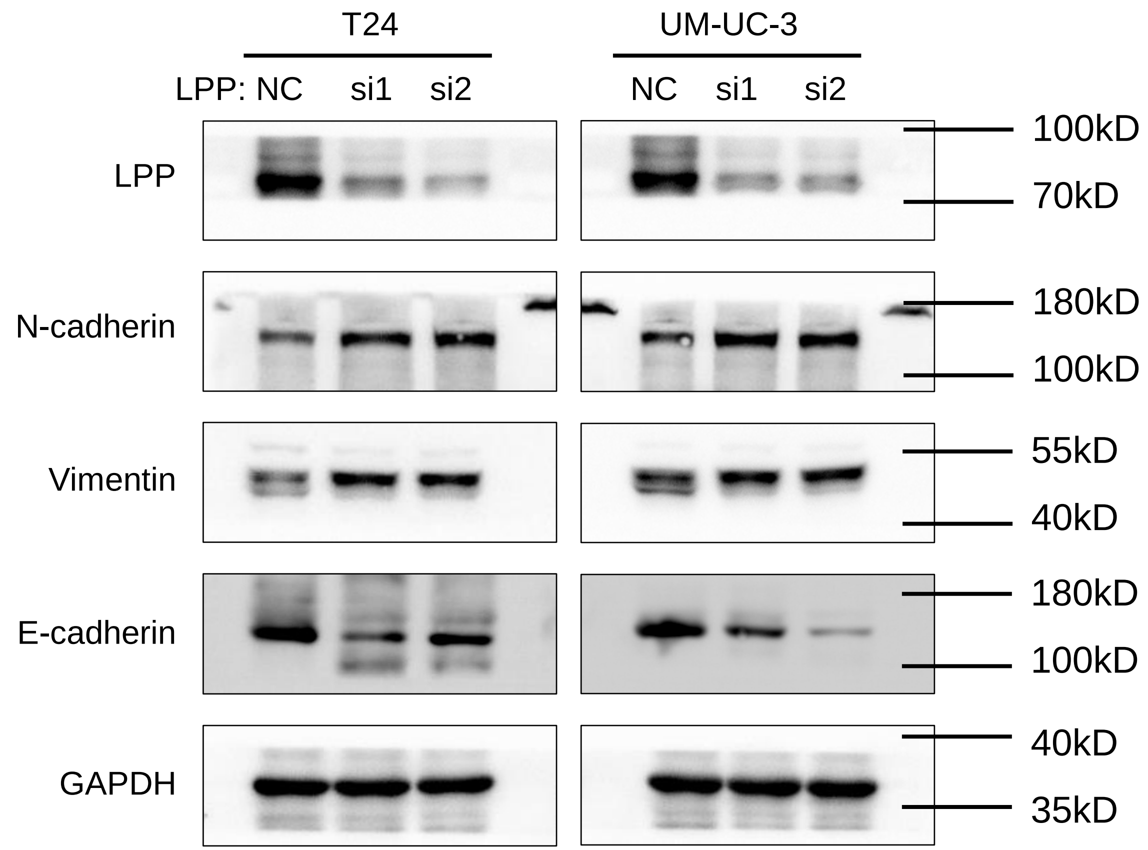

Figure 6E

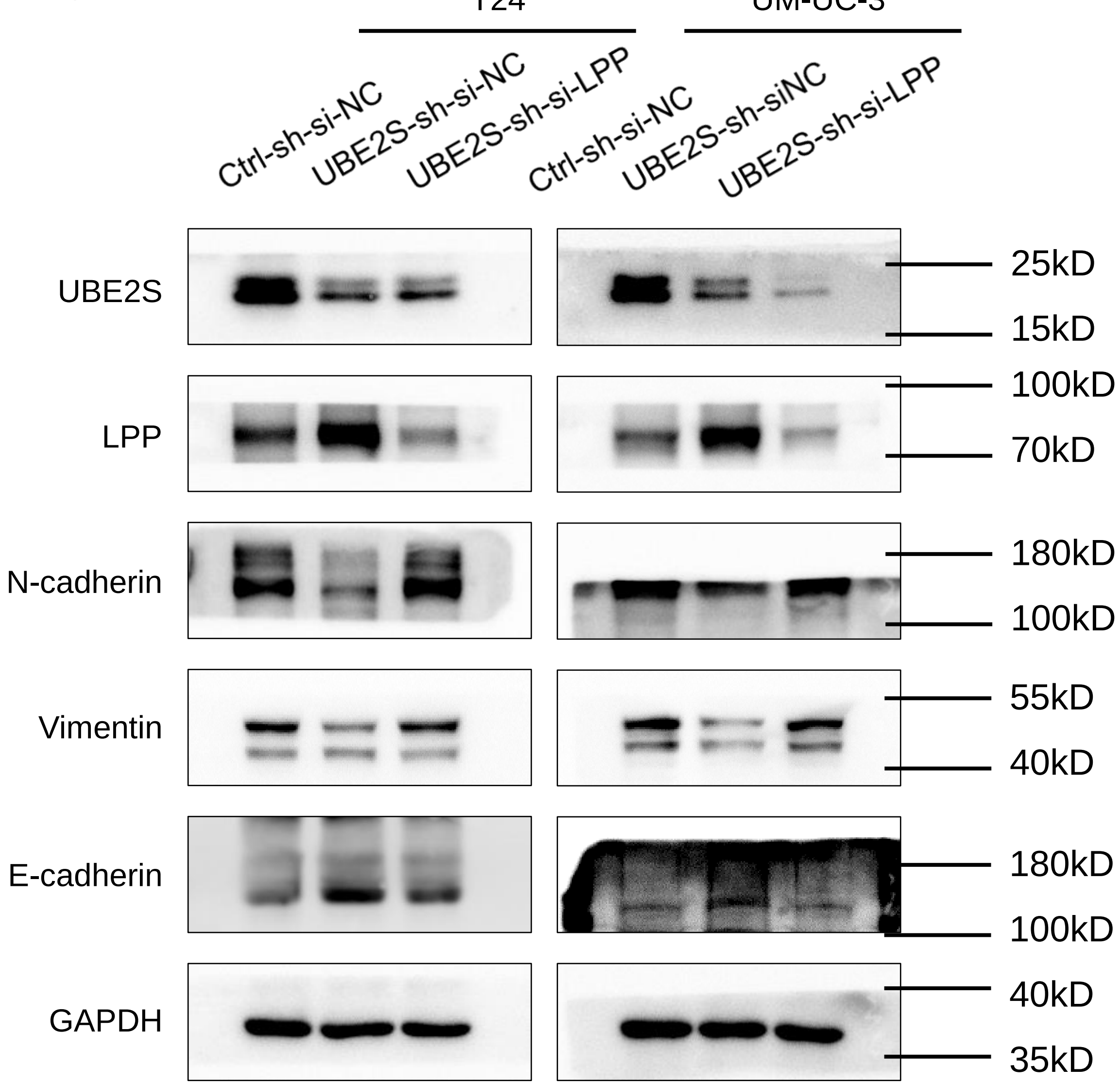

Figure 7A

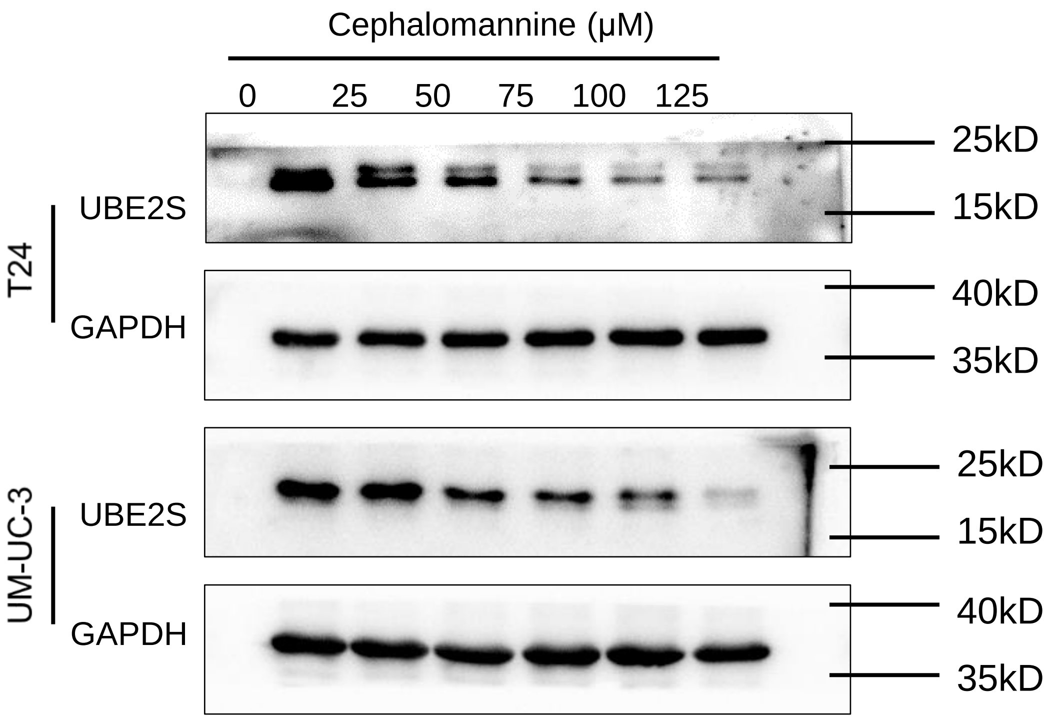

Cephalomannine (μM)
0
25
50
75
100
125
UBE2S
GAPDH
T24
UBE2S
GAPDH
UM-UC-3
25kD
15kD
40kD
35kD
25kD
15kD
40kD
35kD

Figure S2

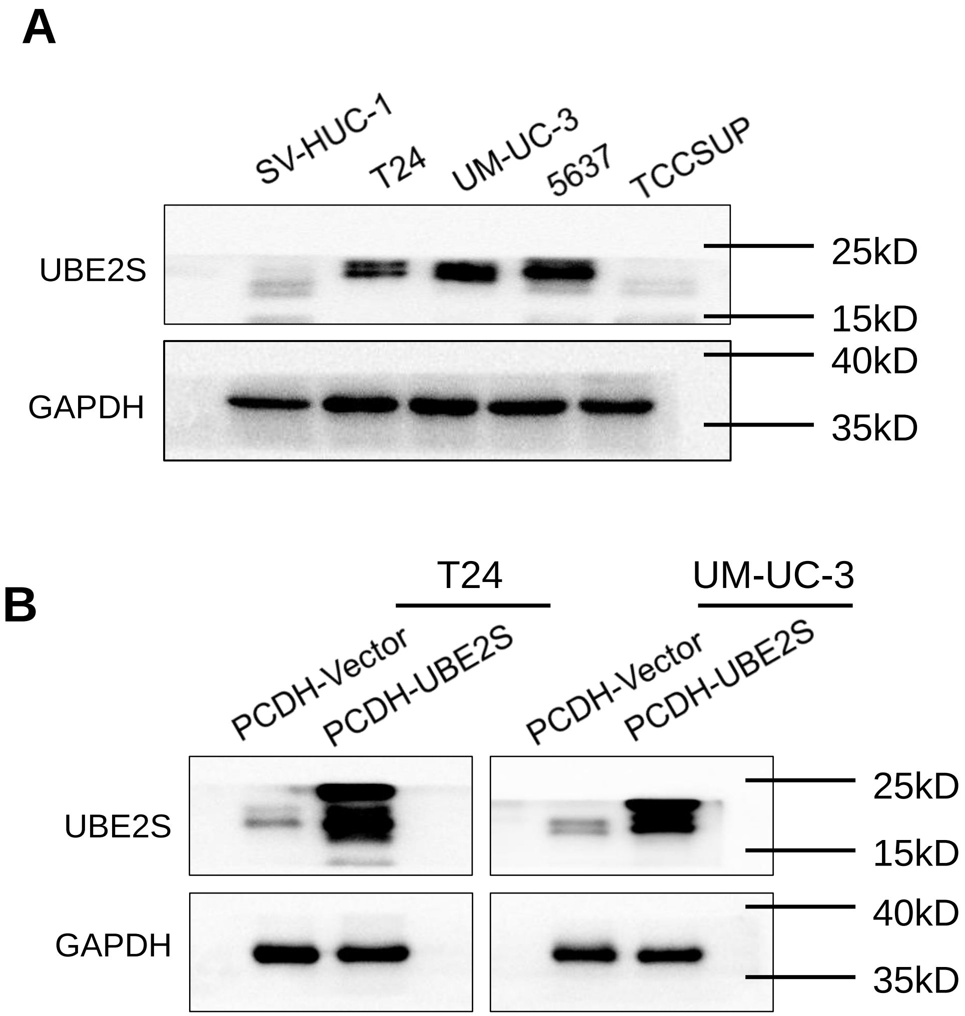

Figure S5

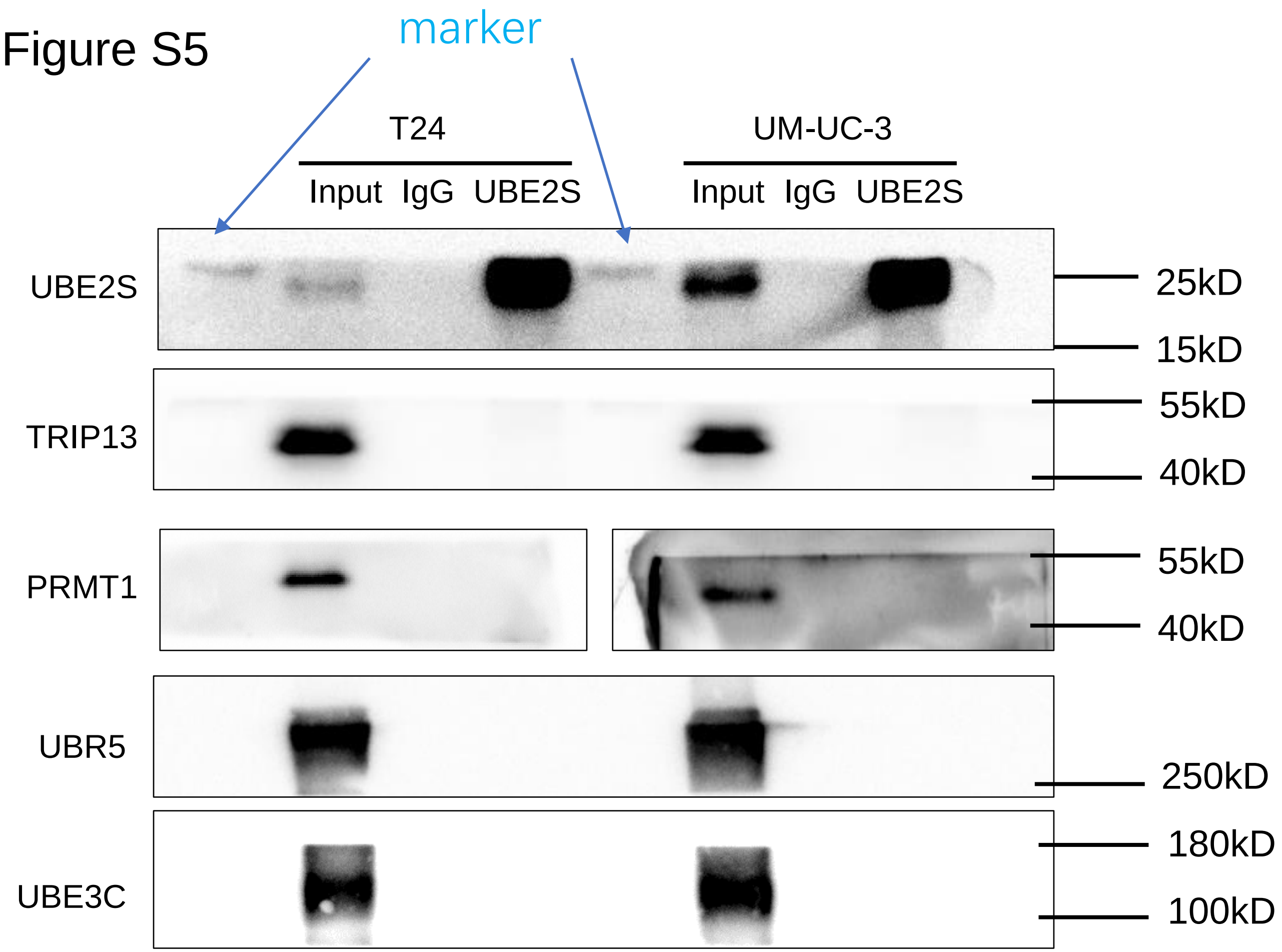

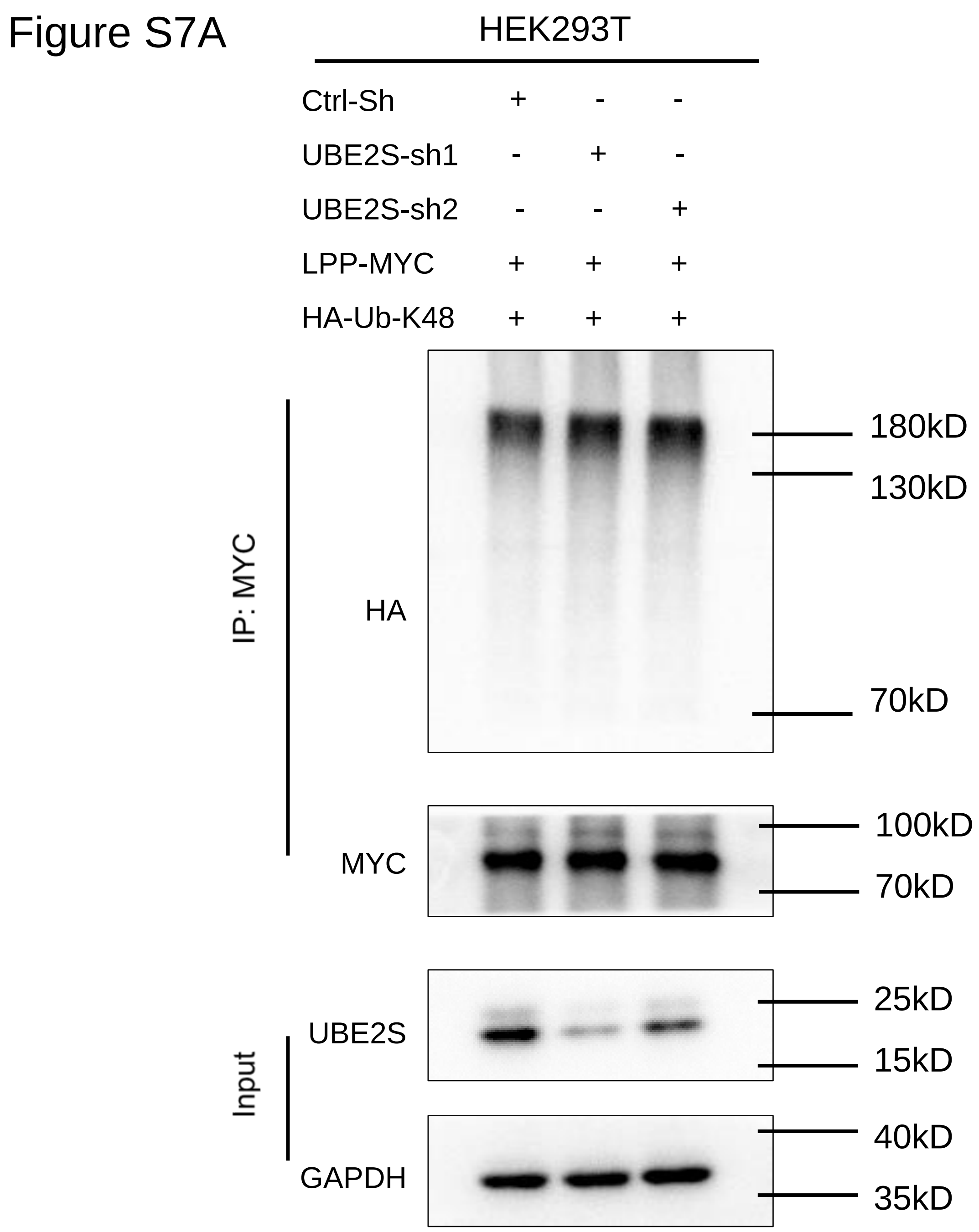
Figure S7A
HEK293T
Ctrl-Sh + - -
UBE2S-sh1 - + -
UBE2S-sh2 - - +
LPP-MYC + + +
HA-Ub-K48 + + +
IP: MYC
HA
180kD
130kD
70kD
MYC
100kD
70kD
Input
UBE2S
25kD
15kD
GAPDH
40kD
35kD

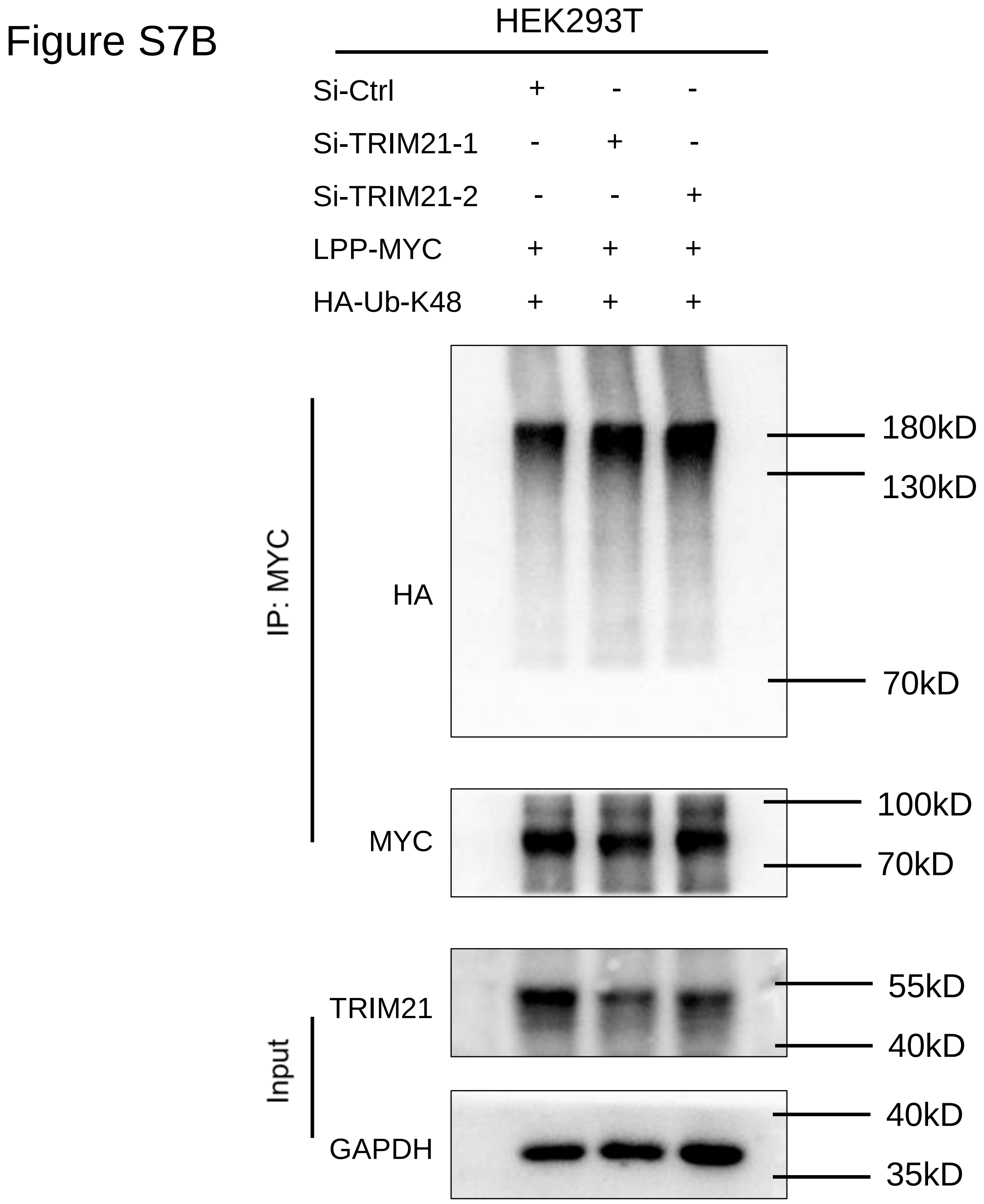
Figure S7B
HEK293T
Si-Ctrl + - -
Si-TRIM21-1 - + -
Si-TRIM21-2 - - +
LPP-MYC + + +
HA-Ub-K48 + + +
IP: MYC
HA
180kD
130kD
70kD
MYC
100kD
70kD
Input
TRIM21
55kD
40kD
GAPDH
40kD
35kD

Figure S7C

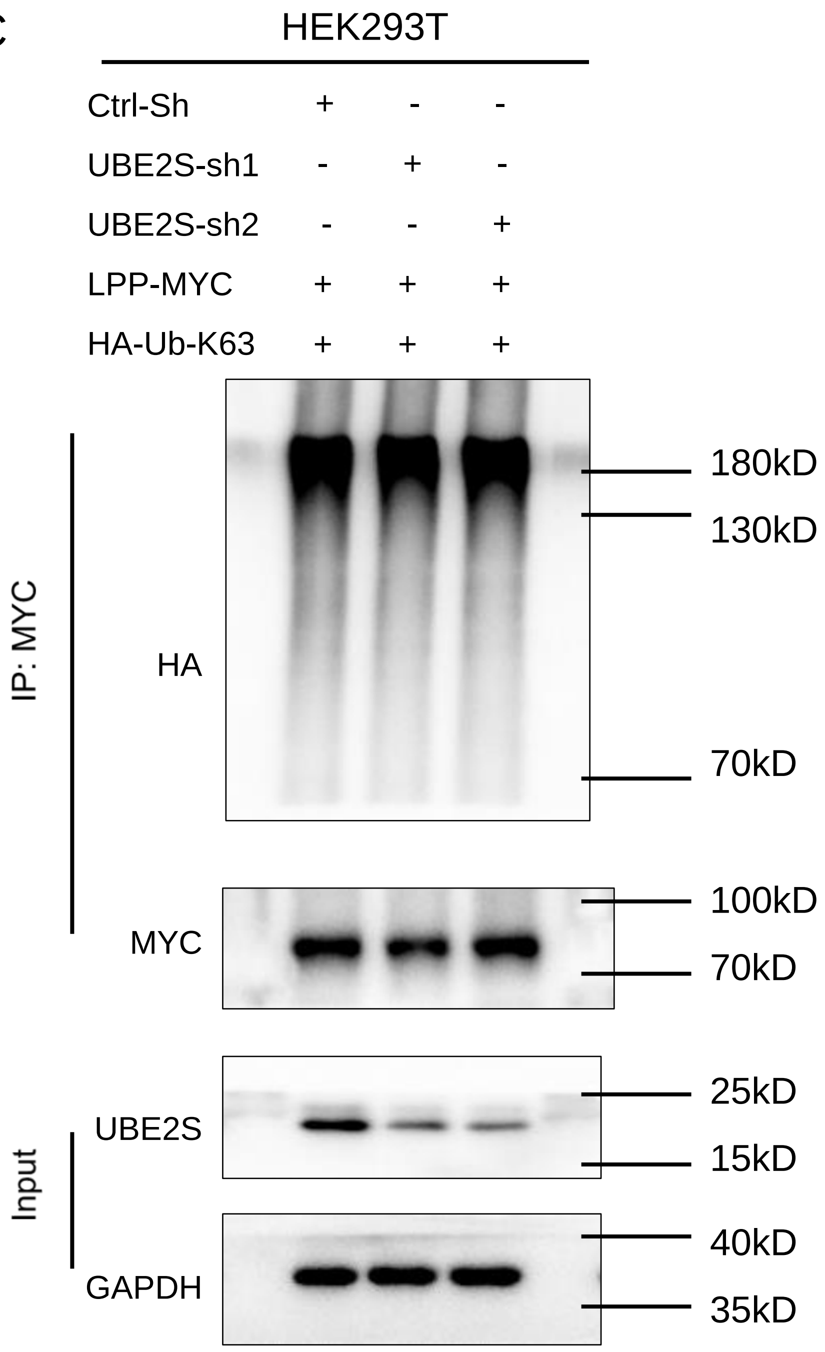

Figure S7D

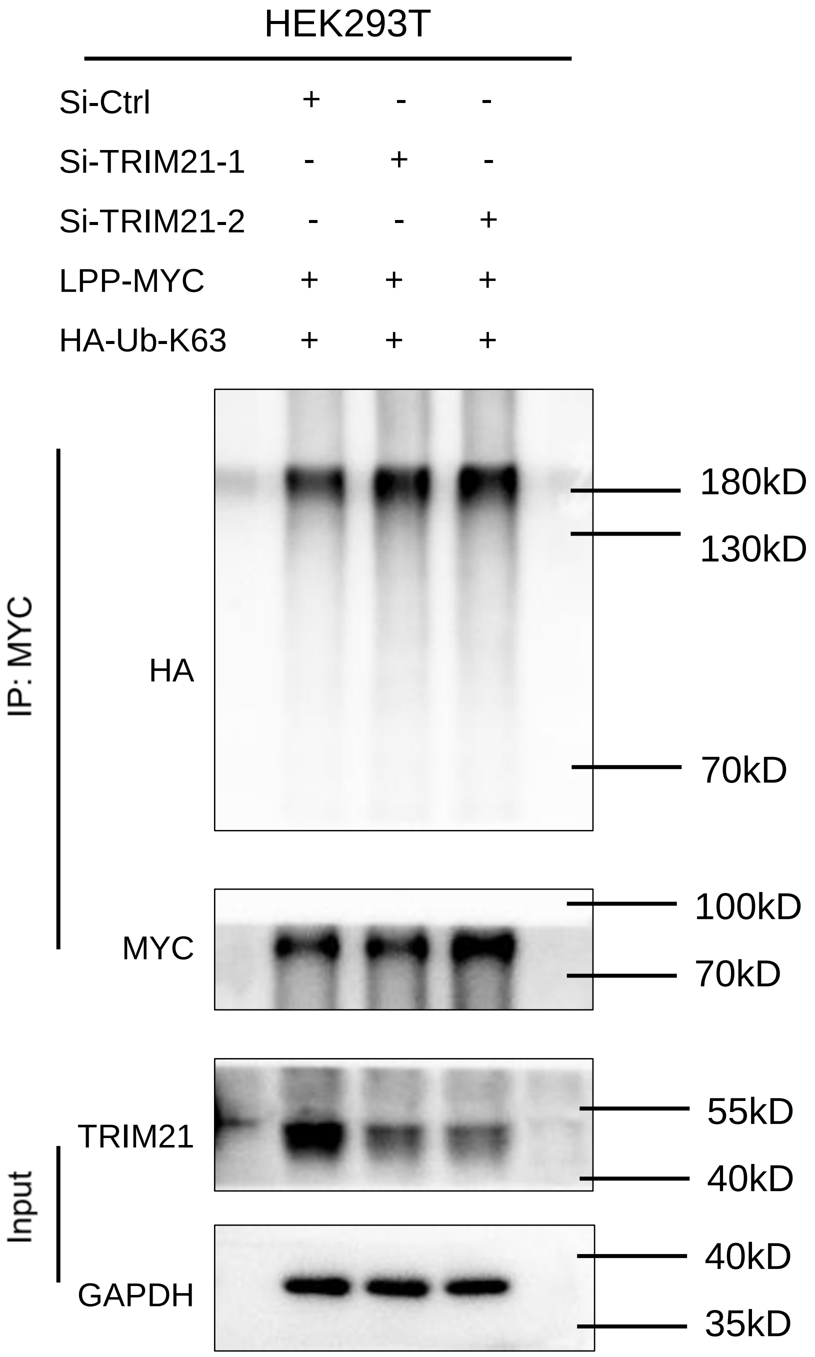

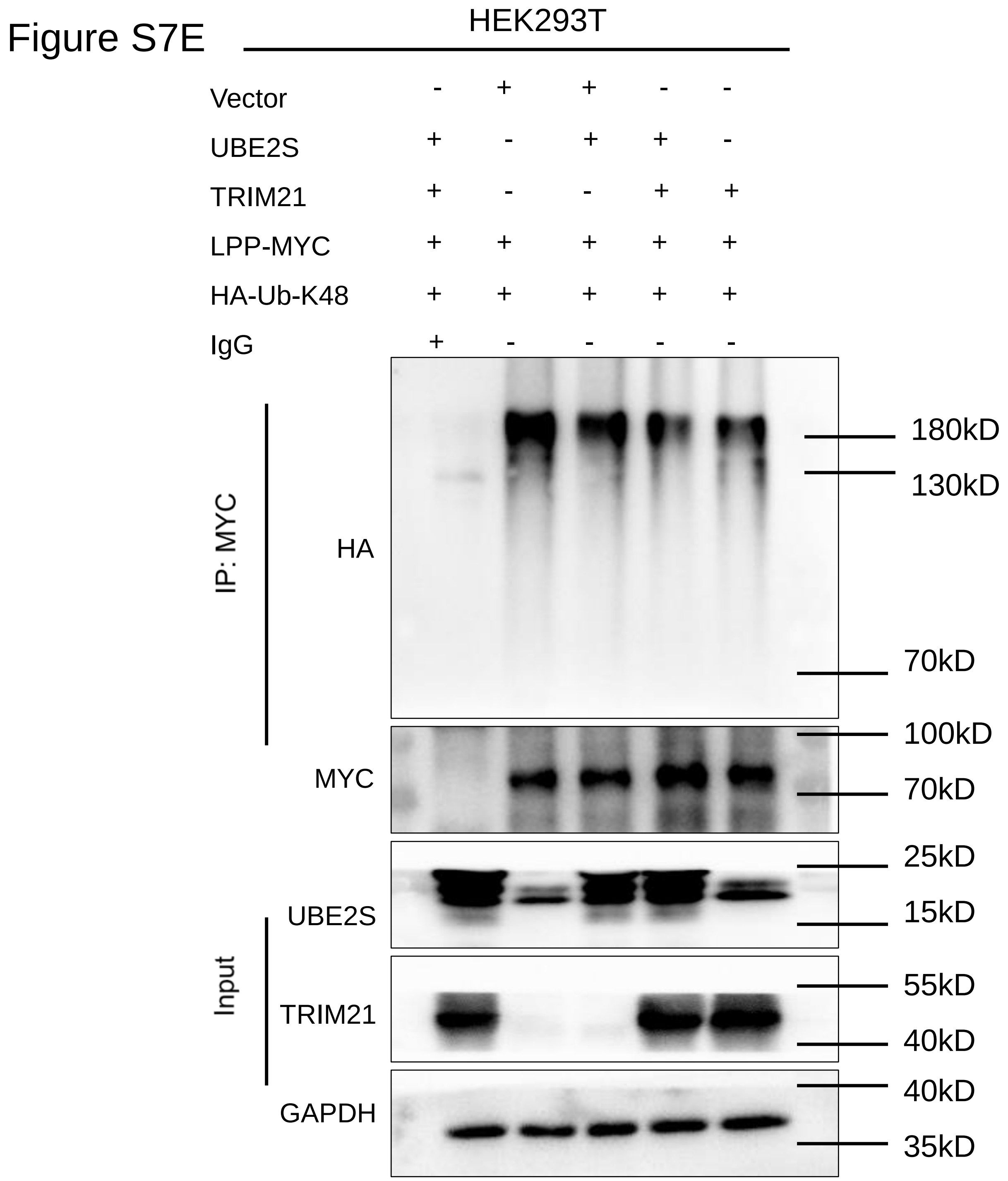
Figure S7E
HEK293T
Vector - + + - -
UBE2S + - + + -
TRIM21 + - - + +
LPP-MYC + + + + +
HA-Ub-K48 + + + + +
IgG + - - - -
IP: MYC
HA
180kD
130kD
70kD
MYC
100kD
70kD
Input
UBE2S
25kD
15kD
TRIM21
55kD
40kD
GAPDH
40kD
35kD

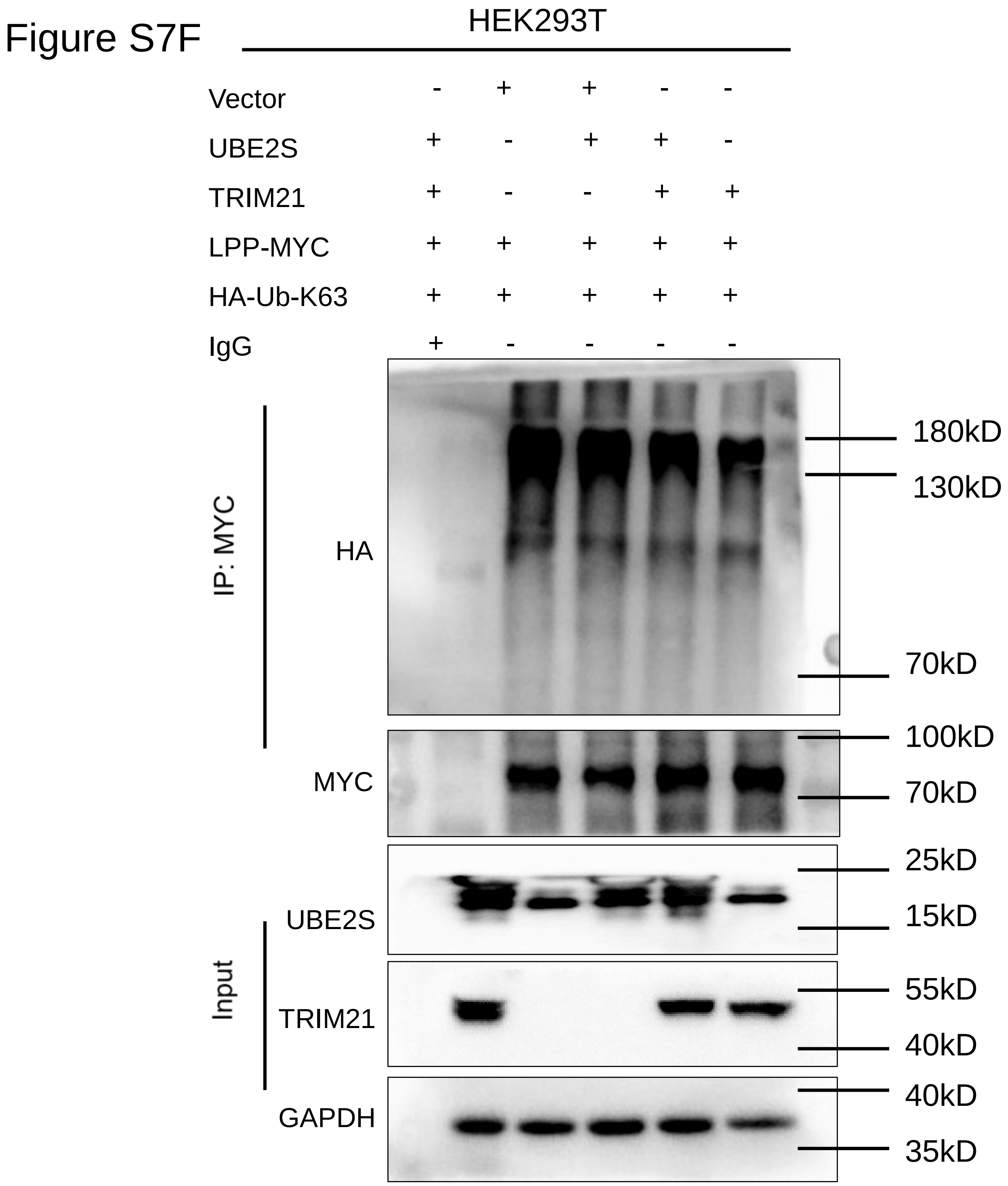
Figure S7F
HEK293T
Vector - + + - -
UBE2S + - + + -
TRIM21 + - - + +
LPP-MYC + + + + +
HA-Ub-K63 + + + + +
IgG + - - - -
IP: MYC
HA
180kD
130kD
70kD
MYC
100kD
70kD
Input
UBE2S
25kD
15kD
TRIM21
55kD
40kD
GAPDH
40kD
35kD
